# Antioxidant Activity of Essential Oils from Pinaceae Species

**DOI:** 10.3390/antiox13030286

**Published:** 2024-02-26

**Authors:** Robert Ancuceanu, Adriana Iuliana Anghel, Marilena Viorica Hovaneț, Anne-Marie Ciobanu, Beatrice Elena Lascu, Mihaela Dinu

**Affiliations:** 1 Department of Pharmaceutical Botany and Cell Biology, Faculty of Pharmacy, Carol Davila University of Medicine and Pharmacy, 020956 Bucharest, Romania; robert.ancuceanu@umfcd.ro (R.A.); marilena.hovanet@umfcd.ro (M.V.H.); beatrice-elena.lascu@drd.umfcd.ro (B.E.L.); mihaela.dinu@umfcd.ro (M.D.); 2 Department of Drug Analysis, Faculty of Pharmacy, Carol Davila University of Medicine and Pharmacy, 020956 Bucharest, Romania; anne.ciobanu@umfcd.ro

**Keywords:** Pinaceae, *Pinus*, *Picea*, *Abies*, *Cedrus*, DPPH, ABTS, FRAP, TBARS, beta-carotene bleaching assay

## Abstract

With a widespread distribution throughout the Northern Hemisphere and 11 genera, Pinaceae is the largest family of *Gymnosperms* in the world. Essential oils are an important chemotaxonomic marker for the species of this family, although the degree of chemical and biological investigation has not been the same for all genera. Essential oils from *Abies* and *Cedrus* (from the abietoid clade) or *Pinus* and *Picea* (from the pinoid clade) have been more extensively investigated with respect to their chemical composition and biological or pharmacological properties, including their antioxidant effects. Instead, essential oils from the other genera of the family have been less explored in this respect or even have not been investigated at all. This is a narrative review looking into the knowledge acquired up to date, the variability and limitations of the current methods used to estimate antioxidant effects, and multiple comparisons between EOs obtained from different genera, species, and plant parts, as well as potential applications and future directions of research and utilization of essential oils derived from Pinaceae species.

## 1. Introduction

The Pinaceae family is one of the most important groups of gymnosperms (conifers) and is currently recognized as consisting of 11 genera and over 260 species (plus 44 subspecies) scattered throughout the northern part of the planet, forming the largest membership of the mountain forest ecosystems on this territory [1,2]. Its division into two main clades, pinoid (*Cathaya*, *Larix*, *Picea*, *Pinus*, and *Pseudotsuga*) and abietoid (*Abies*, *Cedrus*, *Keteleeria*, *Tsuga*, *Nothotsuga*, and *Pseudolarix*), seems well supported by phylogenetic data, despite uncertainties or controversies concerning the placement of four of its eleven genera [1,3] or the fact that in the past three or four subfamilies have been recognized and often are still used (Pinoideae Pilg., Abietoideae Pilg., Laricoideae Melchior et Werdermann, Piceoideae Frankis) [4,5,6]. A two-clade classification of the Pinaceae genera is also supported by multiple phenotypical characters, such as the wood structure, amount and placement of resin ducts inside the immature root’s vascular system, whether or not resin vesicles are present within the layers of the seed coat, and the immunological characteristics of the seed proteins [1].

Like many other gymnosperms, species of the Pinaceae family biosynthesize terpenoids, either in the form of oleoresins or essential oils. These compounds are believed to play a vital role in the host’s defense against various pathogens, insects, and herbivores [7]. Terpenic compounds from essential oils are recognized as good chemotaxonomic markers of particular usefulness in studying species belonging to the order Pinales, and Pinaceae are one of the primary families in this order [8]. The presence of schizogenic oil ducts is considered specific for the family [9].

Essential oils (EOs) are complex and diverse mixtures of natural compounds with a low boiling point (hence their volatile character, being susceptible to removal by distillation), lipophilic properties, and relatively low molecular weights (under 300 Da) [10]. Although rarely used as active ingredients of conventional medicines or investigated in adequately designed clinical trials, EOs are widely believed to have a wide range of health benefits, such as antibacterial, antiviral, or antifungal properties [11], providing stress and anxiety relief [12,13], improving sleep disorders [14], mitigating cognitive deficits in Alzheimer’s disease [15], and improving other conditions affecting the central nervous system [16]; they also have health benefits for cardiovascular [17], inflammatory [18], gastrointestinal [19], immunological [20], hepatic [21], oncological [22], and other diseases [23].

In the attempts to justify some of the biological activities of EOs, their antioxidant abilities are often assumed to be of primary importance, an assumption based on the role played by oxidative stress in various pathological processes [24]. An increasing volume of data supports the possibility that free radical-induced cellular damage is the root cause of many illnesses [25]. However, this research is limited mainly to non-clinical (often in vitro) or, at best, clinical observational models. There is speculation that EOs could help prevent various diseases, including cancer, heart disease, cognitive dysfunction, or a weakened immune system, by scavenging free radicals [26]. In addition to these hypothetical health benefits, the antioxidant activities of EOs could contribute to their food and feed-preserving properties, with potential application in use as promising feedstuffs for farm animals, resulting in animal products with better organoleptic properties and extended shelf lives [27].

Such antioxidant properties of EOs depend on their chemical composition and are attributable to ingredients with hydroxyl (particularly phenolic) groups or multiple bonds [28]. Today, over 3000 EOs are known, often with an impressive variability in their qualitative and quantitative composition [29]. Because Pinaceae species are par excellence producers of EOs (from multiple organs, with distinct chemical compositions), it is interesting to understand what is known about the antioxidant properties of their essential oils.

In this context, it is interesting to understand what is known about the antioxidant effects of the essential oils produced by Pinaceae species because this family constitutes a taxon known for its EO production, and these oils come from diverse organs and species with different chemical compositions. This paper is a narrative review based on primary bibliographic sources collected in a systematic manner from several databases: Pubmed, Web of Science Core Collection, Scopus, and Google Scholar, using the keywords “Pinaceae” + “essential oil” + “antioxidant”. Following this search strategy, which was applied similarly in each of the four databases and eliminated irrelevant papers, we ended up with seventy-nine publications containing primary data on at least one essential oil prepared from a Pinaceae species. We mainly analyzed the data reported by the authors of primary sources in the text and tables; in several cases, the authors have only provided plots without reporting the corresponding numbers. In such cases, we used the R package metaDigitise to convert the plots to the corresponding values [30].

Scientific names are often reported without the taxonomist(s) who formally described the species and attributed its name. We used the correct name when scientific names included typing errors (e.g., *Pinus halapensis* instead of *P. halepensis*).

## 2. Methods Available for Antioxidant Testing of Essential Oils

Over time, a range of methods have been proposed to assess the antioxidant effects of essential oils and plant extracts (as well as for different synthetic substances). They can be classified into two main groups: (i) chemical-based assays and (ii) enzyme-based assays [31].

Chemical-based assays, in turn, are sub-classified as assays based on single electron transfer (SET) reactions and hydrogen transfer atom (HAT) reactions [32,33,34].

SET-based methods evaluate the capacity of a putative antioxidant to reduce a substrate (organic molecule, free radical, or metal) by transferring a single electron; such a reduction reaction is accompanied by a change in color [32]. The following methods are considered in the literature to be based on SET reactions:

- TEAC (Trolox equivalence antioxidant capacity);
- FRAP (ferric ion reducing antioxidant power);
- Total antioxidant potential methods based on a Cu^2+^ complex used as an oxidant;
- DMPD^•+^ (N,N-dimethyl-p-phenylenediamine) radical scavenging;
- CUPRAC (Cupric ions reducing antioxidant power);
- Total phenolics assay by the Folin–Ciocâlteu reagent;
- TAC (total antioxidant capacity);
- Phosphomolybdenum scavenging;
- Scavenging of xanthine oxidase;
- DPPH^•^ (2,2-diphenyl-1-picrylhydrazyl) radical scavenging;
- ABTS^•+^ (2,2-azinobis 3-ethylbenzthiazoline-6-sulfonic acid radical scavenging [32,33,34,35,36].

HAT-based methods evaluate the capacity of a putative antioxidant to scavenge free radicals by transferring a hydrogen atom [32]. The following methods are considered in the literature to be based on HAT reactions:

- ORAC (oxygen radical absorbance capacity);
- TRAP (total radical trapping antioxidant parameter);
- Methods based on the inhibition of LDL oxidation;
- TOSC(A) (total oxyradical scavenging capacity);
- β-carotene bleaching methods;
- CBAs (crocin-bleaching assays);
- Chemiluminescent assay;
- Nitric oxide scavenging;
- TBARS (thiobarbituric acid reactive substances);
- Inhibited oxygen uptake;
- DPPH^•^ (2,2-diphenyl-1-picrylhydrazyl) radical scavenging;
- ABTS^•+^ (2,2-azinobis 3-ethylbenzthiazoline-6-sulfonic acid radical scavenging [32,33,34,35,36].

Chemical-based assays have also been classified depending on the nature of chemicals reduced by the antioxidants as follows:

- Radical scavenging assays (e.g., DPPH, ABTS, hydroxyl radical);
- Lipid peroxidation assays (e.g., β-carotene/linoleic acid bleaching assay, thiobarbituric acid reactive substances (TBARS));
- Reduction power assays (e.g., FRAP, CUPRAC, phosphomolybdenum assay) [31].

Enzyme-based assays evaluate the impact of a putative antioxidant not on small chemical substances or radicals but rather on enzymes involved either in generating free radicals in the cells (e.g., NAD(P)H oxidase or xanthine oxidase) or in protecting the cell against free radicals (superoxide dismutase—SOD, catalase—CAT, glutathione peroxidase—GPX, glutathione reductase—GR, glutathione S-transferases—GSTs, thioredoxin reductases—TRs, heme oxygenase—HO-1/HSP32, biliverdin reductase—biliverdin reductase—BVR) [37]. Such enzymes can be studied in both cell-free and cell systems, and each approach has strengths and shortcomings. For instance, certain natural compounds might have an antioxidant effect on isolated chemicals or free radicals but not necessarily in the cellular environment (where they could be inactivated or outcompeted by various cell components) [37]. On the other hand, it is very likely that certain natural compounds, while not active directly on free radicals or pro-oxidant substances, can upregulate certain antioxidant enzymes (increase their expression) or downregulate one or several enzymes involved in generating free radicals; such compounds are said to be indirect antioxidants [24].

It has been increasingly recognized that the antioxidant activities assessed by chemical-based assays (using either HAT or SET mechanisms) do not correlate well with clinical effects. Therefore, there is a need for better assays, such as those based on cell systems [38]. On the other hand, cell systems are more expensive, time consuming, and complex and, therefore, do not lend themselves to a straightforward interpretation [31]. However, such systems have become sufficiently mature and robust to allow use in high-throughput applications, and it has been opined that they should now replace HAT- and SET-based assays [38]. Due to economic and logistic constraints, though, such a replacement is expected to take place only gradually.

By far, most papers that reported antioxidant effects for essential oils from Pinaceae included a DPPH method, often together with at least one additional assessment method (e.g., [39,40]). Still, in many cases, DPPH was the only antioxidant method employed (e.g., [41,42]). Prima facie, this could create the impression that comparing the antioxidant effects of essential oils prepared from Pinaceae species, as measured through this method, should be relatively straightforward. Unfortunately, comparisons are often impossible to make because of a wide variability in how the technique was performed in positive controls (or lack thereof) and in how results were expressed. For instance, spectrophotometric measurements are usually carried out after maintaining the prepared mixture of sample and free radicals (DPPH) for a particular duration. Although most often this duration was 30 min (e.g., [39,40,43,44,45]), in various published papers, it varied considerably: 10 min [46], 20 min [47,48,49,50,51], 25 min [52], 40 min [53], 50 min [42], 60 min [54,55,56,57,58,59,60,61], or 70 min [62]. Very often, the period after which the spectrophotometric measurements were performed was not mentioned (e.g., [63,64,65]). Sometimes, this lack of detail was supplied through a reference. Still, because it was stated that the method in the reference was applied with minor adaptations, one could not be sure whether the measurement timing changed. In a few cases, the authors reported a dynamic assessment, i.e., performing measurements at multiple time points (e.g., 20, 40, 60, 90, and 120 min [41,66]). This approach is helpful as it provides more information on the behavior of the essential oil in contact with the evaluated free radicals (DPPH). Still, in this specific example, the most widely used time point (30 min) is lacking, preventing comparisons with measurements performed by other laboratories at 30 min.

In most cases, the spectrophotometric measurements for the DPPH assay were performed at room temperature or, as explicitly stated in two cases [67,68], at 25 °C. However, one paper reported incubating the DPPH mixture at 37 °C before performing the spectrophotometric measurements [69]. 

A variety of positive controls were used: ascorbic acid [47,52,58,70,71,72,73,74], alpha-tocopherol [45,52,64,75,76,77], BHA [45,55,74,78,79], BHT [42,44,45,54,58,67,76,78,80,81], beta-caryophyllene [64], caryophyllene oxide [64], quercetin [42], tannic acid [82], gallic acid [60,83], thymol [84], and even *Thymus vulgaris* essential oil [62].

Further differences were found for the endpoint used to report the results of the DPPH test. Whereas in many cases this was based on an IC_50_ value (e.g., [47,54,69]), despite using a synonym name (such as EC50—half maximal effective concentration [41,79], RC50—50% reduction concentration [85], or SC50—50% scavenging concentration [42]), in other cases, different endpoints were used as follows:

- Percentage of DPPH inhibition measured for a single sample prepared in a wide variety of ways (i.e., very different concentrations, e.g., 5 mg of essential oil diluted to 5 mL with ethanol, treated with 250 µL of DPPH in methanol (5.07 × 10^−4^ M) [55], 100 µL of essential oil mixed with 3.9 mL of DPPH solution [86], 50 μL/mL [87], or even without details on the way the sample was processed but referencing a published source without clear statement whether identical amounts were used [53,82,88]);
- Percentage of DPPH inhibition measured on three to five different amounts/concentrations of essential oil with no IC_50_ estimation [72,78,89];
- Equivalents to certain antioxidant substances expressed as mg per gram of essential oil (hydroxytoluene equivalent; ascorbic acid equivalent; Trolox equivalent) [90,91], μM equivalents per gram of essential oil [51], mM equivalents per ml [48] or per liter of essential oil [56], or μg of equivalents per ml of essential oil [92].

When a single concentration was used, due to the high variability in the concentrations used, percentages reported are hardly comparable, except for the samples mentioned in the same paper by the same authors. Often, not only were different substances used as reference agents, but the way of expressing the results (gram or moles, per mL/L or mg/g of essential oil) makes them hardly comparable (except for comparisons reported by the same authors in the same paper and through the same methodology). The estimation of an IC_50_ value is highly recommended as a unique point estimate able to encapsulate more information on the antioxidant activity of an EO. However, it is well known that IC_50_ values can also vary widely, depending on the substrate concentration used and other aspects of the experimental design [93]. All these aspects should be considered in interpreting the results reported for the antioxidant activity of essential oils obtained from various Pinaceae species (or any other taxonomic group).

Similar wide variability in performing and reporting results was also found concerning the ABTS method. For instance, results were reported as IC_50_ (in μg/mL [39], mg/mL [64], or % [94]), TEAC (Trolox equivalent antioxidant capacity) (mEq Trolox/g EO [91], μmol TE/g DW [40,48]), the percentage of scavenging activity at defined concentrations [89], and ascorbic acid equivalent/g [74]. There are also differences in how the method was put into practice. The free radical was produced by reacting an aqueous solution of 7 mM ABTS with 140 mM potassium persulfate (K_2_S_2_O_8_), and the two reagents were incubated for 16 h before use; after diluting to an appropriate absorbance value with ethanol and adding the EO, the resulting mixture was incubated for 5 min before measuring the absorbance [39]. In addition to the 7 mM concentration [40,52,64,70], other authors used various concentrations of the ABTS solution as a starting point for producing the free radicals (or added pure ABTS [74]): 7.4 mM [89,94], 14 mM [95], and 1.4 mM [81]. Most often, they mixed the ABTS solution with 2.4/2.45/2.46/2.5 mM potassium persulfate [40,52,64,71,89,94,96,97], but 140 mM [48] or 4.9 mM [81,95] persulfate solutions were also used. Venditti et al. generated the ABTS radical by adding 0.6 g of manganese oxide to an ABTS solution and leaving the two reagents in contact for 20 min [51]. Various experimenters reported waiting for 12–16 h before use [40,74,89,96], 14–16 h [52], 14 h [71], 16 h [64,81,95,97], or “one day in advance” [48]. Absorbance measurement was carried out after 3 min [52], 5 min [91], 6 min [40,48,64,70,71,81,89,95,96,97], 7 min [71], or 30 min [74,94]. The solution was diluted to an appropriate absorbance level with pure water [70,74,97] or methanol [52,71]. For DPPH, various substances were used as positive controls: ascorbic acid [52,70,71,94,97], Trolox [39,40,48,51,97], alpha-tocopherol [52,64], BHT [81], BHA [74,94], beta-caryophyllene [64], and caryophyllene oxide [64], and in multiple cases, no comparator was employed [89,91,95,96]. Although active controls were mentioned as used, their antioxidant values were sometimes not reported [94], and are the equivalent of no control.

Specific authors only referenced an original method from the literature for FRAP without specifying any adaptations [80], and others used various modifications [40]. In one variant, the FRAP reagent was obtained by mixing a 10 mM TPTZ in 40 mM HCl, 20 mM ferric chloride, and 300 mM acetate buffer (pH 3.6) (1:1:10) [40,48,51,71,77,98]. Others (Oyaizu method) mixed the EO with equal aliquots of potassium ferricyanide (1% solution) and a pH 6.6 phosphate buffer (0.2 M). After incubation for 20 or 25 min at 50 or 55 °C, the reaction was stopped with trichloroacetic acid, centrifuged, and the supernatant was mixed with H2O2 and ferric chloride [52,64,68,70,78,81,97,98,99]. Sometimes, this latter method is described only as “reducing power” and as distinct from FRAP [77] (whereas other authors treat it as FRAP [52,97,98]); as discussed later in this paper, the data available for bark EOs were obtained from *Pinus pumila* (Pall.) Regel [77], which clearly shows that the two methods are not equivalent and should not be treated as the same. The absorbance was measured after 4 min of incubation at 37 °C [100], 10 min of incubation at 37 °C [40,48], 30 min of incubation at 37 °C [51,71,77], or 20 min of incubation at 55 °C [52,78,98]; these differences in sample processing (temperatures and durations) not only could impact the redox reaction but also the stability of various EO constituents. The result reporting was as diverse as other methods. Some papers reported FRAP as antioxidant capacity in Trolox equivalents (μmol TE/g DW [40,51], μmol eq Trolox/mL EO [48], Trolox equivalents/g [77]), and gallic acid equivalents [61], others estimated EC50/ IC_50_ values (μg/mL [52,68,99], mg/mL [81]), and others reported the absorbance measured at 700 nm (“A700 value”) [98] or were sometimes simply unclear as to what was used [61]. Active controls were, as usual, diverse: Trollox [40,48,77], ascorbic acid [52,71,81], alpha-tocopherol [52], chlorogenic acid [98], BHA [68], and BHT [68].

The beta-carotene bleaching test was less widely used than DPPH, ABTS, or FRAP, but we identified at least eight publications in which it was used on Pinaceae EOs [52,60,62,64,67,68,77,101]. In one variant, the beta carotene–linoleic emulsion was prepared using 0.2 mL of the β-carotene solution (1.0 mg/mL with chloroform as the solvent), 20 μL of linoleic acid, 200 mg of Tween 40, and 50 mL of oxygen-enriched water (oxygen flow at 100 mL/min for 30 min) [77]. In another variant, to create the emulsion, 3 [64,101] or 4 [68] mL of the β-carotene solution (0.1 mg/mL in chloroform) was combined with 40 mg of linoleic acid and 400 mg of Tween 40 (or similar recipes [52,62,67]). The solution thus obtained was evaporated most often at 40 °C (in one case at 45 °C [52]) (5–10 min) to eliminate the solvent. Finally, the emulsion was prepared by gradually adding 100 mL of distilled water to the dried residue, accompanied by energetic stirring [52,62,64,68]. The reaction was reported to take place at 50 °C [64,68,77]. In addition to the baseline measurement, the UV absorption was measured at 30 and 60 min [77] and 60 [62,64,101], 100 [52], or 120 min [67,68]. A variety of substances were used as positive controls: ascorbic acid [52,77], alpha-tocopherol [52,64,77], BHA [67,68], BHT [60,67,68], rutin [101], Trollox [62], and Thymus vulgaris EO [62].

A method related to beta-carotene bleaching is based on inhibiting linoleic acid peroxide formation. The EO sample (diluted in ethanol) is mixed with a solution of linoleic acid, ethanol, and a pH 7 sodium phosphate buffer and then incubated for a relatively long period (175 h). Peroxide value is then estimated spectrophotometrically using a method based on a complex formation with ammonium thiocyanate and ferrous chloride. This method was used once to evaluate EOs from Pinaceae [61].

Nitric oxide scavenging was assessed via the Griess reagent method using naphthyl ethylenediamine and sulphanilamide (in an acidic environment) to react with NO (generated by sodium nitroprusside), resulting in a colored azo compound [102]. This method was applied in two papers to evaluate nitric oxide scavenging Pinaceae species’ EOs [69,101].

TBARS (thiobarbituric acid reactive substances) assesses antioxidant properties based on malondialdehyde (MDA) resulting from lipid peroxidation under the attack of free radicals and can react with the thiobarbituric acid to generate a pink-colored dimeric compound. Despite its limitations, it is still used for evaluating the antioxidant effects of EOs [103].

Methods gauging the ability to scavenge hydrogen peroxide can be enzymatic or non-enzymatic [26]. Two such methods applied in assessing EOs from Pinaceae use a spectrophotometric approach based on the direct hydrogen peroxide reaction with the EO in a phosphate buffer (pH 7.4), followed by spectrophotometric measurement at 230 nm [61,88]. Cited several times under the name of “Ruchet et al. (1989)” [88,104,105], it was actually proposed by R. J. Ruch et al. (1989) [106].

Three papers evaluated the hydroxyl radical scavenging using the deoxyribose method, where free radicals generated through a Fenton reaction attack deoxyribose to form malondialdehyde were then measured with the thiobarbituric acid method [84].

The ORAC method, developed in the 1990s, is based on heating an azide compound to generate very active free radicals, which subsequently quench the fluorescence of fluorescein [107]. “ORAC values have been used more as political and marketing tools than as chemical tools” [107], and coupled with its limitations, the USDA decided to withdraw a public ORAC database it had previously developed [108].

The chelating activity was evaluated using a ferrozine method [67,78,91,101] (in one paper, it is inappropriately called “the method of Dinis et al.” [78], when in fact, the publication of Dinis et al. [109] is based on a method published in the 1970s by P. Carter [110]). The results were expressed as % [78], IC_50_ [68,101], or mg Eq EDTA/g EO [91].

The following methods were used only once or in a small number of cases:

- The superoxide radical inhibition based on the autooxidation of pyrogallol [88];
- The superoxide radical scavenging based on nitroblue tetrazolium reduction [70,111];
- The method based on 3-morpholino-sydnonimine and 1-keto-4-methylthiobutyric acid (SIN-1—KMB) [112];
- The Fenton system [113] proposed for use in the antioxidant assessment by Halliwell and Gutteridge (1985) [114];
- The method based on 1-aminocyclopropane-1-carboxylic acid (ACC) fragmentation induced by HOCl (hypochlorous acid) [112];
- The ferric–phenanthroline assay (Phen assay) based on the ability of ferric ions to form a complex with phenanthroline [115];
- The Rancimat method, developed by Hadorn and Zurcher in the 1970s (in a publication in the German language [116]), established itself as one of the most widely utilized accelerated techniques for assessing the oxidative stability of fats and fat-containing foods [117,118];
- A method based on the xanthine/xanthine oxidase system [112];
- A method based on the NADH/diaphorase system [112];
- Two methods based on the impact of the EO on catalase and glutathione reductase [119];
- The phosphomolybdenum method (total antioxidant capacity (TAC) assay) [64];
- The Folin–Ciocâlteu method of quantifying total phenolics [43];
- The 20,70-dichlorofluorescein diacetate probe to estimate the intracellular antioxidant activity in the human keratinocytes HaCaT cell line [73].

The most extensive investigation of the antioxidant effects of multiple EOs from Pinus species used the luminol chemiluminescence assay [120]. Luminescence, the emission of light by excited molecules returning to their ground state, comes in many forms, depending on the energy source, such as photoluminescence (including fluorescence and phosphorescence), pyroluminescence, triboluminescence, cathodoluminescence, crystalloluminescence, and chemoluminescence. The latter is luminescence powered by chemical reactions [121]. Luminol (5-amino-2,3-dihydrophthalazine-1,4-dione) is an organic substance capable of chemiluminescence in the presence of oxidants (such as free radicals), whereby its oxidation results in an excited electronic state, which upon relaxation to the ground state, emits light (maximal emission at 425 nm) [122].

## 3. Comparing Antioxidant Effects of Pinaceae EOs

A review paper should offer a synthesis of the data, including comparisons and ranking of EOs from multiple genera, species, plant parts, or other variables of interest. However, such a comparison is difficult to perform, mainly because of the wide variability observed in how methods are applied (see Section 2), how the EOs are extracted, and their chemical composition.

Comparisons made based on data from the same study are, to some extent, more reliable, as they cancel many experimental errors specific to the analyst, equipment, and other particularities of the laboratory. If EOs from several taxons are compared, for instance, but the collection of the plant material was performed at different times in the year or from different areas (with different pedological and climatic conditions), the differences recorded might still be due to other factors that are specific to the taxons analyzed. Using a positive control should facilitate (to some extent) comparators even in the absence of the same methods and experimental details. However, the use of different endpoints and comparators by different experimenters still precludes extensive comparisons across the available data. The results between different labs are often not negligible for the same comparator using the same endpoint. For ascorbic acid using the DPPH method, for instance, the following IC_50_ values have been reported (we converted mg/mL to μg/mL): 1.75 ± 0.69 μg/mL [70], 2.0 ± 0.13 [85], 3.27 μg/mL [47], 5.0 ± 0.4 μg/mL [101], 6.25 μg/mL [71], 11.5 μg/mL [72], 19.0 ± 1.1 μg/mL [69], 22.61 ± 1.08 [97], 40 ± 110 μg/mL [73], 46.54 ± 3.64 μg/mL [52], 53.24 ± 3.25 [59], 54 μg/mL [74], and 84 ± 63 μg/mL [99]. How do we use such a variety of values spanning almost two orders of magnitude? Employing the average is not the most appropriate because they correspond to different experimental conditions. Still, those conditions are expected to have similarly impacted the values estimated for the EO samples. Therefore, although not perfect, we propose comparing IC_50_ values with the use of the “relative potency” (RP), defined as follows:

$$RP(positive\;control)=\frac{{\mathrm{IC}}_{50}\;of\;sample}{{\mathrm{IC}}_{50}\;of\;positive\;control}$$

This calculation assumes that the IC_50_ of both the positive control and the sample are expressed in the same units, and the RP will be a unitless number. It describes how powerful or weak a sample’s antioxidant effect is with respect to a pre-established positive control. The higher the RP, the lower the antioxidant effects, and the lower the RP, the stronger the antioxidant effects. For instance, if the RP = 0.5, that means that the positive control is twice more active than the sample or that the activity of the sample is about half of that of the positive control; instead, if the RP = 2.0, that means that the positive control is twice more active than the EO or that the EO has only half of the activity of the positive control. Because “strong” or “weak” antioxidant effects are often spoken about without a clear quantitative criterion, we hereby propose an RP scale and the equivalent common terms to describe the strength of antioxidant activity (Table 1).

Because multiple positive controls have been used in different studies, it is helpful to use conversion factors to estimate the RP values in relationships with other comparators. We have analyzed the studies using multiple comparators and computed the ratio between their IC_50_ values determined in the same experimental conditions. Whereas IC_50_ values varied widely (as shown above), the ratio between two pairs of positive controls tended to vary much less, allowing us to estimate the conversion factors (ratios) mentioned in Table 2. Because the most extensive corpus of data was available for DPPH, we estimated these factors for DPPH. Still, with more data, a similar estimation can also be performed for other antioxidant assays.

IC_50_ values and relative potencies against ascorbic acid identified in the studied literature for Pinaceae species on DPPH are listed in Table 3.

In the literature surveyed, we found available antioxidant data from at least one testing method for seventy Pinaceae species: fifty-one for *Pinus* species, eleven for *Abies* species, four for *Picea* species, three for *Cedrus* species, and one for Larix species. A detailed, critical discussion of the findings of various antioxidant results for each species, often with EOs from multiple plant parts (needles, bark, cones, and others) and with multiple testing methods, would take space that is not available for a synthetic review such as this. We intend to conduct a species-by-species discussion in two future papers. Here, we are focused on a synthesis of the main findings.

The DPPH antioxidant effects measured through IC_50_ values for various EOs derived from Pinaceae by species and plant part, where ascorbic acid was used as a reference substance, are shown synthetically in Table 3.

IC_50_ values and relative potencies against alpha-tocopherol, as well as against ascorbic acid (by conversion using the conversion factors in Table 2) based on DPPH results, are listed in Table 4.

IC_50_ values and relative potencies against BHA, as well as against ascorbic acid (by conversion using the conversion factors in Table 2), estimated on DPPH, are listed in Table 5.

IC_50_ values and relative potencies against BHT, as well as against ascorbic acid (by conversion using the conversion factors in Table 2), estimated on DPPH, are listed in Table 6.

Among the EOs with the lowest antioxidant effects in Table 6 is the one obtained from the leaves of *Pinus halepensis* Mill. [81], for which IC_50_ was 73.03 mg/mL (i.e., 73,030 μg/mL), whereas the positive control (BHT) reported in the same study was only 34.23 ± 1.15 μg/mL, resulting in an RP value of over 2000. This value contrasts with the RP value of only 3.42 reported for another EO obtained from the same species (*Pinus halepensis* Mill.) [124]. The latter comes from a study where the IC_50_ reported for BHT was 0.12 mg/mL (120 μg/mL); in other words, the IC_50_ for the positive control in this second study was estimated to have a value about three times larger than in the first study (whereas the IC_50_ for the EO in this second study was 0.41 mg/mL, i.e., about 3.4 times higher than the control). Therefore, the vast difference in RP seems not to originate as much from the experimental conditions as from the differences in the antioxidant effects of the oil tested. Variability in chemical composition could at least partially explain it, as the caryophyllene content was 28.57% in the first EO, whereas it was estimated to be 15.87% in the second, and although β-pinene was under 3% in the first EO, it constituted 13.7% in the second, etc. However, it is likely that factors relating to how the assessment method was implemented were also involved in explaining such discrepancies.

Based on the relative potencies against ascorbic acid (computed directly from the source data or through conversion factors against other comparators), we have pooled the available data on DPPH from multiple sources and ranked them in Table 7.

Only a small number (six) of EOs obtained from the species of Pinaceae have stronger potencies than ascorbic acid. Of the six EOs with stronger antioxidant potencies than ascorbic acid, four are derived from the genus *Pinus*, one from *Abies*, and one from *Cedrus*. Among the four *Pinus* EOs, three were obtained from the wood of a single species (*Pinus pinaster* Aiton) using different newer extraction methods and from a single publication [100]. The other *Pinus* EO was obtained from the needles of *Pinus thunbergii* Parl. [77]. A leaf EO of *Cedrus deodara* (Roxb. ex D.Don) G.Don was about three times more active than ascorbic acid [70]. In contrast, a leaf EO of *Abies numidica* de Lannoy ex Carrière was slightly superior to the reference antioxidant [54]. At the opposite pole, a feeble effect was also reported for a leaf EO derived from *Abies numidica* de Lannoy ex Carrière, which at 800 μg/mL did not manage to cause 50% inhibition of DPPH radicals (unlike the reference substances used by the authors) [45]. “No activity” or very weak activities were also reported for *Pinus pinaster* Aiton (two producers) [73], *Abies balsamea* (L.) Mill. [44], *Larix laricina* (Du Roi) K. Koch [44], *Picea glauca* (Moench) Voss [44], and *Pinus nigra* J. F. Arnold ssp. *dalmatica* (Vis.) [80].

We also provide rankings from different studies carried out with DPPH using various endpoints, which could not be pooled in a single comparison but are still relevant for comparing EOs from at least two distinct taxons. These are listed in Table 8.

Comparisons made among EOs derived from Pinaceae species evaluated for their antioxidant effects through the ABTS method are synthesized in Table 9.

Comparisons made among EOs derived from Pinaceae species evaluated for their antioxidant effects through the FRAP method are synthesized in Table 10.

Although the luminol chemiluminescence assay was employed in a single paper evaluating the antioxidant effects of EOs from Pinaceae (all belonging to genus *Pinus*), this was the most impressive paper in its breadth, reporting on EOs from no less than 46 *Pinus* species (but only EOs obtained from fresh leaves were analyzed) [120]. The IC50 and RP (beta-carotene) of the EOs from the fresh needles of the 46 species are shown in Table 11.

The variation of EO antioxidant effects depending on the part from which they were obtained is shown synthetically in Table 12.

The influence of the extraction method on the antioxidant effects of EO is shown synthetically in Table 13.

## 4. Variability of EO Antioxidant Effects

There is sizeable intra-study variability related to the specimen origin, as evidenced in several studies. Dakhlaoui et al. (2023) [81] analyzed EOs obtained from needles of *Pinus halepensis* collected from eleven sites corresponding to distinct bioclimatic areas in Tunisia. The point estimate of the IC_50_ for the DPPH test (mean of three replicates) varied between 73.03 and 270.86 mg/mL, with a mean (of the eleven-point estimates) of 192.50 and a standard deviation of 57.49 mg/mL; this corresponds to a coefficient of variation (relative standard deviation) of 29.87%. For the ABTS assay, the IC_50_ varied between 197.9 and 577.3 mg/mL, with a mean of 399.2 and a standard deviation of 135.56 mg/mL; this corresponds to a coefficient of variation of 33.96%. Finally, for the reducing powers, the authors reported point estimates (means of three replicas) varying between 1.23 and 3.92, with a mean of 2.00 and a standard deviation of 0.91; this corresponds to a coefficient of variation of 45.57% [81]. Aidi Wannes et al. (2021) also analyzed leaf EOs from *Pinus halepensis* derived from three Tunisian provenances. The IC_50_s on DPPH had a coefficient of variation of 11.27%, whereas the IC_50_s on ABTS had a coefficient of variation of 24.85% [95].

An interesting study evaluated the antioxidant effects of *Pinus mugo* leaf EOs obtained in two distinct years from two altitudes in each of the two years: 1640 and 2039 m. At the lower altitude, the IC_50_ was 3.22 ± 0.4 and 4.26 ± 0.5 mg/mL in the two years. At the higher altitude, the IC_50_ in the two years were 2.65 ± 0.6 and 2.51 ± 0.3. Higher altitudes tended to be associated with lower IC_50_s (higher activities) in both years [46]. The coefficient of variation corresponding to the four values across the two years and two altitudes is 25.16%. Similar findings (similar size and direction) were reported in the same paper for the antioxidant effects evaluated through the thiobarbituric acid reactive substance method (the coefficient of variation, in this case, was 25.25% [46]).

Jaouadi et al. (2011) evaluated the antioxidant effects of EOs obtained from wood tar and sawdust of *Cedrus atlantica* obtained from two distinct Morrocan forests: Itzer (1240 m altitude) and Senoual (2195 m altitude). The differences in IC_50_ (Itzer vs. Senoual) were more pronounced in the case of wood tar EO than for the sawdust EOs: 0.126 ± 0.013 vs. 0.143 ± 0.014 mg/mL for the wood tar and 15.559 ± 0.715 vs. 16.264 ± 0.285 for the sawdust, and in the case of DPPH; 0.832 ± 0.002 vs. 0.410 ± 0.002 (wood tar), and 2.219 ± 0.001 vs. 1.996 ± 0.013 mg/mL (sawdust) for FRAP [99].

Ruas et al. (2022) evaluated (inter alia) the antioxidant effects of three *Pinus pinaster* Aiton leaf EOs derived from three different producers. Whereas for one of them, low antioxidant effects were recorded against DPPH (IC_50_ 55.2 ± 0.9 mg/mL), the other two were entirely inactive in this test. Evaluated with the ORAC method, the three EOs exhibited wide differences in their IC_50_ values: 161.2 ± 24.9, 355.6 ± 30.3, and 565.5 ± 70.4 mM TE/g (a mean of 202 mM TE/g and a coefficient of variation of 56.05%). Finally, concerning the reduction in reactive oxygen species generated by hydrogen peroxide in the HaCaT cell line, the IC_50_ values were 34.3 ± 3.7, 29.5 ± 0.5, and 21.7 ± 1.9% (mean 28.5, coefficient of variation 22.3%) [73]. It may be seen that variation among the three EOs is not consistent among various antioxidant assessment tests. In this case (different producers), and even in the previous ones, the differences observed might be related to different origins of the herbal materials. Still, they might also be more or less due to other variables, such as different preprocessing and extraction methods, the growth stage of the plant at harvest, the timing of the harvest, etc.

The variability of the EO composition and, consequently, of the antioxidant capacity of various EOs is influenced not only by the area of origin (with its pedo-climatic variables) but also by a plant’s age and growth stage. Dejrrad et al. (2017) investigated these aspects in a study examining the antioxidant effects of EOs obtained from six different growth stages (termed by the authors B0–B5). For the first two stages, they reported IC_50_ varying between 228.96 and 236.18 μg/mL, with a mean (of the two values) of 232.57 μg/mL; for the third and fourth stages, IC_50_ varied between 214.93 and 216.87 μg/mL (mean 215.9 μg/mL), and for the last two stages, between 201.28 and 206.79 μg/mL (mean 204.03 μg/mL) [67].

## 5. Correlation among Various Antioxidant Methods

All antioxidant assessment methods attempt, in principle, to measure the same property of an EO, plant extract, or chemical compound: the ability of the tested product to scavenge various free radicals. Because (as discussed in Section 2) a variety of methods are available and have been applied to evaluate the antioxidant effects of EOs, and considering that EOs are complex mixtures of scores of hundreds of compounds with different chemical and physical properties (including different solubilities in different solvents), differences may occur from method to method and from species to species. Often, the same research group applies two or more antioxidant methods, and in some papers, the authors compared the degree of correlation between the results obtained with those methods. When only one or two EOs have been analyzed for their antioxidant effects, formally assessing the correlation among results is impossible. However, using the classical correlation methods (Pearson, Spearman) becomes possible when the number of samples is three or higher. We, therefore, compared the results among various antioxidant method results available for Pinaceae using both the Pearson and Spearman correlation methods. We report the results for the Pearson correlation, and only when the Spearman correlation coefficient is much higher than the Pearson coefficient do we provide the latter (as this indicates a monotonic instead of a linear relationship). We limited the comparison to intra-study results (i.e., results by the same group of authors for the same EOs they have analyzed). The results are shown in Table 14 (source data and plots visually showing the correlation are available in the Supplementary Electronic Materials, Tables S1–S43 and Figures S1–S43).

As may be seen, a wide variability of degrees of correlation was reported. Some papers reported a very high correlation (r > 0.95) between two methods, for instance, between DPPH and ABTS [39,124], but for the same two methods, other papers had lower results (0.84, 0.66 [40,48]) or even no correlation at all (r = −0.10 [91]). These findings should not necessarily be surprising because different assessment methods have different specificities and sensitivities, the antioxidant components of EOs may be very diverse, and the results (usually determined spectrophotometrically) may be influenced by the sample matrix that constitutes the EO. Mechanistic differences among different methods may also contribute to different results, and this is very evident when comparing the results of radical scavenging methods with methods evaluating (ferrous iron) chelation abilities of EOs (correlations tend to be lower or even absent). Sometimes, for the same group of authors, the results of the two methods were very well correlated (e.g., linoleic acid system vs. FRAP). In contrast, there was almost no correlation for other systems in the same paper (e.g., linoleic acid system vs. hydrogen peroxide scavenging) [61].

## 6. Chemical Composition of EOs and Correlation with Antioxidant Effects

The wide variability observed in the antioxidant effects seems likely to reflect the wide variability in chemical composition. Although most EOs from Pinaceae tend to include a number of shared compounds (e.g., α-pinene, β-pinene, limonene, β-caryophyllene, germacrene D, myrcene, β-phellandrene, etc.), there are vast quantitative differences from species to species and often intra-species. This intra-species variability (leaving aside differences from organ to organ) often reflects genetic variability, as different chemotypes often correspond to distinct genotypes [128]. α-pinene, for instance, in several samples of leaf EO from *Pinus pinaster* Aiton, varied from 13.53% [71] to 44.6% [73], whereas β-pinene varied from 9.81% [71] to 28.0% [73], but they were common to all EOs derived from the leaves of this species. Rimuen (a natural diterpene) was reported in a proportion of 9.13% in one sample [71] but not in others because, in the latter, the authors limited the reporting to compounds present in a proportion ≥ 5% (therefore, we do not know if the other samples contain it at all or not) [73]. In the fresh leaf EO analyzed by Ioannou et al. (2014), a very different composition was found, dominated by isoabienol (19.1%), sclarene (18.0%), and germacrene D (11.2%); α-pinene was only found in a 5.2% level, whereas β-pinene was only reported in a small proportion of 0.6% [126].

When examining the composition of essential oils (EOs) in leaves across various genera of the Pinaceae family with available data, there appears to be minimal distinct separation between the genera based on the types of components present. Additionally, due to significant quantitative differences, there is also a lack of clear boundaries between genera based on the amounts of these components. For instance, among the leaf EOs from four *Abies* species for which antioxidant data were available, three of them (*Abies pindrow* (Royle ex. D.Don) Royle [47], *Abies alba* Mill. [85], *Abies balsamea* (L.) Mill. [44]) contained limonene in amounts similar to at least one Pinus species (e.g., 38.9% in *Abies pindrow* [47] vs. 31.7% in *Pinus pinea* [126]). In the leaf EO obtained from *A. numidica*, Benouchenne et al. (2022) did not find limonene as an ingredient, although previous investigations indicated its presence in amounts of 12.7% or 19.7% [45]. There are also species of *Pinus* in whose leaf EOs limonene was not detected (e.g., *P. roxburghii*, *P. densiflora*, *P. massoniana*), as there are species in whose leaf EOs the limonene level is close to 12.7% (*P. nigra* ssp. *salzsmanii*, 12.5% [126]) or close to 19.7% (*P. densiflora*, 20.58% [126]). Similar examples can easily be found for multiple analyses of EOs derived from the same species and plant part but with different origins. In a paper focused on analyzing the variability of EOs from *P. pinea* (twenty-six samples from the literature) and *P. pinaster* (thirty samples from the literature), it was reported that the variability of the six main chemical compounds (limonene, α-pinene, β-pinene, *trans*-β-caryophyllene, germacrene D, β-myrcene) was equal to 90% or higher. The ranges of relative amounts for each of the six compounds were 1–75% (limonene), 1–42% (β-pinene), 1–37% (α-pinene), 1–17% (*trans*-β-caryophyllene), 1–10% (germacrene D), and 1–11% (β-myrcene), respectively [129].

In addition to the variability in chemical composition among different samples from the same species and product, attributable to genetic or epigenetic/environmental factors, the extraction methods contribute to this variability. Several studies have provided direct evidence in this sense, as shown in Table 13. The pretreatment of the herbal material by a variable number of underwater shockwaves (which create a sudden high pressure that breaks cell walls), followed by conventional steam distillation, results in different extraction yields of EOs and different antioxidant activities of those EOs. Untreated dried samples give yields of about 2.4 g/kg (on a dry basis), whereas untreated fresh samples give about twice that yield (5.1 g/kg). Using shockwave pretreatment increases dramatically the yield, from 16.7 g/kg with two additional shockwave cycles up to 32.7 g/kg with ten shockwave cycles [43]. As shown in Table 13, though, the highest yield does not necessarily imply the highest antioxidant effect (which was maximal for the samples prepared with seven shockwave cycles). The impact of the method of extraction in this case is dramatic. For instance, α-pinene has a content of about 7.73% in the EO obtained by steam distillation (no pretreatment) from fresh leaves and only 0.66% obtained by the same conventional method from dried leaves. However, in the samples obtained using various cycles of shockwave treatments, the α-pinene proportion varied between 13.25 and 16.15%, i.e., about twice the amount obtained conventionally from fresh leaves. Similar examples could be provided for most other compounds analyzed in this study [43].

Meullemiestre et al. (2013) evaluated five methods of EO extraction of *Pinus pinaster* wood: hydrodistillation (HD), turbo-hydrodistillation (THD), ultrasound-assisted extraction (UAE-HD), solvent-free microwave (SFME), and microwave hydrodiffusion and gravity (MHG). As shown in Table 13, the extraction method influenced the antioxidant performance of the obtained EOs, with SFME and MHG having the highest activity. Each method was associated with a different chemical profile. For instance, α-pinene content was about 0.4% in the sample obtained by HD, only about half (0.2%) in the MHG sample, 0.6% in the SFME sample, 1.2% in the UAE-HD sample, and 2.6% in the THD sample. The proportion of monoterpene hydrocarbons was the lowest in MHG samples, slightly higher in HD and SFME samples, considerably higher in THD EOs, and the highest in UAE-HD EOs. Vast differences have also been observed with respect to the extraction of oxygenated monoterpenes, sesquiterpene hydrocarbons, or oxygenated sesquiterpenes [100]. Such differences induced by the extraction method could partially explain why EOs obtained from the same species and part differ in chemical composition and antioxidant performance. To this component of variability, one must add the genetic and environmental factors that influence the intra-species variability of EOs.

Correlation can be easily measured among two variables, but in the case of complex mixtures, such as EOs, using classical correlation tools to evaluate antioxidant effects becomes rather useless, and new methodologies have to be developed. One approach to solve this issue could be using multiple regression or machine learning algorithms, but such methods need appropriate sample sizes, and the larger, the better. However, for most EOs (from Pinaceae but also for most other plant taxa), we often have less than a handful of data sets, and such an attempt becomes hampered by the so-called “curse of the dimensionality”, generally operating in the case of metabolomics [130]. More chances could have an approach based on modeling the effects of mixtures (e.g., antioxidant effects) based on measuring the individual impact of each component of the mix and weighting by its proportion. This approach is not yet possible because for many pure ingredients of Eos, no antioxidant effects are reported individually in the available scientific literature. However, for a few compounds, such data are available. Yang et al. (2010) [131], for instance, evaluated antioxidant effects for six Eos (none from Pinaceae), as well as for four ingredients of such Eos: 1,8-cineole, limonene, α-pinene, and β-pinene. They found that on DPPH, the most potent effect was that of limonene, whereas α-pinene and 1,8-cineole had a negligible effect. Limonene and β-pinene still exhibited higher antioxidant effects on ABTS than α-pinene and 1,8-cineole, but the differences among the four compounds were considerably less pronounced, and all four compounds were much inferior in effects to Trolox [131].

The variability of the different ingredients in Pinaceae EOs is impressive, and probably the most extensive and most remarkable study to date is the one published by Ioannou et al. (2014) [120] on EOs obtained from fresh leaves of 46 Pinus species. The authors found wide qualitative and quantitative variations in the composition of those EOs. Examining the chemical composition and antioxidant effects estimated by the authors serves well to our understanding of the difficulties in correlating the antioxidant effects of EOs with their chemical composition. For several species, for instance, the most abundant ingredient was germacrene D (*Pinus canariensis* C. Sm., *P. muricata* D.Don, *P. nigra* ssp. *nigra*, *P. nigra* ssp. *salzmannii* (Dunal) Franco, *P. cembra* L., *P. thunbergii* Parl., *P. strobiformis* Engelm., *P. koraiensis* Siebold & Zucc., *P. elliottii* Engelm., *P. patula* Schiede ex. Schltdl. & Cham.); however, even though all these taxa had germacrene D as the primary ingredient, their composition varied considerably, and the germacrene D content ranged between 18.5% (*P. nigra* ssp. *salzmannii*) and 44.0% (*P. canariensis*). The highest antioxidant effect in this study (evaluated through the luminol chemiluminescence assay) was recorded for the EO with the highest content in germacrene D (*P. canariensis*, 44.0%). However, it is unclear to what extent germacrene D contributed to this effect because the fourth most active EO (from *Pinus sylvestris* var. *sylvestris*) only contained 5.1% germacrene D [120].

The major components of the leaf EO of *P. canariensis* were germacrene D (44.0%), α-pinene (14.6%), β-caryophyllene (8.7%), limonene (7.9%), myrcene (6.4%), and δ-cadinene (4.1%). Which would play the major role in the antioxidant effect recorded for this EO? In the literature, it has been shown that germacrene D, β-caryophyllene, and β-pinene all have relatively similar antioxidant effects and are rather moderate in potency (IC_50_ on DPPH 80.0, 73.2, and 78.1 μg/mL) [132]. Therefore, it is unlikely that any of the three is the leading actor in the relatively potent antioxidant effect observed for this EO. Limonene was the most abundant ingredient in the fresh leaf EO derived from *P. pinea* L. (31.7%) and *P. heldreichii* Christ. (23.7%), and despite its relatively high antioxidant activity on DPPH compared to that of α-pinene and β-pinene [131], the two EOs rich in limonene had modest antioxidant effects in the luminol chemiluminescence assay, and EOs with much lower levels of limonene had higher antioxidant activities [120]. In the leaf EO of *P. canariensis,* the limonene content was limited to 7.9%; therefore, it seems unlikely that it played a major role in the strong antioxidant recorded for this EO. Myrcene was also reported to have a modest antioxidant effect on DPPH (inferior to that of α-pinene) and to be inactive on ABTS free radicals [133]. In a rat brain homogenate lipid peroxidation study, it was claimed that δ-cadinene was about ten times more active than germacrene D (IC_50_ 3.2 μM for that δ-cadinene vs. 34 μM for germacrene D) [134]. Therefore, one could be tempted to attribute this important antioxidant effect of the leaf EO of *P. canariensis* to δ-cadinene, as none of the other significant compounds seem to be able to explain it. However, there are EOs in the same data set with a higher amount of δ-cadinene but with a much lower antioxidant effect (e.g., the leaf EO from *Pinus sylvestris* L. contains 7.2% δ-cadinene but has an RP value of 21.13, the leaf EO from *P. contorta* var. *latifolia* contains 8.4% δ-cadinene and has an RP value of 41.61, whereas that from *P. canariensis* with 4.1% δ-cadinene has an RP value of 4.35). It seems that the relatively strong antioxidant effect of this leaf EO (of *P. canariensis*) should actually be attributed to one or several minor compounds with strong antioxidant activities (such a conclusion has also been formulated for the pharmacological effects of some EOs [135]). Such an analysis could be made for each of the EOs reviewed, but it is evident that a more rigorous quantitative approach is far preferable. In a future paper, we intend to explore or develop a scientific tool intended to gauge quantitative relationships between the chemical composition of EOs and their antioxidant effects.

## 7. Pharmacological Interest of Pinaceae EOs

The EOs obtained from Pinaceae species have been explored to a variable degree for certain potential pharmacological properties. Probably the most widely evaluated activity is the antimicrobial one. For instance, EOs from various parts of *Pinus halepensis* cones [136,137], leaves [97,138,139,140,141,142], aerial parts [143], bark [137,141], seeds [137], and gum [144] have been repeatedly evaluated for their antibacterial, antifungal, or (less often) antiprotozoal, anthelmintic, and antiviral activities. EOs from various parts of many other Pinaceae species have also been evaluated for their antimicrobial effects, for instance, *P. banksiana* [44], *P. brutia* [145,146], *P. cembra* [39,79], *P. densiflora* [76,94,147,148,149,150], *P. eldarica* [127,151], *P. halepensis* [83,95,136,152,153,154], *P. heldreichii* [92], *Pinus kesiya* Royle ex Gordon (syn. *P. insularis*) [155], *P. koraiensis* [148,156,157,158], *P. merkusii* Jungh. & de Vriese [155], *P. monticola* [159], *P. mugo* [39,92,160], *P. nigra* [74,92,160,161,162], *P. nigra* subsp. *dalmatica* [163], *P. nigra* subsp. *laricio* [146], *P. nigra* subsp. *nigra* [74], *P. nigra* subsp. *pallasiana* [74], *P. nigra* var. *banatica* [74], *P. oocarpa* Schiede ex Schltdl. [155], *P. parviflora* [84], *P. peuce* [92,160], *P. pinaster* [83,139,164], *P. pinea* L. [51,101,139,146,152,165], *P. ponderosa* [166], *P. resinosa* [166], *P. rigida* [147], *P. roxburghii* [82,101,167,168,169,170,171], *P. sibirica* [66,172], *P. strobus* [159,166], *P. succinifera* [173], *P. sylvestris* [92,160,172,174,175,176,177,178], *P. wallichiana* [159], *P. thunbergia* [76,147], *Abies alba* Mill. [39,179,180,181,182], *A. balsamea* [44,183], *A. cephalonica* Loudon [184], *A. cilicica* subsp. *cilicica* [185], *A. cilicica* subsp. *isaurica* [185], *A. concolor* (Gordon) Lindl. ex. Hildebr. [179], *A. firma* Siebold & Zucc. [179], *A. holophylla* [94,186,187], *A. koreana* [94,186,188,189], *A. nephrolepis* [94], *A. nordmanniana* subsp. *equi-trojani* [185], *Abies nordmanniana* subsp. *nordmanniana* [185], *A. numidica* [54,190,191], *A. pinsapo* Boiss. [179], *A. sibirica* [66,172,181,192,193], *Picea abies* [39,94,161,172,194,195,196], *P. excelsa* [197], *P. glauca* [44], *Picea koraiensis* [94], *P. mariana* [44], *P. orientalis* (L.) Peterm. [198], *P. smithiana* (Wall.) Boiss. [199], *Larix decidua* (L.) Mill. [161,195,200], *Larix kaempferi* (Lamb.) Carrière [201], *Larix laricina* (Du Roi) K.Koch [44], *Cedrus atlantica* [90,202,203,204,205,206,207], *Cedrus brevifolia* (Hook.f.) Elwes & A.Henry [208], *Cedrus deodara* [70,209,210,211], and *Cedrus libani* [51]. The activity against phytopathogens (potential use as biopesticides) was also investigated, although to a smaller extent, e.g., for EOs derived from *P. mugo* [212]. Reviewing exhaustively all such data would exceed the available editorial space for this paper. Mixtures of EOs from several *Pinus* EOs were reported to be more active (acting synergically) against microbial species commonly involved in otitis infections [213].

The activity of multiple *Pinus* aerial part EOs has also been evaluated on the larvae of *Aedes albopictus*; those derived from *P. halepensis*, *P. brutia*, and *Pinus brutia* var. *pityusa* (Steven) Silba (syn. *P. stankewiczii*) were considered highly active, even at doses of 0.2 μL cm^−2^ [214]. The leaf EO of *Pinus kesiya* had relatively modest larvicidal activity on *Anopheles*, *Aedes*, and *Culex* vectors, with IC_50_ values varying between 52 and 62 µg/mL [215]. A *C. deodara* EO obtained from the aerial parts [216] and a *C. libani* EO obtained from seeds [217] had larvicidal activity against *Culex pipiens*. A leaf EO of *P. nigra* subsp. *mauritanica* had relatively strong insecticidal activity against coleoptera *Callosobruchus maculatus* (Cowpea Weevil) [64]. A commercial EO derived from *A. balsamea* (part not specified) enhanced the knockdown effect (loss of flight and upright orientation) of various insecticides on insects, although it had a negligible effect on 24 h mortality; the effect was minor in the case of organophosphates and fipronil [218]. The EOs of wood, cones, and leaves of *C. libani* exhibited modest antiviral effects against HSV-1 in vitro, with IC_50_ values of 0.44–0.66 mg/mL, and the selectivity was relatively low [219].

A fresh needle EO of *Pinus nigra* subsp. *pallasiana* was reported to exert scolicidal activity against *Echinococcus granulosus*, the causative agent of hydatid cysts [220]. EOs prepared from aerial parts of *P. pinea* exhibited anthelmintic activity on the model earthworm *Allolobophora caliginosa*, whereas the EOs similarly obtained from *P. halepensis* were less active [152]. A *Cedrus atlantica* leaf EO had relatively strong molluscicidal activity against *Bulinus truncates*, while leaf EOs obtained from *P. halepensis*, *P. brutia*, *P. pinaster,* and *P. pinea* were also active, but their activity was inferior to that of *C. atlantica* [221].

Based on non-clinical experiments, a number of Pinaceae EOs have been claimed to have antiinflammatory effects. Thus, an EO of *Abies koreana* E.H. Wilson needles exhibited antiinflammatory effects on RAW 264.7 cells, inhibiting the LPS-induced secretion of several inflammatory cytokines (TNF-α, IL-1β, IL-6), as well as the secretion of NO and PGE2. Corroborating these results with those observed for the same EO against *Propionibacterium acnes* and *Staphylococcus epidermidis*, the authors suggested its potential use in skin health care [222]. There is speculation supported by non-clinical experimental data that the whitening and anti-wrinkle activities of this EO are related to its content in borneol and borneol acetate, which inhibit tyrosinase and matrix metalloproteinase 1 while favoring collagen I synthesis [223,224]. Similar antiinflammatory effects were reported for EOs prepared from twigs of *Pinus peuce*, *Pinus mugo*, and *Pinus heldreichii*, as well as those from *P. mugo* cones. In all three species examined, the antiinflammatory effects were found to be more pronounced when using essential oils extracted from twigs compared to those from leaves or cones. It is hypothesized that α-pinene, imonene, and δ-3-carene would be the main active ingredients [135]. Both cone and leaf EOs of *P. pinaster* exhibited no significant antiinflammatory activity in a carrageenan paw edema murine model. In contrast, the cone EO inhibited vascular permeability induced with acetic acid in a mouse model at a level inferior to that of indomethacin, though (30.3% inhibition for the EO vs. 42.8% for indomethacin) [71]. The EO obtained from leaves of *P. brutia* was also claimed to have antiinflammatory effects, but these results were derived from a human red blood cell membrane stabilization and an albumin denaturation test [225]. A bark EO of *P. roxburghii* manifested no interaction with canabinnoid receptor CB1 and minimal interactions (2.9–22%) with CB2. The bark EO of *P. roxburghii* antagonized the increase in myeloperoxidase, L-6, and TNF-α levels induced by bleomycin in the lung tissues of treated mice [226]. The inhibitory effects of a wood EO of *Cedrus atlantica* on 5-lipoxygenase and tyrosinase were inferior to those of quercetin, but the latter is not a clinical-use reference product. Therefore, these activities seem rather modest [52]. Wood EOs of both *Cedrus deodara* [227] and *C. atlantica* [228] showed antiinflammatory activity in carrageenan-based models in rats and, in the case of *C. atlantica*, also in the formalin test. The *C. deodara* wood EO apparently inhibits enzymes involved in drug metabolism; it is devoid of any analgesic or sedative properties [227]. An EO obtained from the “dried rhizome” of *C. deodara* exhibited antiinflammatory effects in an auricle swelling murine model by inhibiting the expression of NF-kB, TNFα, and COX-2 [229]. A wood EO of *C. deodara* administered orally demonstrated antiinflammatory and analgesic activities in rat models (carrageenan-induced paw edema, acetic acid writhing test, and hot plate test—at 50 and 100 mg/kg body weight) [230].

On RBL-2H3 cells (a histamine-releasing cell line often used experimentally to assess potential antiinflammatory and anti-allergic effects), a wood EO of *P. densiflora* inhibited the release of IL-4 and IL-13, but the effect was inferior to that of dexamethasone. Additional experimental evidence indicated longifolene as the responsible compound [231]. Similar effects were reported for a wood EO of *P. koraiensis.* Still, in addition to longifolene, the authors found that (+)-α-pinene was even more potent (close in effect to dexamethasone), and smaller effects were also found for 3-carene, limonene, and (+)-α-terpineol; the effect of β-pinene was negligible [232]. On the same RBL-2H3 cells, among three Pinaceae wood EOs, one of *P. densiflora* had the highest inhibitory effect on IL-4 and IL-13. In contrast, the wood EO of *P. koraiensis* had a lower effect, and the wood EO of *Larix kaempferi* had the lowest effect [233]. A leaf EO of *A. holophylla* reduced asthma symptoms in a mouse model and inhibited the IL-17 signaling pathway, as well as the activity of NF-kB [234]. The same mechanism was shown to explain the benefits of the same EO in a murine model of allergic rhinitis [235].

In an imiquimod-induced psoriasis model in mice, an EO prepared with the aerial parts of *P. canariensis*, topically applied, was claimed to attenuate psoriasis symptoms in a manner similar to that of mometasone and to decrease the IL-23 and IL-17A serum levels [236].

A needle EO of *P. eldarica* exhibited antinociceptive effects in non-clinical models of inflammatory pain (acetic acid writhing test, formalin test), whereas using the carrageenan and croton oil tests, the authors confirmed the antiinflammatory effects of the EO [237]. A *Cedrus atlantica* EO (part not specified, most likely wood) exerted analgesic effects in a murine model, apparently by stimulating descending pain modulation pathways, involving opioidergic, serotonergic, noradrenergic (α2-adrenergic), and dopaminergic (D1 and D2) receptors. This mechanism mitigates postoperative pain [238]. The same group later suggested, based on further research, that this effect likely results from an interaction with the endocannabinoid receptors (CB_1_R and CB_2_R) [239]. A different research group also reported analgesic effects for a wood EO of *C. atlantica* using two classical non-clinical tests (hot plate and acetic acid-induced writhing) at a dose of 50 mg/kg [228]. The bark EO of *P. roxburghii* manifested no interaction with canabinnoid receptor CB1 and had minimal interactions (2.9–22%) with CB2 [226].

Administered by the inhalation route, a leaf EO of *Abies sachalinensis* showed anxiolytic effects in a mouse model validated on diazepam [240]. A leaf EO of *A. sibirica* was tested on nine male students (mean age 22 years). It was found to diminish arousal levels after visual display terminal work, suggesting its potential for mitigating mental health concerns linked to such work environments [241]. A leaf EO of *Picea mariana* exhibited hypnotic effects in a mouse model, apparently by increasing the expression of GABAARα1 and 5-HT1A protein levels [242].

Results of non-clinical experiments indicate the potential of *Pinus koraiensis* EOs to reduce blood cholesterol levels. This effect might be linked to the upregulation of LDL receptors (at the mRNA level) and the inhibition of an enzyme involved in cholesterol esterification (acyl-coenzyme A: cholesterol acyltransferase—hACAT1 and 2) [243]. The same research group reported that the EO induces the suppression of lipid accumulation in differentiated 3T3-L1 adipocytes, as well as a decrease in the expression of PPAR*γ*, CEBP*α* (CCAAT enhancer-binding protein alpha, a transcriptor regulator involved among others in adipogenesis [244]), FABP (fatty acid-binding protein 4, with a recognized role in the development of insulin resistance and atherosclerosis [245]), and GPDH (a key enzyme involved in triglyceride synthesis [246]); they also confirmed an anti-obesity effect of the EO in a rat model [247]. The same EO was reported to exert potential antidiabetic effects in a murine model, at least partially through quenching reactive oxygen species and inhibiting endothelial NO synthase and VEGF [248]. Several Pinaceae EOs were reported to have potential antidiabetic effects through an inhibitory action on α-amylase (e.g., the wood EO of *Cedrus libani* [249], the cone EO of *C. deodara* [250], the twig EO of *P. sylvestris* [176], or the leaf EO of *Pinus nigra* subsp. *pallasiana* [251]) or α-glucosidase (*P. wallichiana*, *P. patula*, *P. roxburghii*, *P. gerardiana* [252], *A. numidica*—the latter about five times more active than acarbose [45]). The EOs extracted from the twigs and leaves of *P. densiflora* demonstrate inhibition of elastase and hyaluronidase enzymes, suggesting potential anti-aging properties associated with these oils [253].

An EO of *P. halepensis* exhibited hepatoprotective and reno-protective effects against aspirin-induced damage in an experimental rat model [254]. A *Cedrus deodara* wood EO showed gastro-protective (reduction in gastric juice volume and acidity) and antiulcer effects in a rat model [255]. Leaf EOs from several Pinus species (*P. halepensis*, *P. roxburghii*, and *P. canariensis*, and particularly *P. pinea*) exhibited in vitro antimicrobial effects against *H. pylori*, and in silico, a few of several of their ingredients were claimed to inhibit *H. pylori* urease and shikimate kinase, thus suggesting a potential antiulcer effect for these EOs [256]. A needle EO of *P. eldarica* showed in vitro cytoprotective and genoprotective effects against cis-platin on HUVEC cells [123].

Cone EOs obtained from *P. pinea* L. and *P. halepensis* Mill. exhibited wound healing activity in non-clinical models, whereas those of *P. brutia*, *P. nigra*, and *P. sylvestris* were reported to be devoid of such activities in the same experimental setting [257]. An EO obtained from *P. pinaster* cones exhibited wound healing activity superior to that of leaf or wood extracts or EOs in a wound model with linear incision. However, the tensile strength recorded for the cone EOs was only about half that observed for the positive control (Madecassol ^®^) [71]. In a study that evaluated the wound healing activity of several Pinaceae cone EOs (*Abies cilicica* subsp. *cilicica*, *A. nordmanniana* subsp. *bornmulleriana*, *A. nordmanniana* subsp. *equi-trojani*, and *Abies nordmanniana* subsp. *nordmanniana*, *Cedrus libani*, *Picea orientalis*) those from *Cedrus libani* and *Abies cilicica* subsp. *cilicic* had the strongest wound healing activity in both models used for evaluation (unlike the remainder of EOs, whose activity was negligible) [258]. Wound healing activity properties were also claimed for the fresh leaf EO of *P. sibirica* [259].

Among EOs from five *Pinus* species (*P. brutia*, *P. halepensis*, *P. nigra*, *P. pinea*, and *P. sylvestris*), a twig EO and a leaf ethanol extract from *P. halepensis* had the highest (in vitro) activity against acetylcholinesterase and butyrylcholinesterase (83.91 ± 3.95% and 82.47 ± 5.57% inhibition at 200 μg mL^−1^) [98]. In an independent study, the EO from juvenile leaves demonstrated the highest inhibitory activity against acetylcholinesterase (1.37 mg eq dopenzil/g EO) [91]. Among leaf EOs from three other *Pinus* taxa (*P. nigra* subsp. *nigra*, *P. nigra* var. *calabrica*, and *P. heldreichii* subsp. *leucodermis*), the one from *P. heldreichii* subsp. *leucodermis* was the most active against acetylcholinesterase and butyrylcholinesterase (IC_50_ values of 51.1 and 80.6 μg/mL, respectively) [260]. The fresh needle EO of *P. nigra* subsp. *dalmatica* inhibited acetylcholinesterase stronger than physostigmine, an effect attributed to a good extent to α-pinene and β-pinene [80]. The leaf EO of *A. numidica* had only a weak inhibitory effect on acetylcholinesterase [45].

Multiple EOs were reported to have in vitro antiproliferative activities. A cone EO obtained from *P. roxburghii* was reported to be cytotoxic against the MCF-7 cancer cell line [261], and a leaf EO from the same species was modestly active against the A-549 lung cell line (IC_50_ 161.30 μg/mL) and T98G glioblastoma cell line (IC_50_ 154.30 μg/mL) [169]. A needle EO of *P. roxburghii* was also active in vitro against several cancer cell lines, upregulating pro-apoptotic genes and inhibiting several proteins associated with cell survival, proliferation, and metastasis [262]. However, in one study that compared the cytotoxic effects of a cone EO from *P. roxburghii* with those of mitomycin C, the reported effects were very slightly inferior to those reported for mitomycin C, but when one compares the concentrations used, for the EO it was 100 μg/mL, whereas for mitomycin C, it was 10^−5^ μg/mL, indicating that the EO is about seven order of magnitude inferior to mitomycin C [263]. A leaf EO of *P. sylvestris* var. *mongolica* was also reported to be active against MCF-7 cells but at relatively high concentrations (100 μg/mL) [49].

EOs derived from different *Cedrus* species were cytotoxic in vitro against a variety of cancer cell lines [51,204]. The cytotoxicity of the wood EO of *C. atlantica* on MCF-7 cells was rather modest (IC_50_ 143.13 ± 14.6 µg/mL) [203]. A bark EO of *C. deodara* was claimed to induce apoptosis in colon cancer cells (HCT-116 and SW-620) by interfering with the NFΚB signaling pathway [264]. Leaf EOs of *Abies balsamea* (L.) Mill. [265], as well as those of *A. alba* and *A. koreana* seeds [56], exhibited limited in vitro antiproliferative activities but were rather weak and had low selectivity. EOs from *A. cephalonica* and those from *A. concolor* are also devoid of selectivity in their cytotoxicity, and those from cones tend to be more cytotoxic on normal fibroblasts than those from seeds. Similar effects have also been reported for EOs from cones of *Picea pungens*, *Picea orientalis*, and *A. concolor* [266]. A leaf EO of *P. smithiana* was also reported to demonstrate antiproliferative effects on tumor cell lines in vitro [267]. Compared to a leaf extract, a *P. sylvestris* needle essential oil showed superior cytotoxicity against estrogen receptor-positive and negative breast cancer cell lines [268]. EOs obtained from leaves of *Pinus peuce*, *Pinus mugo*, and *Pinus heldreichii* (and for the latter, also from twigs) exhibited cytotoxic effects in vitro on three malignant cell lines (HeLa, CaCo-2, and MCF-7). It is speculated that germacrene D, β-pinene, and possibly more minor compounds could be responsible for these effects [135]. An EO of *P. mugo* (plant part not stated) was reported to exert its antiproliferative and apoptosis-inducing effects on prostate cells by inhibiting the STAT3 pathway [269]. An EO prepared from leaves of *P. koraiensis* inhibited the cell proliferation and migration of HCT116 colorectal cancer cells, apparently through the suppression of the PAK1 signaling pathway [270]. An EO prepared from pinecones of *P. koraiensis* was active in vitro against gastric cancer cells, apparently through the inhibition of the HIPPO/YAP signaling pathway [271,272]. A leaf EO of *P. eldarica* was more active against HeLa and MCF-7 cells than two different hydro-alcoholic extracts from the same species [273]. A needle EO of *Pinus morrisonicola* Hayata showed a somewhat selective inhibitory effect on several malignant cell lines (A549—human lung cancer, HepG2—hepatoma, MCF-7—breast cancer, PC3—prostate cancer, and HT-29—colon cancer) compared with normal human fibroblasts (HFF cells) through apoptosis, as indicated by the upregulation of several pro-apoptotic genes [274]. A fresh needle EO from *P. wallichiana* (with β-pinene—46.8% and α-pinene—25.2% as the key components) exerted antiproliferative effects on several cancer cell lines: THP-1 (leukemia), A-549 (lung cancer), HEP-2 (liver cancer), IGR-OV-1 (ovarian cancer), and PC-3 (prostate cancer); IC_50_ values for all lines varied between 5.6 and 9.9 μg/mL [72]. A leaf EO of *P. densiflora* had antiproliferative effects on YD-8 human oral squamous cell carcinoma cells, reducing their survival and activating apoptosis [275].

Despite the authors‘ claims, a leaf EO of *Abies pindrow* had modest antiproliferative effects in vitro on several cancer cell lines [47]. EOs obtained from the wood of three *Cedrus* species (*C. libani*, *C. atlantica*, *C. deodara*) and a seed EO from *C. libani* also show potential in fighting cancer. They induce the differentiation of red blood cells in in vitro settings and inhibit the proliferation of several cell lines of chronic myelogenous leukemia and lymphoblastic leukemia, including the multidrug-resistant CEM/ADR5000 line [276,277,278].

In a study that compared the IC_50_ of multiple leaf EOs on A 549 cell lines, the following values were reported for those obtained from Pinaceae (in %, *v*/*v*): 0.179 (*P. densiflora* f. *multicaulis* Uyeki), 0.098 (*Abies nephrolepis*), 0.087 (*Picea abis*), 0.063 (*A. koreana*), 0.027 (*Picea koraiensis*), 0.021 (*Pinus densiflora*), and 0.011 (*Abies holophylla*). On normal human fibroblasts (Detroit 551 cell line), the following IC_50_ values were reported for Pinaceae leaf EOs: 0.311 (*P. densiflora* f. *multicaulis* Uyeki), 0.185 (*Abies nephrolepis*), 0.010 (*Picea abis*), 0.176 (*A. koreana*), 0.019 (*Picea koraiensis*), 0.341(*Pinus densiflora*), and 0.116 (*Abies holophylla*) [279]. It may be seen that most EOs manifested a certain selectivity against cancer cells, but EOs from *Picea abies* and *Picea koraiensis* were more toxic on normal cells. The authors suggested the cell death mechanism involved for all Pinaceae leaf EOs evaluated in this study, based on the variation of cyclins A-E expression, to be cell cycle arrest [279].

A leaf EO of *C. deodara* exerted low thrombolytic activity (32.64%) in vitro [211]. A bark EO of *C. deodara* was proposed as a candidate for glaucoma treatment, but this was based on mere computational data, suggesting an interaction of several of its ingredients with a variety of biological receptors [280].

EOs obtained from leaves, cones, and stems of *P. halepensis* were reported in several publications to have phytotoxic (inhibiting seed germination and seedling growth) and herbicidal effects [281]. Cone EOs of *P. brutia*, *P. nigra* subsp. *laricio*, and *P. pinea* [146], as well as the leaf EO of *Larix decidua* [282], have phytotoxic effects against *Phalaris canariensis* and *Sinapis arvensis*.

In a small number of cases, an antioxidant effect was directly claimed to be the main mechanism involved in an observed pharmacological effect or at least a key contributor to this effect. For instance, a cytoprotective effect of a *P. halepensis* leaf EO against aspirin was claimed to be related to the antioxidant properties of the natural product, as evidenced by changes in SOD and CAT activities [283]. The beneficial effects of a *P. halepensis* needle EO observed in rat models of Alzheimer’s disease were also attributed (based on experimental evidence) to its antioxidant effects [96,284]. In other cases, though, it is difficult to establish whether a connection exists between the observed pharmacological effects and the antioxidant properties of an EO or its ingredients, or mechanisms independent of the antioxidant effects are proposed. For instance, based on molecular dockings (with its known limitations), it was suggested that germacrene D-4-ol (from *P. nigra* EOs) would act by inhibiting FtsZ, a tubulin homolog in bacteria [74], a leaf EO of *P. koraiensis* would act on *S. aureus* by inhibiting specific bacterial regulatory genes involved in pathogenicity [158], while a *P. sylvestris* EO would inhibit beta-lactamase, as suggested by molecular ligand docking [177].

Concerning safety, it was reported that a *P. sylvestris* EO (part not specified) induces somatic mutations in *Drosophila* but exhibits significantly lower genotoxicity when tested on human lymphocytes. The authors inferred that pinpointing the specific compounds accountable for the genotoxic effects of EOs would facilitate the creation of EOs devoid of these constituents, thus enhancing their safety [285]. Seed and cone EOs from *A. concolor* exerted no cytotoxicity on normal human cells (skin fibroblasts and microvascular endothelial cells) when used in concentrations of up to 1 μL/mL [286]. For cone EOs of *Picea pungens* and *Picea orientalis*, the safety levels on human skin fibroblasts were around 0.075 μL/mL, whereas on HMEC-1 cells, they were around 0.005 μL/mL. Among the two EOs, the one from *Picea orientalis* had stronger effects on cell viability, whereas the cone EO from *Picea pungens* had stronger effects on DNA synthesis [287]. For a branch and leaf EO of *C. atlantica*, an LD50 of 500 mg/kg was estimated in rats after a single dose administration [90]. At a dose of 2.5 mL/rat, a *C. deodara* root oil caused a number of modifications in the liver and kidney of the animals (fatty changes, some congestion and a few inflammatory cells in the liver, atrophic changes, slight edema and inflammatory cells in the kidney) [288].

The pharmacological investigations of EOs obtained from Pinaceae have been limited to a small number of species, mainly *Pinus*. It is rather sad that in an era where humanity has embarked on an unprecedented knowledge adventure in the fields of genomics, epigenomics, proteomics, metabolomics, and other “omics”, research on plant metabolites remains behind, very fragmented, and scarce. We could not identify any evaluation of EOs derived from six Pinaceae genera. In the future, it is expected that both chemical characterization and pharmacological exploration will extend to new species and new EOs (from parts other than those investigated up to date) using better methodologies.

## 8. Conclusions

EOs derived from various Pinaceae species have often been evaluated for their antioxidant effects, mostly using in vitro and, to a very limited extent, ex vivo systems. We identified 70 species from which EOs have been at least once evaluated for their antioxidant effects. By far, most of these species belonged to the genus *Pinus*, followed by *Abies*, *Picea*, *Cedrus*, and *Larix*. Thus, antioxidant data were available for genera from both the pinoid (*Pinus*, *Picea*, *Larix*) and abietoid (*Abies*, *Cedrus*) clade genera. However, for six genera (*Cathaya*, *Pseudotsuga*, *Keteleeria*, *Tsuga*, *Nothotsuga*, and *Pseudolarix*), no EO seems to have been evaluated for antioxidant effects.

Using relative potencies and estimating conversion factors from one comparator to another allowed us to compare EOs when IC_50_ values were computed with different reference substances. Only a limited subset (six) of essential oils (EOs) derived from Pinaceae species exhibit greater potency than ascorbic acid as antioxidants. Among these, four EOs originate from the *Pinus* genus, one from *Abies*, and one from *Cedrus*. Notably, three of the *Pinus* EOs were extracted from the wood of a singular species (*Pinus pinaster* Aiton) using various modern extraction techniques, as reported in a single publication [100]. The remaining *Pinus* EO was obtained from the needles of *Pinus thunbergii* Parl. [77]. Additionally, a leaf EO sourced from *Cedrus deodara* (Roxb. ex D.Don) G.Don demonstrated approximately threefold greater activity compared to ascorbic acid [70]. However, a wide variability in the way in which various antioxidant methods are applied has been identified, as well as in the results for EOs obtained from the same plant species and part; therefore, the relevance of such a ranking should not be overestimated. Differences might be partially due to differences in extraction methods but also to different influences of pedo-climate factors and the age and growth stage of the plant. Anyway, the wide differences observed in the properties of EOs obtained from the same plant species and part, often by the same authors (and thus controlling at least to some extent for the variability in the extraction method), suggest that epigenetic factors have a large contribution in driving the chemical composition and biological effects of an EO, in addition to the genetic traits of the species. The wide variability observed in the antioxidant effects emphasizes the need for standardization of EOs by those involved in marketing such products so as to ensure that batch-to-batch variations are sufficiently small. This review also emphasizes the need for more work in standardizing the way in which the antioxidant effects of EOs are investigated and reported. There is also a need for more research in correlating the chemical composition of EOs (known to be complex and variable) and the antioxidant effects measured through various testing methods.

## Figures and Tables

**Table 1:** Common language terms describing antioxidant activity based on the quantitative criterion of RP values.

RP Values	Common Language Term Describing Antioxidant Activity
<0.1	Very strong
0.1 < RP < 1.0	Strong
1 < −RP < 10	Moderate
10 < RP < −100	Weak
RP > 100	Very weak or inactive

**Table 2:** Conversion of relative potencies (RPs) estimated with different positive controls for DPPH results.

RP to Convert	Conversion Ratio	Mean Ratio	References
alpha-tocopherol ⇨ ascorbic acid	100.44/47.58 (=2.11); 0.1/0.04 (=2.5)	2.305	[52,123]
BHA ⇨ ascorbic acid:	0.093/0.054 (1.72); 3.7/2 (1.85)	1.785	[74,85]
BHT ⇨ ascorbic acid:	23.71/63.04 (=0.376); 21.51/53.24 (=0.404)	0.39	[58,59]

**Table 3:** IC_50_ values and relative potencies against ascorbic acid for the antioxidant effects of EOs from Pinaceae estimated on DPPH.

Taxon	Main Chemical Constituents	IC_50_ Sample/IC_50_ Ascorbic Acid	RP (Ascorbic Acid)
Leaf			
*Abies pindrow* (Royle ex. D.Don) Royle [47]	Limonene (38.9%), α-pinene (36.5%), β-pinene (6.9%), and α-selinene (4.4%)	8.07 μg/mL/3.27 μg/mL	2.47
*Cedrus deodara* (Roxb. ex D.Don) G.Don [70]	α-terpineol (30.2%), linalool (24.47%), limonene (17.01%), anethole (14.57%), caryophyllene (3.14%), and eugenol (2.14%)	0.53 μg/mL/1.75 μg/mL	0.30
*Pinus gerardiana* Wall. ex D.Don [69]	α-pinene (46.8%), 3-carene (24%), caryophyllene (9.1%), and α-phellandrene (3.9%)	54.8 μg/mL/19 μg/mL	2.88
*Pinus halepensis* Mill. [97]	β-caryophyllene (28.04%), myrcene (23.81%), and α-pinene (12.02%)	113.25 μg/mL/22.61 μg/mL	5.00
*Pinus nigra* ssp. *nigra* [74]	α-pinene (45.93%), germacrene D (27.50%), β-caryophyllene (8.13%), β-pinene (6.90%), and germacrene D-4-ol (0.57%)	25.596 mg/mL/0.054 mg/mL	474.0
*Pinus nigra* ssp. *pallasiana* (Lamb.) Holmboe [74]	α-pinene (42.33%), germacrene D (30.59%), β-caryophyllene (7.43%), β-pinene (5.15%), and germacrene D-4-ol (1.93%)	28.677 mg/mL/0.054 mg/mL	531.06
*Pinus nigra* ssp. *nigra* (syn. *P. nigra* var. *banatica* Georgescu & Ionescu) [74]	α-pinene (50.83%), germacrene D (23.69%), β-caryophyllene (7.31%), β-pinene (3.10%), and germacrene D-4-ol (0.01%)	25.08 mg/mL/0.054 mg/mL	464.4
*Pinus pinaster* Aiton (two producers) [73]	α-pinene (44.6%, 36.5%), β-pinene (23.0%, 18.8%), β-caryophyllene (5.0%, 8.7%), β-myrcene (5.0%, 5.9%), germacrene-D (1.7%, 5.6%), limonene (3.9%, 3.3%), and δ-3-carene (2.1%, 1.8%).	No activity	No activity
*Pinus pinaster* Aiton [71]	α-pinene (13.53%), β-caryophyllene (15.46%), abietadiene (10.81%), β-pinene (9.81%), rimuen (9.13%), abietatriene (8.36%), α-amorphene (6.91%), cupressene (5.21%) β-myrcene (4.14%), α-humulene (2.70%), and δ-cadinene (1.52%)	145.8 μg/mL/6.25 μg/mL	23.33
*Pinus pinaster* Aiton [73]	α-pinene (27.0%), β-pinene (28.0%), β-myrcene (11.0%), δ-3-carene (6.6%), germacrene-D (6.3%), β-caryophyllene (4.5%), and limonene (4.5%)	55.2 mg/mL/0.04 mg/mL	1380
*Pinus pinea* L. [101]	α-pinene (0.51%), β-pinene (0.36%), limonene (11.42%), β-caryophyllene (7.61%), germacrene-D (5.52%), δ-selinene (4.14%), guaiol (12.70%), α-eudesmol (5.19%), and manoyl oxide (3.61%)	45.1 μg/mL/5.0 μg/mL	9.02
*Pinus pinea* L. [73]	limonene (72.8%) and α-pinene (7.6%)	195.7 mg/mL/0.04 mg/mL	4892.5
*Pinus roxburghii* Sarg. [69]	α-terpinene (50.9%), α-ocimene (25.4%), caryophyllene (19.5%), 3-carene (17.8%), α-pinene (12.7%), humulene (3.1%), and thujopsene (3.1%)	67.3 μg/mL/19 μg/mL	3.54
*Pinus wallichiana* A.B.Jacks. [69]	A-pinene (36.0%), sesquisabinene hydrate (10.4%), cadinol (2.2%), and limonene (2.1%)	69.8 μg/mL/19 μg/mL	3.67
*Pinus wallichiana* A.B.Jacks. [72]	β-pinene (46.8%), α-pinene (25.2%), myrecene (2.5%), α-terpineol (2.3%), and caryophyllene oxide (2.1%)	28.8 μg/mL/11.5 μg/mL	2.50
Leaf and twig			
*Abies alba* Mill. [85]	Bornyl acetate (30.31%), camphene (19.81%), 3-carene (13.85%), tricyclene (12.90%), and limonene (7.50%)	27,000 μg/mL/2 μg/mL	13,500
Wood/sawdust			
*Cedrus atlantica* (Endl.) G.Manetti ex Carrière [52]	β-himachalene (28.99%), α-himachalene (14.43%), longifolene (12.2%), α-bisabolene (7.71%), α-atlantone (4.81%), deodarone (4.18%), and δ-cadinene (3.65%)	54.19 μg/mL/47.58 μg/mL	1.14
*Cedrus atlantica* (Endl.) G.Manetti ex Carrière (Itzer forest) [99]	β-himachalene (27.67%), α-himachalene (12%), 11αH-himachal-4-en-1β-ol (9.42%), cadina-1(6), 4 diene (8.45%), and 6-camphenol (3.16%)	15.559 mg/mL/0.08 mg/mL	194.49
*Cedrus atlantica* (Endl.) G.Manetti ex Carrière (Senoual Forest) [99]	β-himachalene (44.23%), α-himachalene (16.69%), cadina-1(6) 4 diene (11.27%), 6-camphenol (4.54%), and 11αH-himachal-4-en-1β-ol (1.31%)	16.264 mg/mL/0.08 mg/mL	203.3
*Pinus pinaster* Aiton [71]	α-pinene (58.44%), junipene (6.10%), α-terpineol (5.32%), and limonene (4.09%)	113.45 μg/mL/6.25 μg/mL	18.15
Cone			
*Pinus armandii* Franch. [58]	α-pinene (20.92%), limonene (15.78%), β-pinene (4.91%), and pinocarveol (4.76%)	378.51 μg/mL/63.04 μg/mL	6
*Pinus koraiensis* Siebold & Zucc. [59]	α-pinene (35.2%), limonene (18.4%), β-pinene (8.7%), β-caryophyllene (3.5%), and myrcene (3.0%)	242.39 μg/mL/53.24 μg/mL	4.55
*Pinus pinaster* Aiton [71]	α-pinene (32.57%), β-pinene (27.39%), junipene (9.45%), δ-3-carene (7.32%), limonene (3.54%), and β-myrcene (3.20%)	85.82 μg/mL/6.25 μg/mL	13.73
*Pinus pinea* L. [101]	Limonene (32.56%), α-pinene (6.78%), β-pinene (4.66%), and caryophyllene oxide (3.73%)	40.5 μg/mL/5 μg/mL	8.1
*Pinus sylvestris* L. [59]	aromadendrene (20.2%), α-pinene (18.5%), α-longipinene (10.5%), α-terpineol (5.5%), caryophyllene oxide (3.6%), limonene (3.3%), and pinocarveol (3.0%)	365.61 μg/mL/53.24 μg/mL	6.87
Bark			
*Pinus gerardiana* Wall. ex D.Don [69]	3-carene (31.1%), α-cubebene (16.4%), α-pinene (16.3%), α-phellandrene (5.9%), isoledene (4.6%), and bornyl acetate (4.0%)	71.2 μg/mL/19 μg/mL	3.75
*Pinus pinea* L. [101]	β-caryophyllene (16.51%), limonene (14.83%), caryophyllene oxide (11.83%), longifolene (7.51%), and guaiol (3.13%)	48.4 μg/mL/5 μg/mL	9.68
*Pinus pumila* (Pall.) Regel [77]	α-pinene (23.61–32.53%), limonene (9.21–12.67%), camphene (9.12–13.4%), longifolene (5.85–13.23%), β-pinene (2.92–4.58%), δ-cadinene (2.47–4.62%), and bornyl acetate (2.20–4.52%)	15.26 mg/mL/0.04 mg/mL 14.89 mg/mL/0.04 mg/mL 14.63 mg/mL/0.04 mg/mL	381.5 372.25 365.75
*Pinus wallichiana* A.B.Jacks. [69]	3-carene (43.2%), α-pinene (30.2%), cadinol (3.5%), and limonene (3.2%)	58.4 μg/mL/19 μg/mL	3.07
*Pinus roxburghii* Sarg. [69]	3-carene (22.5%), 4-carene (6.2%), limonene (4.9%), longifolene (4.7%), and α-pinene (3.4%),	51.7 μg/mL/19 μg/mL	2.72
Wood tar			
*Cedrus atlantica* (Endl.) G.Manetti ex Carrière (from Itzer forest) [99]	β-himachalene (24.05%), α-himachalene (13.76%), methyl-1,4 cyclohexadiene (9.06%), cadina-1 (6), 4 diene (7.65%), and 6-camphenol (8.76%)	0.126 mg/mL/0.084 mg/mL	1.5
*Cedrus atlantica* (Endl.) G.Manetti ex Carrière (from Senoual Forest) [99]	β-himachalene (24.25%), α-himachalene (1.15%), methyl-1,4 cyclohexadiene (13.56%), cadina-1 (6), 4 diene (7.37%), 6-camphenol (8.76%), and sabinene hydrate (5.92%)	0.143 mg/mL/0.084 mg/mL	1.7

**Table 4:** IC_50_ values, relative potencies against alpha-tocopherol, and equivalent RP values against ascorbic acid for the antioxidant effects of EOs from Pinaceae estimated on DPPH.

Taxon	Main Chemical Constituents	IC_50_ Sample/IC_50_ Alpha-Tocopherol	RP (Alpha-Tocopherol)	RP (Ascorbic Acid)
Leaf				
*Abies numidica* de Lannoy ex Carrière [45]	Caryophyllene (17.31%), α-pinene (10.59%), 2,2,6,10-tetramethylbicyclo [5.4.0] undeca-9,11-diene (8.65%), linalylacetate (8.42%), 2,6-octadiene, 2,6-dimethyl (7.63%), β-selinene (7.28%), sabinene (6.88%), β-pinene (5.45%), camphene (3.72%)	IC_50_ could not be estimated because of the very low effect	Weak/inactive	Weak/inactive
*Pinus densiflora* Siebold & Zucc. [76]	Camphene (22.38%), α-pinene (20.58%), α-limonene (20.16%), bornyl acetate (9.79%), β-pinene (6.73%), δ-3-carene (4.36%)	120 μg/mL/12.6 μg/mL	9.52 (moderate)	21.94
*Pinus nigra* J. F. Arnold ssp. *mauritanica* (Mair. & Pay) [64]	β-caryophyllene (26.2%), germacrene D (17.2%), α-pinene (9.4%), kaur-16-ene (7.1%), δ-cadinene (6.9%), α-humulene (4.1%)	260.93 mg/mL/0.142 mg/mL	1837 (very weak)	4234.285
*Pinus thunbergii* Parl. [76]	α-terpinolene (19.3%), δ-3-carene (16.77%), β-phellandrene (13.36%), α-pinene (10.91%), γ-terpinene (6.25%), 4-terpineol (5.35%), sabinene (5.15%), α-terpinene (4.22%), β-pinene (3.96%)	30 μg/mL/12.6 μg/mL	2.38 (moderate)	5.49
Twigs				
*Pinus nigra* J. F. Arnold ssp. *mauritanica* (Mair. & Pay) [64]	α-pinene (55.7%), kaur-16-ene (12.4%), β-pinene (2.3%), cembrene (2.3%)	93.72 mg/mL/0.142 mg/mL	660 (very weak)	1521.3
Wood				
*Cedrus atlantica* (Endl.) G.Manetti ex Carrière [52]	β-himachalene (28.99%), α-himachalene (14.43%), α-bisabolene (7.71%), α-atlantone (4.81%), deodarone (4.18%)	54.19 μg/mL/100.44 μg/mL	0.54 (strong)	1.245
Bark				
*Pinus pumila* (Pall.) Regel (three samples with different extraction methods) [77]	α-pinene (23.61–32.53%), limonene (9.21–12.67%), camphene (9.12–13.4%), longifolene (5.85–13.23%), β-pinene (2.92–4.58%), δ-cadinene (2.47–4.62%), bornyl acetate (2.20–4.52%)	15.26 mg/mL/0.1 mg/mL 14.89 mg/mL/0.1 mg/mL 14.63 mg/mL/0.1 mg/mL	152.6 (very weak) 148.9 (very weak) 146.3 (very weak)	351.7 343.2 337.2

**Table 5:** IC50 values, relative potencies against BHA, and equivalent RP values against ascorbic acid for the antioxidant effects of EOs from Pinaceae estimated on DPPH.

Taxon (Main Chemical Constituents of the EO)	IC_50_ Sample/ IC_50_ BHA	RP (BHA)	RP (Ascorbic Acid)
Leaf			
***Pinus cembra* L.** [79] (α-pinene—69.14%, limonene + β-phellandrene—4.64%, α-cadinene—3.71%)	19.93 mg/mL/0.0033 mg/mL	6039	10,779.61
***Pinus halepensis* Mill.** [67] (myrcene 17.5–21.6%, β-caryophyllene 17.3–21.2%, *p*-cymene 7.9–11.9%, α-pinene 8.5–12.9%, caryophyllene oxide 5.4–12.6%)	201.28 μg/mL/23.57 μg/mL to 236.18 μg/mL/23.57 μg/mL	8.54 to 10.02	15.24–17.89
***Pinus nigra* J. F. Arnold ssp. *dalmatica* (Vis.)** [80] (α-pinene—24%, β-pinene—16.0%, germacrene D—14.6%, β-caryophyllene, bornyl acetate—3.3%, limonene—3.3%)	EC50 estimation could not be performed due to very low activity	Very weak	Very weak
***Pinus nigra* ssp. *nigra*** [74] (α-pinene—45.93%, germacrene D (27.50%), β-caryophyllene—8.13%, β-pinene—6.90)	25.596 mg/mL/0.093 mg/mL	275.23	491.29
***Pinus nigra* ssp. *pallasiana* (Lamb.) Holmboe** [74] (α-pinene—42.33%, germacrene D—30.59%, β-caryophyllene —7.43%, β-pinene—5.15%)	28.677 mg/mL/0.093 mg/mL	308.35	550.40
***Pinus nigra* ssp. *nigra* (syn. *P. nigra* var. *banatica* Georgescu & Ionescu)** [74] (α-pinene—50.83%, germacrene D—23.69%, β-caryophyllene—7.31%, β-pinene—3.10%)	25.08 mg/mL/0.093 mg/mL	269.68	481.38
Leaf and twig			
***Abies alba* Mill.** [85] Bornyl acetate (30.31%), camphene (19.81%), 3-carene (13.85%), tricyclene (12.90%), limonene (7.50%)	27,000 μg/mL/3.7 μg/mL	7297.30	13,025.68

**Table 6:** IC50 values, relative potencies against BHT, and equivalent RP values against ascorbic acid for the antioxidant effects of EOs from Pinaceae estimated on DPPH.

Taxon (Main Chemical Constituents of the EO)	IC_50_ Sample/ IC_50_ BHT	RP (BHT)	RP (Ascorbic Acid)
Leaf			
***Abies balsamea* (L.) Mill.** [44] (β-pinene—31.1%, α-pinene—14.4%, δ-3-carene—13.6%, bornyl acetate—9.1%, limonene—8.5%, β-phellandrene—6.8%, camphene—5.5%)	The IC_50_ value could not be determined due to the observed low activity	Very weak	Very weak
***Abies numidica* de Lannoy ex Carrière** [54] (Caryophyllene—17.31%, α-pinene—10.59%, 2,2,6,10-tetramethylbicyclo [5.4.0] undeca-9,11-diene—8.65%, 2,6-octadiene, 2,6-dimethyl—7.63%, linalylacetate—7.42%, β-selinene—7.28%, sabinene—6.88%, β-pinene—5.45%, camphene—3.72%) *	0.288 mg/mL/0.143 mg/mL	2.01	0.78
***Abies numidica* de Lannoy ex Carrière** [45] (Caryophyllene—17.31%, α-pinene—10.59%, 2,2,6,10-tetramethylbicyclo [5.4.0] undeca-9,11-diene—8.65%, 2,6-octadiene, 2,6-dimethyl—7.63%, linalylacetate—7.42%, β-selinene—7.28%, sabinene—6.88%, β-pinene—5.45%, camphene—3.72%)	IC_50_ could not be estimated because of the very low effect	Weak/inactive	Weak/inactive
***Larix laricina* (Du Roi) K. Koch** [44] (Bornyl acetate—16.4%, α-pinene—16.1%, camphene—13.4%, limonene—13.2%, β-pinene—12.2%, camphor—5.7%, δ-3-carene—5.4%, myrcene—4.0%)	The IC_50_ value could not be determined due to the observed low activity	Very weak	Very weak
***Picea glauca* (Moench) Voss** [44] (β-pinene—15.1%, bornyl acetate—14.6%, camphor—14.5%, α-pinene—13.7%, limonene—12.7%, camphene—12.6%)	The IC_50_ value could not be determined due to the observed low activity	Very weak	Very weak
***Picea mariana* Britton, Sterns, & Poggenb.** [44] (Bornyl acetate—29.2%, α-pinene—15.3%, camphene—17.8%, δ-3-carene—8.5%, limonene—4.9%, β-pinene—4.7%)	80.3 mg/mL/0.02 mg/mL	4015	1565.85
***Pinus halepensis* Mill.** [81] (Caryophyllene—28.57–48.77%, phenethyl isovalerate—3.59–22.22%, α-humulene—5.34–9.24%, α-pinene—4.63–16.1%, β-myrcene—3.70–15.55%, sabinene—0.7–5.14%, α-terpinolene—1.22–5.61%, 3(Z)-cembrene A—n.d.–12.64%)	73,030 μg/mL/34.23 μg/mL to 270,860 μg/mL/34.23 μg/mL	2133.5 to 7912.94	832.06 to 3086.05
***Pinus banksiana* Lamb.** [44] (α-pinene—38.2%, β-pinene—17.8%, δ-3-carene—8.2%, limonene 8.1%, myrcene—6.4%, camphene—3.1%, camphor—2.8%, bornyl acetate—2.6%)	7 mg/mL/0.02 mg/mL	350	136.5
***Pinus densiflora* Siebold & Zucc.** [76] (Camphene—22.38%, α-pinene—20.58%, α-limonene—20.16%, bornyl acetate—9.79%, β-pinene—6.73%, δ-3-carene—4.36%, 2,3-dimethylbicyclo[2.2.1]hept-2-ene—4.35%, 1,1,7-trimethyltricyclo[2.2.1.0(2.6)]heptane—4.01%)	120 μg/mL/14.3 μg/mL	8.39	3.27
***Pinus halepensis* Mill.** [124] (Caryophyllene—15.87%, β-pinene—13.74%, α-pinene—12.5%, cembrene—9.84%, α-humulene—9.19%, β-phenylethyl isovalerate—7.89%, trans-β-ocimene—6.65%, 1R-α-pinene—3.68%)	0.41 mg/mL/0.12 mg/mL	3.42	1.33
***Pinus halepensis* Mill.** [67] (Myrcene—17.5–21.6%, (z)-β-caryophyllene—17.3–21.2%, α-pinene—8.5–12.9%, p-cymene 7.9–11.9%, caryophyllene oxide—5.4–12.6%)	201.28 μg/mL/31.41 μg/mL to 236.18 μg/mL/31.41 μg/mL	6.41 to 7.52	2.50 to 2.93
***Pinus thunbergii* Parl.** [76] (α-terpinolene—19.3%, δ-3-carene —16.77%, β-phellandrene —13.36%), α-pinene—10.91%, γ-terpinene—6.25%, 4-terpineol—5.35%, sabinene—5.15%, α-terpinene—4.22%, β-pinene—3.96%)	30 μg/mL/14.3 μg/mL	2.10	0.819
**Leaf and twig**			
***Abies alba* Mill.** [85] (Bornyl acetate—30.31%, camphene—19.81%, 3-carene—13.85%, tricyclene—12.90%, dl-limonene—7.50%)	27,000 μg/mL/39.3 μg/mL	687.02	267.94
**Wood/sawdust**			
***Pinus pinaster* Aiton** [100] **(EOs obtained through five methods)** (β-caryophyllene—21.2–30.1%, longifolene—8.9–14.4%, α-caryophyllene—3.7–5.2%, α-muurolen—1.6–3.7%, nerolidol—1.4–4.7%, patchouli alcohol—n.d.–6.3%, limonene—0.1–6.9%, α-terpineol—2.5–12.4%, anethol—n.d.–4.7%)	123.0 μg/mL/24.0 μg/mL 115.2 μg/mL/24.0 μg/mL 59.8 μg/mL/24.0 μg/mL 15.0 μg/mL/24.0 μg/mL 15.4 μg/mL/24 μg/mL	5.125 4.8 2.49 0.625 0.64	2.00 1.87 0.97 0.24 0.25
Cone			
***Pinus armandii* Franch.** [58] (α-pinene—20.92%, D-limonene—15.78%, β-pinene—4.91%, trans-pinocarveol—4.76%)	378.51 μg/mL/23.71 μg/mL	15.96	6.22
***Pinus koraiensis* Siebold & Zucc.** [59] (α-pinene—35.2%, limonene—18.4%, β-pinene—8.7%, β-caryophyllene—3.5%, myrcene—3.0%)	242.39 μg/mL/21.51 μg/mL	11.27	4.39
***Pinus sylvestris* L.** [59] (Aromadendrene—20.2%, α-pinene—18.5%, α-longipinene—10.5%, α-terpineol—5.5%, caryophyllene oxide—3.6%, limonene—3.3%, trans-pinocarveol—3.0%)	365.61 μg/mL/21.51 μg/mL	17.00	6.63
Bark			
***Abies nordmanniana* ssp. *equi-trojani* (Asch. & Sint. ex Boiss.) Coode & Cullen** [42] (α-terpineol—5.4%, abietadien—4.2%, manoyl oxide—4.0%, dehydroabietal—3.9%, 4-terpineol—3.8%, octadecadienoic acid—3.2%)	5480 μg/mL/9.8 μg/mL	559.18	218.08
***Cedrus libani* A. Rich** [42] (Manool—11.0%, isolongifolene—9.7%, abietate 3.1%, dehydro-p-cymene—2.5%, camphene 2.4%, berbenone—2.3%, borneol—2.0%)	440 μg/mL/9.8 μg/mL	44.90	17.51
***Pinus nigra* J. F. Arnold** [42] (Docosane—8.0%, octadec-9-en-18-olide—4.7%, p-xylene—3.6%, hexacosane 3.1%)	1970 μg/mL/9.8 μg/mL	201.02	78.40

* Chemical composition reported based on [45] because the primary reference did not include it.

**Table 7:** Ranking of EOs from Pinaceae based on the RP (ascorbic acid).

Taxon [Reference]	RP (Ascorbic_Acid)	Plant Part
***Pinus pinaster* Aiton** [100] **(microwave hydrodiffusion and gravity)** (β-caryophyllene—21.2%, longifolene—9.8%, α-caryophyllene—3.8%, α-muurolen—1.6%, nerolidol—1.4%, patchouli alcohol—1.4%, limonene—0.1%, α-terpineol—10.0%, anethol—4.7%)	0.24	Wood (sawdust)
***Pinus pinaster* Aiton** [100] **(solvent-free microwave extraction)** (β-caryophyllene—22.2%, longifolene—10.0%, α-caryophyllene—3.7%, α-muurolen—1.6%, nerolidol—1.4%, patchouli alcohol—0.9%, limonene—0.3%, α-terpineol—8.8%, anethol—3.3%)	0.25	Wood (sawdust)
***Cedrus deodara* (Roxb. ex D.Don) G.Don** [70] (α-terpineol—30.2%, linalool—24.47%, limonene—17.01%, anethole—14.57%, caryophyllene—3.14%, and eugenol—2.14%)	0.3	Leaf
***Abies numidica* de Lannoy ex Carrière** [54] (Caryophyllene—17.31%, α-pinene—10.59%, 2,2,6,10-tetramethylbicyclo [5.4.0] undeca-9,11-diene—8.65%, 2,6-octadiene, 2,6-dimethyl—7.63%, linalylacetate—7.42%, β-selinene—7.28%, sabinene—6.88%, β-pinene—5.45%, camphene—3.72%) *	0.78	Leaf
*Pinus thunbergii* Parl. [76] (α-terpinolene—19.3%, δ-3-carene—16.77%, β-phellandrene—13.36%, α-pinene—10.91%, γ-terpinene—6.25%, 4-terpineol—5.35%, sabinene—5.15%, α-terpinene—4.22%, β-pinene—3.96%)	0.819	Leaf
***Pinus pinaster* Aiton** [100] **(ultrasound-assisted extraction HD)** (β-caryophyllene—24.0%, longifolene—8.9%, α-caryophyllene—4.4%, α-muurolen—1.9%, nerolidol—3.1%, patchouli alcohol—0.4%, limonene—6.9%, α-terpineol—10.7%, anethol—2.5%)	0.97	Wood (sawdust)
***Cedrus atlantica* (Endl.) Manetti ex Carriere** [52] (β-himachalene—28.99%, α-himachalene—14.43%, longifolene—12.2%, α-bisabolene—7.71%, (Z)-α-atlantone—4.81%, deodarone—4.18%, δ-cadinene—3.65%)	1.14	Wood
***Cedrus atlantica* (Endl.) Manetti ex Carriere** [52]	1.245	Wood
***Pinus halepensis* Mill.** [124] (Caryophyllene—15.87%, β-pinene—13.74%, α-pinene—12.5%, cembrene—9.84%, α-humulene—9.19%, β-phenylethyl isovalerate—7.89%, trans-β-ocimene—6.65%, 1R-α-pinene—3.68%)	1.33	Leaf
***Cedrus atlantica* (Endl.) G.Manetti ex Carrière (from Itzer forest)** [99] (β-himachalene—24.05%, α-himachalene—13.76%, methyl-1,4 cyclohexadiene—9.06%, trans-cadina-1 (6), 4 diene—7.65%, 6-camphenol—8.76%)	1.5	Wood tar
***Cedrus atlantica* (Endl.) G.Manetti ex Carrière (from Senoual Forest)** [99] (β-himachalene—24.25%, α-himachalene—1.15%, trans-cadina-1 (6), 4 diene—7.37%, 6-camphenol—8.76%, cis-sabinene hydrate—5.92%)	1.7	Wood tar
***Pinus pinaster* Aiton** [100] **(turbohydrodistillation)** (β-caryophyllene—28.0%, longifolene—12.6%, α-caryophyllene—5.2%, α-muurolen—2.9%, nerolidol—4.7%, patchouli alcohol—n.d., limonene—0.8%, α-terpineol—12.4%, anethol—n.d.)	1.87	Wood (sawdust)
***Pinus pinaster* Aiton** [100] **(hydrodistillation)** (β-caryophyllene—30.1%, longifolene—14.4%, α-caryophyllene—5.2%, α-muurolen—3.7%, nerolidol—3.4%, patchouli alcohol—6.3%, limonene—0.3%, α-terpineol—2.5%, anethol—n.d.)	2	Wood (sawdust)
***Abies pindrow* (Royle ex. D.Don) Royle** [47] (Limonene—38.9%, α-pinene—36.5%, β-pinene—6.9%, and α-selinene 4.4%)	2.47	Leaf
***Pinus wallichiana* A.B.Jacks.** [72] (β-pinene—46.8%, α-pinene—25.2%, myrecene—2.5%, α-terpineol—2.3%, caryophyllene oxide—2.1%)	2.5	Leaf
***Pinus gerardiana* Wall. ex D.Don** [69] (α-pinene—46.8%, 3-carene—24%, caryophyllene—9.1%, α-phennaldrene—3.9%)	2.88	Leaf
***Pinus wallichiana* A.B.Jacks.** [69] (3-carene—43.2%, α-pinene—30.2%, cadinol—3.5%, D-limonene—3.2%)	3.07	Bark
***Pinus densiflora* Siebold & Zucc.** [76] (Camphene—22.38%, α-pinene—20.58%, α-limonene—20.16%, bornyl acetate—9.79%, β-pinene—6.73%, δ-3-carene—4.36%)	3.27	Leaf
***Pinus roxburghii* Sarg.** [69] (α-terpinene—50.9%, α-ocimene—25.4%, caryophyllene—19.5%, 3-carene—17.8%, α-pinene—12.7%, humulene—3.1%, thujopsene—3.1%)	3.54	Leaf
*Pinus wallichiana* A.B.Jacks. [69]	3.67	Leaf
***Pinus gerardiana* Wall. ex D.Don** [69] (α-pinene—36.0%, sesquisabinene hydrate—10.4%, cadinol—2.2%, D-limonene—2.1%)	3.75	Bark
***Pinus koraiensis* Siebold & Zucc.** [59] (α-pinene—35.2%, limonene—18.4%, β-pinene—8.7%, β-caryophyllene—3.5%, myrcene—3.0%)	4.39	Cone
*Pinus koraiensis* Siebold & Zucc. [59]	4.55	Cone
***Pinus halepensis* Mill.** [97] (β-caryophyllene—28.04%, myrcene—23.81%, and α-pinene—12.02%)	5	Leaf
***Pinus thunbergii* Parl.** [76] (α-terpinolene—19.3%, δ-3-carene—16.77%, β-phellandrene—13.36%, α-pinene—10.91%, γ-terpinene—6.25%, 4-terpineol—5.35%, sabinene—5.15%, α-terpinene—4.22%, β-pinene—3.96%)	5.49	Leaf
***Pinus armandii* Franch.** [58] (α-pinene—20.92%, D-limonene—15.78%, β-pinene—4.91%, trans-pinocarveol—4.76%)	6	Cone
***Pinus armandii* Franch.** [58]	6.22	Cone
***Pinus sylvestris* L.** [59] (Aromadendrene—20.2%, α-pinene—18.5%, α-longipinene—10.5%, α-terpineol—5.5%, caryophyllene oxide—3.6%, limonene—3.3%, trans-pinocarveol—3.0%)	6.63	Cone
***Pinus sylvestris* L.** [59]	6.87	Cone
***Pinus pinea* L.** [101] (Limonene—32.56%, α-pinene—6.78%, β-pinene—4.66%, caryophyllene oxide—3.73%)	8.1	Cone
***Pinus pinea* L.** [101] (α-pinene—0.51%, β-pinene—0.36%, limonene—11.42%, β-caryophyllene—7.61%, germacrene-D—5.52%, δ-selinene—4.14%, guaiol—12.70%, α-eudesmol—5.19%, manoyl oxide—3.61%)	9.02	Leaf
***Pinus pinea* L.** [101] (β-caryophyllene—16.51%, limonene—14.83%, caryophyllene oxide—11.83%, longifolene—7.51%, guaiol—3.13%)	9.68	Bark
***Pinus pinaster* Aiton** [71] (α-pinene—32.57%, β-pinene—27.39%, ju-nipene—9.45%, δ-3-carene—7.32%, limonene—3.54%, β-myrcene—3.20%)	13.73	Cone
***Cedrus libani* A. Rich** [42] (Manool—11.0%, isolongifolene—9.7%, abietate 3.1%, dehydro-p-cymene—2.5%, camphene 2.4%, berbenone—2.3%, borneol L—2.0%)	17.51	Bark
***Pinus pinaster* Aiton** [71] (α-pinene—58.44%, junipene—6.10%, α-terpineol—5.32%, limonene—4.09%)	18.15	Wood (sawdust)
***Pinus densiflora* Siebold & Zucc.** [76] (Camphene—22.38%, α-pinene—20.58%, α-limonene—20.16%, bornyl acetate—9.79%, β-pinene—6.73%, δ-3-carene—4.36%)	21.94	Leaf
***Pinus pinaster* Aiton** [71] (α-pinene—27.0%, β-pinene—28.0%, β-myrcene—11.0%, δ-3-carene—6.6%, germacrene-D—6.3%, β-caryophyllene—4.5%, limonene—4.5%)	23.33	Leaf
***Pinus nigra* J. F. Arnold** [42] (Docosane—8.0%, octadec-9-en-18-olide—4.7%, 12-(cyanomethyl) indolo [1,2] quinazoline—3.7%, p-xylene—3.6%, hexacosane 3.1%)	78.4	Bark
*Pinus banksiana* Lamb. [44] (α-pinene—38.2%, β-pinene—17.8%, δ-3-carene—8.2%, limonene 8.1%, myrcene—6.4%, camphene—3.1%, camphor—2.8%, bornyl acetate—2.6%)	136.5	Leaf
***Cedrus atlantica* (Endl.) G.Manetti ex Carrière (Itzer forest)** [99] (β-himachalene—27.67%, α-himachalene—12%, 11αH-himachal-4-en-1β-ol—9.42%, trans-cadina-1 (6), 4 diene—8.45%, 6-camphenol—3.16%)	194.49	Wood (sawdust)
***Cedrus atlantica* (Endl.) G.Manetti ex Carrière (Senoual Forest)** [99] (β-himachalene—44.23%, α-himachalene—16.69%, trans-cadina-1 (6), 4 diene—11.27%, 6-camphenol—4.54%, 11αH-himachal-4-en-1β-ol—1.31%)	203.3	Wood (sawdust)
***Abies nordmanniana* ssp. *equi-trojani* (Asch. & Sint. ex Boiss.) Coode & Cullen** [42] (α-terpineol—5.4%, abietadien—4.2%, manoyl oxide—4.0%, dehydroabietal—3.9%, 4-terpineol—3.8%, octadecadienoic acid—3.2%)	218.08	Bark
***Abies alba* Mill.** [85] (Bornyl acetate—30.31%, camphene—19.81%, 3-carene—13.85%, tricyclene—12.90%, dl-limonene—7.50%)	267.94	Leaf and twig
***Pinus pumila* (Pall.) Regel (hydrodistillation with screw extrusion treatment—E-HD)** [77] (α-pinene—24.88%, longifolene 11.64%, limonene—10.13%, camphene—9.60%, (+)-δ-cadinene—4.52%, β-pinene—3.22%)	337.2	Bark
***Pinus pumila* (Pall.) Regel (microwave-assisted hydrodistillation with screw extrusion treatment—E-MHD)** [77] (α-pinene—32.53%, camphene—13.40%, longifolene 5.85%, limonene—12.67%, β-pinene—4.58%, bornyl acetate—4.52%, (+)-δ-cadinene—2.47%)	343.2	Bark
***Pinus pumila* (Pall.) Regel (microwave-assisted hydrodistillation with pulverization treatment—P-MHD)** [77] (α-pinene—23.61%, longifolene 13.23%, limonene—9.21%, camphene—9.12%, (+)-δ-cadinene—4.62%, β-pinene—2.92%)	351.7	Bark
*Pinus pumila* (Pall.) Regel [77] **(P-MHD method)**	365.75	Bark
*Pinus pumila* (Pall.) Regel [77] **(E-MHD method)**	372.25	Bark
*Pinus pumila* (Pall.) Regel [77] **(E-HD method)**	381.5	Bark
***Pinus nigra* ssp. *nigra* (syn. *P. nigra* var. *banatica* Georgescu & Ionescu)** [74] (α-pinene—50.83%, germacrene D—23.69%, (E)-caryophyllene—7.31%, β-pinene—3.10%, germacrene D-4-ol—0.01%))	464.4	Leaf
***Pinus nigra* ssp. *nigra*** [74] (α-pinene—45.93%, Germacrene D—27.50%, (E)-caryophyllene—8.13%, β-pinene—6.90%, germacrene D-4-ol—0.57%)	474	Leaf
***Pinus nigra* ssp. *nigra* (syn. *P. nigra* var. *banatica* Georgescu & Ionescu)** [74]	481.38	Leaf
***Pinus nigra* ssp. *nigra*** [74]	491.29	Leaf
***Pinus nigra* ssp. *pallasiana* (Lamb.) Holmboe** [74] (α-pinene—42.33%, germacrene D—30.59%, (E)-caryophyllene—7.43%, β-pinene—5.15%, germacrene D-4-ol—1.93%))	531.06	Leaf
***Pinus nigra* ssp. *pallasiana* (Lamb.) Holmboe** [74]	550.4	Leaf
*Pinus pinaster* Aiton [73] (α-pinene—27.0%, β-pinene—28.0%, β-myrcene—11.0%, δ-3-carene—6.6%, germacrene-D—6.3%, β-caryophyllene—4.5%, limonene—4.5%)	1380	Leaf
***Pinus nigra* J. F. Arnold ssp. *mauritanica* (Mair. & Pay)** [64] (α-pinene—55.7%, kaur-16-ene—12.4%, β-pinene—2.3%, cembrene—2.3%)	1521.3	Twig
***Picea mariana* Britton, Sterns & Poggenb.** [44] (Bornyl acetate—29.2%, α-pinene—15.3%, camphene—17.8%, δ-3-carene—8.5%, Limonene—4.9%, β-pinene—4.7%)	1565.85	Leaf
***Pinus nigra* J. F. Arnold ssp. *mauritanica* (Mair. & Pay)** [64] (β-caryophyllene—26.2%, germacrene D—17,2%, α-pinene—9.4%, kaur-16-ene—7.1%, δ-cadinene—6.9%, α-humulene—4.1%)	4234.285	Leaf
***Pinus pinea* L.** [73] (Limonene (72.8%) α-pinene (7.6%))	4892.5	Leaf
***Pinus cembra* L.** [79] (α-pinene—69.14%, limonene + β-phellandrene—4.64%, α-cadinene—3.71%)	10,779.61	Leaf
***Abies alba* Mill.** [85] (3-carene—13.85%, camphene—19.81%, tricyclene—12.90%, dl-limonene—7.50%, bornyl acetate—30.31%)	13,025.68	Leaf and twig
***Abies alba* Mill.** [85]	13,500	Leaf and twig
***Pinus halepensis* Mill.** [67] (Myrcene 17.5–21.6%, (Z)-β-caryophyllene 17.3–21.2%, p-cymene 7.9–11.9%, α-pinene 8.5–12.9%, caryophyllene oxide 5.4–12.6%)	15.24–17.89	Leaf
***Pinus halepensis* Mill.** [67]	2.50 to 2.93	Leaf
***Pinus halepensis* Mill.** [81] (Caryophyllene—28.57–48.77%, phenethyl isovalerate—3.59–22.22%, α-humulene—5.34–9.24%, α-pinene—4.63–16.1%, β-myrcene—3.70–15.55%, sabinene—0.7–5.14%, α-terpinolene—1.22–5.61%, cembrene A—n.d.–12.64%)	832.06 to 3086.05	Leaf
***Pinus pinaster* Aiton (two producers)** [73] (α-pinene—44.6% and 36.5%, β-pinene—23.0% and 18.8%, β-caryophyllene—5.0% and 8.7%, β-myrcene—5.0% and 5.9%, germacrene-D—1.7% and 5.6%, limonene—3.9% and 3.3%, δ-3-carene—2.1% and 1.8%)	No activity	Leaf
***Abies balsamea* (L.) Mill.** [44] (β-pinene—31.1%, α-pinene—14.4%, δ-3-carene—13.6%, bornyl acetate—9.1%, limonene—8.5%, β-phellandrene—6.8%, camphene—5.5%)	Very weak	Leaf
***Larix laricina* (Du Roi) K. Koch** [44] (Bornyl acetate—16.4%, α-pinene—16.1%, camphene—13.4%, limonene—13.2%, β-pinene—12.2%, camphor—5.7%, δ-3-carene—5.4%, myrcene—4.0%)	Very weak	Leaf
***Picea glauca* (Moench) Voss** [44] (β-pinene—15.1%, bornyl acetate—14.6%, camphor—14.5%, α-pinene—13.7%, limonene—12.7%, camphene—12.6%)	Very weak	Leaf
***Pinus nigra* J. F. Arnold ssp. *dalmatica* (Vis.)** [80] (α-pinene—24%, β-pinene—16.0%, germacrene D—14.6%, β-caryophyllene, bornyl acetate—3.3%, limonene—3.3%)	Very weak	Leaf
***Abies numidica* de Lannoy ex Carrière** [45] (Caryophyllene—17.31%, α-pinene—10.59%, 2,2,6,10-tetramethylbicyclo [5.4.0] undeca-9,11-diene—8.65%, 2,6-octadiene, 2,6-dimethyl—7.63%, linalylacetate—7.42%, β-selinene—7.28%, sabinene—6.88%, β-pinene—5.45%, camphene—3.72%) *	Very weak	Leaf

* Chemical composition reported based on [45] because the primary reference did not include it.

**Table 8:** Intra-study comparisons of antioxidant activity of EOs derived from Pinaceae species evaluated on DPPH using a variety of endpoints.

Reference	Plant Part	Increasing Order of Antioxidant Effects
Sharma et al. (2020) [69]	Bark	*Pinus gerardiana* < *Pinus wallichiana* < *Pinus roxburghii*
Ulukanli et al. (2014) [75]	Bark oleoresin	*Pinus pinea* < *Pinus brutia* var. *brutia*
Efremov et al. (2021) [125]	Branch	*Abies sibirica* < *Pinus sibirica*
Kurti et al. (2019) [92]	Branch and leaf	*Pinus peuce* < *Pinus heldreichii* < *Pinus sylvestris* < *Pinus mugo* < *Pinus nigra*
Yang et al. (2010) [59]	Cone	*Pinus sylvestris* L. < *Pinus koraiensis*
Wajs-Bonikowska et al. (2015) [56]	Cone	*Abies koreana* E.H. Wilson < *Abies alba*
Ruas et al. (2022) [73]	Leaf	*Pinus pinaster* < *Pinus pinea* < *Pinus pinaster* *
Sharma et al. (2020) [69]	Leaf	*Pinus wallichiana* < *Pinus roxburghii* < *Pinus gerardiana*
Sarac et al. (2014) [74]	Leaf	*Pinus nigra* ssp. *pallasiana* < *P. nigra* ssp. *nigra* < *P. nigra* var. *banatica*
Poaty et al. (2015) [44]	Leaf	*Abies balsamea* < *Picea mariana* < *Picea glauca* < *Pinus banksiana*
Yener et al. (2014) [78]	Leaf	*Pinus pinea* ≈ *Pinus halepensis* < *Pinus brutia* < *Pinus nigra* **
Aloui et al. (2021) [83]	Leaf	*Pinus pinaster* < −*Pinus halepensis* < −*Pinus pinea*
Fkiri et al. (2019) [65]	Leaf	*Pinus pinaster* ssp. *escarena* (Risso) K.Richt. *(syn. Pinus pinaster* var. *maghrebiana*) < *Pinus pinaster* ssp. *renoui* (Villar) Maire
Garzoli et al. (2021) [39]	Leaf	*Picea abies* L. ≈ *Pinus cembra* < *Abies alba* < *Pinus mugo*
Liu et al. (2022) [48]	Leaf	*Pinus armandii* < *Pinus strobus* L. < *Pinus bungeana* Zucc. ex. Endl. < *Pinus sylvestris* var. *mongholica* Litv. < *Pinus yunnanensis* Franch. < *Pinus koraiensis* < *Pinus massoniana* Lamb. < *Pinus tabuliformis* var. *mukdensis* (Uyeki ex. Nakai) Uyeki < *Pinus tabuliformis* Carrière < *Pinus densata*
Xie et al. (2015) [40]	Leaf	*Pinus massoniana* < *Pinus* henryi Mast. < *Pinus tabuliformis* < *Pinus tabuliformis* var. *umbraculifera* Liou & Z.Wang < *Pinus tabuliformis* f. *shekanensis* < *Pinus tabuliformis* var. *mukdensis*
Kačániová et al. (2014) [87]	NA (probably leaves)	*Pinus mugo* (syn. *P. montana*) < *Abies alba* < *Pinus sylvestris*
Wajs-Bonikowska et al. (2015) [56]	Seed	*Abies alba* < *Abies koreana*
Kolayli et al. (2009) [42]	Trunk bark	*Abies nordmanniana* ssp. *equi-trojani* < −*Pinus nigra* < −*Cedrus libani*
Venditti et al. (2022) [51]	Wood	*Pinus pinea* < −*Cedrus libani*

* One sample of *P. pinaster* EO had more potent antioxidant effects than one sample of *P. pinea*. Two samples of *P. pinaster* EO from different producers were entirely inactive, whereas a third was more active than that from *P. pinea.* ** All EOs had shallow antioxidant effects compared with BHT and BHA.

**Table 9:** Intra-study comparisons of antioxidant activity of EOs derived from Pinaceae species evaluated on ABTS using various endpoints.

Reference	Plant Part	Increasing Order of Antioxidant Effects
Jo et al. (2018) [94]	Branch	*Abies holophylla* Maxim. < (*Abies koreana* ≈ *Pinus densiflora* for. multicaulis)
Garzoli et al. (2021) [39]	Leaf	*Picea abies* ≈ *Pinus cembra* < *Abies alba* < *Pinus mugo*
Xie et al. (2015) [40]	Leaf	*Pinus massoniana* < *Pinus henryi* < *Pinus tabuliformis* f. *shekanensis* < *Pinus tabuliformis* < *Pinus tabuliformis* var. *umbraculifera* < *Pinus tabuliformis* var. *mukdensis*
Liu et al. (2022) [48]	Leaf	*Pinus armandii* < *Pinus bungeana* < *Pinus strobus* < *Pinus sylvestris* var. *mongholica* < *P. tabuliformis* var. *mukdensis* < *Pinus massoniana* < *Pinus yunnanensis* < *Pinus koraiensis* < *Pinus densata* < *Pinus tabuliformis*.
Sarac et al. (2014) [74]	Leaf	*Pinus nigra* ssp. *pallasiana* < *P. nigra* ssp. *nigra* < *P. nigra* ssp. *nigra* (syn. *P. nigra* var. *banatica*)
Jo et al. (2018) [94]	Leaf	(*Picea abies* & *Abies nephrolepis* Maxim. & *Picea koraiensis* Nakai) < *Pinus densiflora*
Venditti et al. (2022) [51]	Wood	*Pinus pinea* < *Cedrus libani*

**Table 10:** Intra-study comparisons of antioxidant activity of EOs derived from Pinaceae species evaluated on FRAP using various endpoints.

Reference	Plant Part	Increasing Order of Antioxidant Effects
Ulukanli et al. (2014) [75]	Bark oleoresin	*Pinus pinea* < *Pinus brutia* var. *brutia*
Xie et al. (2015) [40]	Leaf	*Pinus massoniana* < *Pinus henryi* < *Pinus tabuliformis* var. *umbraculifera* < *Pinus tabuliformis* f. *shekanensis* < *Pinus tabuliformis* < *Pinus tabuliformis* var. *mukdensis*
Liu et al. (2022) [48]	Leaf	*Pinus armandii* < *Pinus strobus* < *P. tabulaeformis* var. *mukdensis* < *Pinus bungeana* < *Pinus massoniana* < *Pinus sylvestris* var. *mongholica* < *Pinus koraiensis* < *Pinus yunnanensis* < *Pinus tabulaeformis* < *Pinus densata*
Ustun et al. (2012) [98]	Leaf	*Pinus brutia* < *Pinus sylvestris* < *Pinus pinea* ≈ *Pinus nigra* < *Pinus halepensis* *
Yener et al. (2014) [78]	Leaf	*Pinus pinea* < *Pinus halepensis* < *Pinus brutia* < *Pinus nigra*
Ustun et al. (2012) [98]	Twigs	*Pinus brutia* < *Pinus sylvestris* ≈ *Pinus nigra* < *Pinus pinea* < *Pinus halepensis* *
Venditti et al. (2022) [51]	Wood	*Pinus pinea* < *Cedrus libani*

* Based on the FRAP values at the highest concentration tested (1000 μg/mL).

**Table 11:** IC_50_ and RP (beta-carotene) for the fresh leaf EOs of the 46 *Pinus* species analyzed by Koutsaviti et al. (2021) [120,126].

Species	IC_50_	RP (Beta-Carotene)	Section (Subsection)
***Pinus canariensis* C. Sm.** (Germacrene D—44.0%, α-pinene—14.6%, β-caryophyllene—8.7%, limonene—7.9%, myrcene—6.4%, δ-cadinene—4.1%)	1.00 ± 0.08	4.35	*Pinus* (*Pinaster*)
***Pinus attenuata* Lemmon** (α-pinene −38.1%, germacrene D—29.0%, β-caryophyllene—7.9%, δ-cadinene—5.4%)	1.30 ± 0.02	5.65	*Trifoliae* (Australes)
***Pinus muricata* D.Don** (Germacrene D—41.5%, α-pinene—17.3%, β-ocimene—5.4%, δ-3-carene—5.3%, β-pinene—4.8%, α-cadinol—4.0%)	1.60 ± 0.09	6.96	*Trifoliae* (Australes)
***Pinus sylvestris* var. sylvestris (syn. *Pinus sylvestris* ssp. *scotica* (Beissn.) E.F.Warb.)** (Isoabienol—25.9%, δ-3-carene—10.7%, germacrene D-4-ol—10%, α-pinene—9.5%, β-pinene—5.7%, germacrene D—5.1%, α-cadinol—4.1%, bicyclogermacrene—3.2%, δ-cadinene—3.5%)	1.67 ± 0.05	7.26	*Pinus* (*Pinus*)
***Pinus halepensis* Mill.** (β-caryophyllene—19.0%, myrcene—15.1%, α-pinene—8.0%, cembrene 6.5%, phenyl ethyl 3-methyl butanoate—5.1%, α-humulene 3.8%)	1.78 ± 0.17	7.74	*Pinus* (*Pinaster*)
***Pinus mugo* var. *prostrata*** (Bornyl acetate—14.1%, α-pinene—12.9%, camphene—6.5%, δ-3-carene—6.4%, germacrene D-4-ol—6.0%, bicyclogermacrene—5.7%, β-elemene—3.7%, α-cadinol—3.5%, β-caryophyllene—3.4%)	1.79 ± 0.21	7.78	*Pinus* (*Pinus*)
***Pinus mugo* Turra** (α-pinene—13.7%, germacrene D—12.1%, δ-3-carene—9.9%, myrcene—6.9%, germacrene D-4-ol—6.1%, β-caryophyllene—5.3%, bicyclogermacrene—4.2%, bornyl acetate—3.8%, terpinolene—3.8%)	1.89 ± 0.16	8.22	*Pinus* (*Pinus*)
***Pinus monticola* Douglas ex. D.Don** (β-elemene—15.0%, α-pinene—14.9%, β-pinene—14.2%, germacrende D-4-ol—7.2%, germacrene D—6.8%, β-phellandrene—6.0%, α-cadinol—4.2%)	1.94 ± 0.09	8.43	*Quinquefoliae* (Strobus)
***Pinus nigra* ssp. *nigra*** (Germacrene D—32.1%, α-pinene—19.5%, β-caryophyllene—16.1%, β-pinene—12.1%, α-humulene—3.2%, limonene—3.1%)	2.05 ± 0.20	8.91	*Pinus* (*Pinus*)
***Pinus rigida* Mill.** (β-pinene—16.7%, germacrene D—15.5%, bicyclogermacrene—14.1%, β-phellandrene—13.6%, α-pinene—4.7%, α-cadinol—4.7%, germacrene D-4-ol—4.4%, β-caryophyllene—4.3%, myrcene—3.7%,)	2.09 ± 0.12	9.09	*Trifoliae* (Australes)
***Pinus wallichiana* A.B.Jacks.** (β-pinene—18.1%, α-pinene—13.8%, germacrene D—10.3%, β-caryophyllene—7.2%, germacrene D-4-ol—6.7%, unidentified compound—6.1%, δ-cadinene—4.6%, α-cadinol—4.3%)	2.23 ± 0.12	9.70	*Quinquefoliae* (Strobus)
***Pinus cembra* L.** (Germacrene D—21.2%, α-pinene—21.1%, β-phellandrene—13.5%, bicyclogermacrene—4.7%, β-pinene—4.6%, δ-cadinene—4.3%, methyl daniellate—4.1%)	2.36 ± 0.05	10.26	*Quinquefoliae* (Strobus)
***Pinus cembroides* Zucc.** (α-pinene—30.9%, β-caryophyllene—19.2%, germacrene D—9.4%, β-pinene—5.6%, myrcene—5.0%, β-phellandrene—3.7%, camphene—3.5%, α-humulene—3.2%, α-cadinol—3.0%)	2.38 ± 0.16	10.35	*Parrya* (*Cembroides*)
***Pinus coulteri* D. Don.** (4-*epi*-isocembrol—17.7%, unidentified compound—16.9%, α-pinene—13.6%, germacrene D—8.8%, β-phellandrene—6.2%, α-cadinol—4.7%, δ-cadinene 3.6%)	2.64 ± 0.08	11.48	*Trifoliae* (*Ponderosae*)
***Pinus thunbergii* Parl.** (Germacrene D—18.7%, β-phellandrene—14.5%, β-pinene 13.2%, β-caryophyllene—9.4%, α-pinene—8.7%, myrcene—5.4%, δ-cadinene—4.0%)	2.68 ± 0.12	11.65	*Pinus* (*Pinus*)
***Pinus strobiformis* Engelm.** (Germacrene D—25.5%, β-pinene—12.5%, α-cadinol—8.1%, α-pinene—8.0%, δ-cadinene—6.4%, bicyclogermacrene—4.1%, α-muurolol—3.2%)	2.68 ± 0.15	11.65	*Quinquefoliae* (Strobus)
***Pinus koraiensis* Siebold & Zucc.** (Germacrene D—18.7%, α-pinene—14.3%, β-caryophyllene—8.4%, bornyl acetate—8.3%, terpinolene—6.6%, camphene—6.1%, limonene—5.4%, δ-cadinene—4.8%)	2.73 ± 0.07	11.87	*Quinquefoliae* (Strobus)
***Pinus ponderosa* Douglas ex. C.Lawson** (β-pinene—45.0%, α-pinene—22.5%, δ-3-carene—12.0%, β-phellandrene—4.4%)	2.86 ± 0.09	12.43	*Trifoliae* (*Ponderosae*)
***Pinus nigra subsp. pallasiana* (Lamb.) Holmboe (syn. *P nigra* ssp. *caramanica* (Loudon) Rehder)** (β-pinene—20.7%, germacrene D—20.0%, α-pinene—18.0%, β-caryophyllene—7.8%, limonene—3.1%)	3.28 ± 0.27	14.26	*Pinus* (*Pinus*)
***Pinus mugo* Turra (syn. *Pinus mugo* var. *pumilio* (Haenke) Zenari)** (α-pinene—14.1%, germacrene D—8.0%, bornyl acetate—7.6%, β-caryophyllene—6.8%, camphene—6.3%, δ-cadinene—4.6%, α-cadinol—4.4%, sabinene 4.3%, germacrene D-4-ol—4.1%, myrcene—3.2%, limonene—3.0, sylvestrene—3.0%)	3.42 ± 0.06	14.87	*Pinus* (*Pinus*)
***Pinus contorta* var. *murrayana* (Balf.) S.Watson** (β-phellandrene—47.0%, α-pinene—4.8%, (Z)-β-ocimene—4.6%, bicyclogermacrene—3.8%, α-cadinol—3.3%, β-pinene—3.0%)	3.51 ± 0.16	15.26	*Trifoliae* (Contorte)
***Pinus banksiana* Lamb.** (Bornyl acetate—15.7%, germacrene D—14.7%, α-pinene—8.2%, β-pinene—7.8%, myrcene—6.3%, δ-3-carene—3.2%)	3.60 ± 0.14	15.65	*Trifoliae* (Contorte)
***Pinus flexilis* E. James** (α-pinene—24.5%, germacrene D—12.2%, camphene—9.0%, β-pinene—8.6%, unidentified compound—6.2%, bornyl acetate—3.8%, α-cadinol—3.4%, δ-cadinene—3.3%)	3.62 ± 0.57	15.74	*Quinquefoliae* (Strobus)
***Pinus jeffreyi* A.Murray bis** (α-pinene—29.8%, unidentified compound—18.3%, germacrene D—11.5%, β-pinene—4.7%, δ-cadinene—4.0%, β-phellandrene—3.4%, α-cadinol—3.4%)	3.72 ± 0.39	16.17	*Trifoliae* (*Ponderosae*)
***Pinus elliottii* Engelm.** (Germacrene D—24.5%, β-pinene—12.9%, α-pinene—10.6%, β-caryophyllene—6.6%, δ-cadinene—3.8%, α-cadinol—3.7%, α-terpineol—3.2%)	3.97 ± 0.35	17.26	*Trifoliae* (Australes)
***Pinus tabuliformis* Carrière** (β-caryophyllene—15.9%, germacrene D—14.5%, α-pinene—9.7%, β-pinene—6.2%, bornyl acetate 4.7%, δ-cadinene—4.5%, bicyclogermacrene—4.3%, myrcene—4.1%, α-cadinol—4.0%, α-humulene—3.4%, 4-isocembrol—3.1%)	3.97 ± 0.62	17.26	*Pinus* (*Pinus*)
***Pinus peuce* Griseb.** (α-pinene—30.1%, germacrene D—17.0%, camphene—5.9%, β-pinene—10.8%, β-caryophyllene—9.8%, bornyl acetate—6.5%, sylvestrene—4.7%)	4.04 ± 0.26	17.57	*Quinquefoliae* (Strobus)
***Pinus nigra* ssp. *salzmannii* (Dunal) Franco** (Germacrene D—18.5%, β-caryophyllene—17.8%, α-pinene—12.2%, limonene—12.5%, β-pinene—4.1%, myrcene—4.0%, α-humulene 3.9%)	4.05 ± 0.12	17.61	*Pinus* (*Pinus*)
***Pinus pumila* (Pall.) Regel** (δ-3-carene—20.8%, α-pinene—17.0%, terpinolene—6.0%, limonene—4.6%, germacrene D-4-ol—4.4%, 3-α-hydroxy-manool—4.1%, camphene—3.5%, β-phellandrene—3.0%, β-caryophyllene—3.0%)	4.24 ± 0.27	18.43	*Quinquefoliae* (Strobus)
***Pinus pinea* L.** (Limonene—31.7%, abienol—12.3%, sylvestrene—7.9%, α-pinene—5.8%, germacrene D—4.6%, guaiol—4.0%, methyl levopimarate 3.2%)	4.40 ± 0.37	19.13	*Pinus* (*Pinaster*)
***Pinus densiflora* Siebold & Zucc.** (Cadina-1(6),4-diene—12.7%, α-pinene—9.9%, β-ocimene—8.9%, β-caryophyllene—8.7%, isocembrol—8.5%, β-pinene—7.1%, bornyl acetate—3.7%)	4.45 ± 0.40	19.35	*Pinus* (*Pinus*)
***Pinus brutia* Ten.** (α-pinene—20.3%, β-pinene—31.2%, germacrene D—12.8%, β-caryophyllene—11.7%)	4.67 ± 0.14	20.30	*Pinus* (*Pinaster*)
***Pinus sylvestris* L.** (α-pinene—34.4%, δ-cadinene—7.1%, β-pinene—6.8%, bicyclogermacrene—6.0%, β-caryophyllene—4.7%, γ-cadinene—3.8%, germacrene D—3.7%, α-cadinol—3.5%)	4.86 ± 0.48	21.13	*Pinus* (*Pinus*)
***Pinus armandii* Franch** (α-pinene—48.0%, germacrene D—19.0%, β-caryophyllene—14.3%)	4.95 ± 0.17	21.52	*Quinquefoliae* (Strobus)
***Pinus bungeana* Zucc. ex. Endl.** (α-pinene—21.1%, germacrene D—11.2%, β-caryophyllene—11.0%, γ-muurolene—10.0%, camphene—8.3%, β-pinene—5.6%, δ-cadinene—4.7%, limonene—4.6%)	4.99 ± 0.14	21.70	*Quinquefoliae* (*Gerardianae*)
***Pinus contorta* var. *contorta*** (β-phellandrene—19.9%, pimarinal—8.9%, α-cadinol—8.9%, δ-3-carene—7.4%, manool—7.4%, α-pinene—5.9%, terpinen-4-ol—5.2%, β-pinene—4.7%, methyl dehydroabietate—4.7%, α-muurolol—4.4%, γ-terpinene—3.7%, α-cadinol—3.4%, manool oxide—3.0%)	5.11 ± 0.40	22.22	*Trifoliae* (Contorte)
***Pinus nigra* ssp. *laricio*** (α-pinene—18.0%, δ-3-carene—16.1%, β-caryophyllene—13.9%, germacrene D—12.7%, limonene—9.7%, terpinolene—3.3%)	5.25 ± 0.19	22.83	*Pinus* (*Pinus*)
***Pinus teocote* Schied. ex. Schltdl. & Cham.** (α-pinene—33.3%, germacrene D—27.6%, β-caryophyllene—9.8%, β-pinene—7.8%, δ-3-carene—4.4%, sylvestrene—3.6%, limonene—3.0%)	5.36 ± 1.23	23.30	*Trifoliae* (Australes)
***Pinus patula* Schiede ex. Schltdl. & Cham.** (Germacrene D—21.3%, α-pinene—18.5%, β-phellandrene—14.7%, β-caryophyllene—11.8%, β-pinene—3.7%, δ-cadinene—3.0%)	5.63 ± 0.02	24.48	*Trifoliae* (Australes)
***Pinus radiata* D. Don.** (β-pinene—38.7%, α-pinene—18.9%, germacrene D—6.4%, β-phellandrene—5.0%, bicyclogermacrene—4.7%, β-ocimene—3.8%, δ-cadinene—3.6%)	5.65 ± 0.10	24.57	*Trifoliae* (Australes)
***Pinus pinaster* Aiton** (Isoabienol—19.1%, sclarene—18.0%, germacrene D—11.2%, α-pinene—5.2%, abienol—4.7%, β-elemene 4.5%, abietatriene—4.0%)	7.03 ± 1.12	30.57	*Pinus* (*Pinaster*)
***Pinus parviflora* Siebold & Zucc.** (β-phellandrene—31.9%, α-pinene—21.1%, germacrene D—12.8%, β-pinene—12.5%, camphene—4.5%, methyl daniellate—4.1%, bornyl acetate—3.0%)	7.04 ± 0.44	30.61	*Quinquefoliae* (Strobus)
***Pinus heldreichii* Christ.** (Limonene—23.7%, germacrene D—21.3%, δ-3-carene—18.6%, α-pinene—11.1%, β-caryophyllene—8.6%, β-pinene—3.6%)	7.26 ± 0.54	31.57	*Pinus* (*Pinaster*)
***Pinus massoniana* Lamb.** (α-pinene—26.9%, germacrene D—20.7%, β-pinene—16.3%, β-caryophyllene—11.6%, β-phellandrene—6.8%)	8.29 ± 0.41	36.04	*Pinus* (*Pinus*)
***Pinus sabiniana* Douglas** (α-pinene—61.6%, unidentified compound—12.1%, β-ocimene—5.2%, β-pinene—4.5%)	9.05 ±1.25	39.35	*Trifoliae* (*Ponderosae*)
***Pinus taiwanensis* Hayata** (β-phellandrene—15.6%, β-caryophyllene—14.9%, α-pinene—12.1%, β-pinene—11.5%, myrcene—7.4%, terpinolene—5.6%, germacrene D—5.2%, bornyl acetate—4.3%)	9.31 ± 0.29	40.48	*Pinus* (*Pinus*)
***Pinus contorta* var. *latifolia* Engelm.** (β-pinene—32.8%, β-phellandrene—26.0%, α-pinene—5.6%, β-ocimene—4.9%, δ-cadinene—8.4%)	9.57 ± 0.64	41.61	*Trifoliae* (Contorte)
*Pinus torreyana* Parry ex. Carrière (Isocembrol—55.7%, cembrene—12.7%, limonene—8.6%, thunbergol—7.7%)	9.58 ± 0.40	41.65	*Trifoliae* (*Ponderosae*)
***Pinus gerardiana* Wall. ex D.Don** (β-pinene—39.1%, α-pinene—26.4%, myrcene—5.7%, β-phellandrene—5.3%)	11.35 ± 2.03	49.35	*Quinquefoliae* (*Gerardianae*)
***Pinus strobus* L.** (α-pinene—14.7%, germacrene D—11.1%, unidentified compound—8.4%, β-pinene—5.8%, α-cadinol—5.5%, β-phellandrene—5.2%, δ-cadinene—3.5%, bicyclogermacrene—3.2%, unidentified compound—3.2%)	11.54 ± 3.27	50.17	*Quinquefoliae* (Strobus)
***Pinus culminicola* Andresen & Beaman** (α-pinene—33.6%, β-pinene—20.2%, β-phellandrene 16.9%, germacrene D—7.9%)	11.71 ± 2.17	50.91	*Parrya* (*Cembroides*)
***Pinus roxburghii* Sarg.** (β-caryophyllene—20.5%, δ3-carene—15.9%, terpinolene—10.5%, α-pinene—8.4%, sabinene 6.2%, cembrene—4.9%, α-humulene—4.2%, terpinene-4-ol—3.5%)	15.96 ± 1.45	69.39	*Pinus* (*Pinaster*)
***Pinus aristata* Engelm.** (δ-3-carene—38.4%, β-phellandrene—12.7%, thymol methyl ether—11.4%, terpinolene—8.6%, α-pinene—6.6%, β-pinene—6.4%, sabinene—3.1%)	16.39 ± 1.52	71.26	*Parrya* (Balfourianae)
***Pinus monophyla* Torr. & Frém.** (β-pinene—27.2%, α-pinene—18.7%, δ-cadinene—7.2%, limonene—6.8%, germacrene D—4.9%, myrcene—4.6%, β-phellandrene—4.6%, γ-cadinene—3.5%)	20.03 ± 2.77	87.09	*Parrya* (*Cembroides*)

**Table 12:** Intra-study comparisons of EOs derived from different Pinaceae plant parts.

Species (Reference)	Antioxidant Test	Increasing Order of Antioxidant Effects of EOs
*Abies alba* Mill. [56]	DPPH	seed < cone
*Abies koreana* E.H. Wilson [56]	DPPH	cone < seed
*Cedrus atlantica* (Endl.) G.Manetti ex Carrière [99]	FRAP	wood tar < saw dust
*Pinus halepensis* Mill. [91]	ABTS	twigs < bark < male inflorescences < mature cone < immature cone < adult leaf < juvenile leaf
*Pinus halepensis* Mill. [91]	Chelating activity	mature cone < twigs < juvenile leaf < immature cone < bark < adult leaf < male inflorescences
*Pinus halepensis* Mill. [91]	DPPH	juvenile leaf < twigs < cone < bark < male inflorescence < mature cone < adult leaf
*Pinus brutia* Ten. [98]	FRAP	twig < leaf
*Pinus cembra* L. [79]	DPPH	leaf < twigs *
*Pinus brutia* var. *eldarica* (Medw.) Silba (syn. *Pinus eldarica* Medw.) [127]	DPPH	leaf < pollen < bark
*Pinus gerardiana* Wall. ex D.Don [69]	DPPH	bark < leaf
*Pinus gerardiana* Wall. ex D.Don [69]	Nitric oxide radical scavenging	bark < leaf
*Pinus halepensis* Mill. [98]	FRAP	leaf < twig
*Pinus nigra* J. F. Arnold [98]	FRAP	twig < leaf
*Pinus nigra* Arn. ssp. *mauritanica* (Mair. & Pay) [64]	Beta-carotene bleaching	leaf < twigs *
*Pinus nigra* Arn. ssp. *mauritanica* (Mair. & Pay) [64]	DPPH	leaf < twigs
*Pinus nigra* Arn. ssp. *mauritanica* (Mair. & Pay) [64]	Total antioxidant activity (phosphomolybdenum method)	At lower concentrations, leaf EO exhibited slightly higher activity than twigs, whereas at higher concentrations, twigs’ EO was more active than that of leaves.
*Pinus pinaster* Aiton [71]	ABTS	wood < leaf < cone *
*Pinus pinaster* Aiton [71]	DPPH	leaf < wood < cone
*Pinus pinaster* Aiton [71]	FRAP	leaf < wood < cone
*Pinus pinea* L. [101]	Beta-carotene bleaching	bark < cone < leaf
*Pinus pinea* L. [101]	Chelating activity	bark < leaf < cone *
*Pinus pinea* L. [101]	DPPH	bark < leaf < cone *
*Pinus pinea* L. [101]	Nitric oxide radical scavenging	bark < cone < leaf
*Pinus pinea* L. [98]	FRAP	twig < leaf
*Pinus roxburghii* Sarg. [69]	Nitric oxide radical scavenging	leaf < bark
*Pinus roxburghii* Sarg. [69]	DPPH	leaf < bark
*Pinus roxburghii* Sarg. [82]	DPPH	leaf < wood < bark
*Pinus sylvestris* L. [98]	FRAP	twig < leaf
*Pinus wallichiana* A.B.Jacks. [69]	DPPH	leaf < bark
*Pinus wallichiana* A.B.Jacks. [69]	Nitric oxide radical scavenging	leaf < bark

* The differences among the EOs were small (of doubtful practical relevance).

**Table 13:** Summary of studies evaluating the impact of the extraction method on the antioxidant effects of EOs from Pinaceae.

Species (Reference)	Plant Part	Antioxidant Test	Increasing Order of Antioxidant Effects of EOs Depending on the Extraction Method
*Abies sachalinensis* (F.Schmidt) Mast. [43]	Leaf	DPPH	Steam distillation < SW3 * < SW10 * < SW7 *
*Abies sachalinensis* (F.Schmidt) Mast. [43]	Leaf	Folin–Ciocalteu	Steam distillation < SW3 * < SW10 * < SW7 *
*Pinus pinaster* Aiton [100]	Sawdust (stem and branches)	DPPH	HD (hydrodistillation) < THD (turbo hydrodistillation) < UAE-HD (ultrasound-assisted extraction hydrodistillation) < SFME (solvent-free microwave extraction) ≈ MHG (microwave hydrodiffusion and gravity)
*Pinus pinaster* Aiton [100]	Sawdust (stem and branches)	FRAP	HD < THD < UAE-HD < SFME ≈ MHG
*Pinus pumila* (Pall.) Regel [77]	Bark	DPPH	E-HD (hydrodistillation with screw extrusion treatment) < E-MHD (microwave-assisted hydrodistillation with screw extrusion treatment) < P-MHD (microwave-assisted hydrodistillation with pulverization treatment)
*Pinus pumila* (Pall.) Regel [77]	Bark	FRAP	E-HD < P-MHD < E-MHD
*Pinus pumila* (Pall.) Regel [77]	Bark	Reducing power	E-HD < P-MHD < E-MHD
*Pinus pumila* (Pall.) Regel [77]	Bark	β-carotene bleaching inhibition	P-MHD ≈ E-HD < E-MHD
*Pinus roxburghii* Sarg. [61]	Resin	DPPH	Supercritical fluid extraction, 40 °C, 80 bar < superheated steam (120 °C) < superheated steam (140 °C) < steam distillation < superheated steam (160 °C) **
*Pinus roxburghii* Sarg. [61]	Resin	FRAP	Steam distillation < supercritical fluid extraction, 40 °C, 80 bar < superheated steam (120 °C) < superheated steam (140 °C) < superheated steam (160 °C) **
*Pinus roxburghii* Sarg. [61]	Resin	Hydrogen peroxide scavenging activity	Superheated steam (140 °C) < supercritical fluid extraction, 40 °C, 80 bar) < superheated steam (120 °C) < steam distillation < superheated steam (160 °C) **

* SW followed by a number indicates the number of shockwave cycles used for the pretreatment of samples before using conventional steam distillation. ** These are the results as tabulated by the authors in the paper, but the full-text commentary states that, on the contrary, the highest antioxidant effect was observed for the superheated steam distillation at 120 °C (63.33 ± 0.47%) and the lowest for the superheated steam distillation at 160 °C (49.53%). We contacted the authors but have not yet received clarification.

**Table 14:** Correlation between results obtained in the same study with different antioxidant methods.

Assays Evaluated	Species	Reference	Sample Size (Number of Paired Observations)	Correlation Coefficient (r)
ABTS vs. ferrous ion-chelating activity	*Pinus halepensis* Mill.	[91]	7	−0.07
ABTS vs. FRAP	*Pinus pinaster* Aiton	[71]	3	−0.81
ABTS vs. FRAP	Six *Pinus* taxa	[40]	6	0.63
ABTS vs. FRAP	Ten *Pinus* taxa	[48]	10	0.82
ABTS vs. ^•^OH radical inhibition	*Pinus pinaster* Aiton	[71]	3	0.77
ABTS vs. reducing power	*Pinus halepensis* Mill.	[81]	11	−0.23
Beta-carotene bleaching vs. ferrous ion-chelating activity	*Pinus halepensis* Mill.	[68]	10	0.52
Beta-carotene bleaching vs. nitric oxide radical scavenging	*Pinus pinea* L.	[101]	3	0.97
DPPH vs. ABTS	*Pinus halepensis* Mill.	[91]	7	−0.10
DPPH vs. ABTS	*Pinus halepensis* Mill.	[95]	3	0.99
DPPH vs. ABTS	*Pinus halepensis* Mill.	[81]	11	0.45
DPPH vs. ABTS	*Pinus pinaster* Aiton	[71]	3	0.57
DPPH vs. ABTS	Six *Pinus* taxa	[40]	6	0.84
DPPH vs. ABTS	Ten *Pinus* taxa	[48]	10	0.66
DPPH vs. ABTS (IC_50_)	*Pinus cembra* L., *Pinus mugo* Turra, *Picea abies* L., and *Abies alba* Mill.	[39]	4	0.989
DPPH vs. ABTS (TEAC)	*Pinus cembra* L., *Pinus mugo* Turra, *Picea abies* L., and *Abies alba* Mill.	[39]	4	0.953
DPPH vs. beta-carotene bleaching	*Pinus halepensis* Mill.	[68]	10	0.98
DPPH vs. beta-carotene bleaching assay	*Pinus pinea* L.	[101]	3	0.71
DPPH vs. ferrous ion-chelating activity	*Pinus halepensis* Mill.	[91]	7	0.45
DPPH vs. ferrous ion-chelating activity	*Pinus halepensis* Mill.	[68]	10	0.51
DPPH vs. ferrous ion-chelating activity	*Pinus pinea* L.	[101]	3	0.99
DPPH vs. Folin–Ciocâlteu (TEAC vs. GAE)	*Abies sachalinensis*	[43]	4	0.986
DPPH vs. FRAP	*Cedrus atlantica* (Endl.) G.Manetti ex Carrière	[99]	4	0.97
DPPH vs. FRAP	*Pinus pinaster* Aiton	[71]	3	−0.95
DPPH vs. FRAP	*Pinus pinaster* Aiton	[100]	5	−0.97 **
DPPH vs. FRAP	*Pinus roxburghii* Sarg.	[61]	5	0.17 ***
DPPH vs. FRAP	Six *Pinus* taxa	[40]	6	0.90
DPPH vs. FRAP	Ten *Pinus* taxa	[48]	10	0.60
DPPH vs. hydrogen peroxide scavenging	*Pinus roxburghii* Sarg.	[61]	5	0.49 *** (0.70 Spearman)
DPPH vs. linoleic acid system	*Pinus roxburghii* Sarg.	[61]	5	0.05 ***
DPPH vs. nitric oxide radical scavenging	*Pinus pinea* L.	[101]	3	0.53
DPPH vs. ^•^OH radical inhibition	*Pinus pinaster* Aiton	[71]	3	0.96
DPPH vs. reducing power	*Pinus halepensis* Mill.	[68]	10	0.99
DPPH vs. reducing power	*Pinus halepensis* Mill.	[81]	11	0.43
DPPH vs. TBARS	*Pinus mugo* Turro	[46]	4	0.99
Ferrous ion-chelating vs. beta-carotene bleaching assay	*Pinus pinea* L.	[101]	3	0.81
Ferrous ion-chelating vs. nitric oxide radical scavenging	*Pinus pinea* L.	[101]	3	0.66
FRAP vs. ^•^OH	*Pinus pinaster* Aiton	[71]	3	−0.99
Hydrogen peroxide scavenging vs. FRAP	*Pinus roxburghii* Sarg.	[61]	5	0.11 ***
Linoleic acid system vs. FRAP	*Pinus roxburghii* Sarg.	[61]	5	0.96 ***
Linoleic acid system vs. hydrogen peroxide scavenging	*Pinus roxburghii* Sarg.	[61]	5	0.16 ***
Reducing power vs. beta-carotene bleaching	*Pinus halepensis* Mill.	[68]	10	0.98
Reducing power vs. ferrous ion-chelating activity	*Pinus halepensis* Mill.	[68]	10	0.48
TPC * vs. ABTS	Ten *Pinus* taxa	[48]	10	0.92
TPC * vs. DPPH	Ten *Pinus* taxa	[48]	10	0.68
TPC * vs. FRAP	Ten *Pinus* taxa	[48]	10	0.96

* TPC = total phenolic content. ** We estimated this based on the five IC_50_ values corresponding to five EOs obtained by the authors through different methods. In the original paper, they reported R2 values based on the individual data point in the assessment of each EO, and the values reported had R2 (coefficients of determination) of 0.98–0.99. Still, the slope was negative for four out of five regression equations. *** Whereas the vast majority of values reported here correlated to IC_50_ values, in this study, inhibition percentages caused by a single concentration of the EO were available and correlated.

## Data Availability

The data presented in this study are available as a Supplementary Material (Tables S1–S43).

## Supplementary Materials

The following supporting information can be downloaded at https://www.mdpi.com/article/10.3390/antiox13030286/s1: Table S1 and Figure S1. ABTS vs. Ferrous ion-chelating activity (*Pinus halepensis* Mill.]); Table S2 and Figure S2. ABTS vs. FRAP (*Pinus pinaster* Aiton); Table S3 and Figure S3. ABTS vs. FRAP (six *Pinus* taxa); Table S4 and Figure S4. ABTS vs. FRAP (Ten *Pinus* taxa); Table S5 and Figure S5. ABTS vs. ^•^OH -radical (*Pinus pinaster* Aiton); Table S6 and Figure S6. ABTS vs. reducing power (*Pinus halepensis* Mill.); Table S7 and Figure S7. Beta-carotene bleaching vs. Ferrous ion-chelating activity (Pinus halepensis Mill.); Table S8 and Figure S8. Beta-carotene bleaching vs. Nitric oxide radical scavenging (*Pinus pinea* L.); Table S9 and Figure S9. DPPH vs. ABTS (*Pinus halepensis* Mill.); Table S10 and Figure S10. DPPH vs. ABTS (*Pinus halepensis* Mill.); Table S11 and Figure S11. DPPH vs. ABTS (*Pinus halepensis* Mill.]); Table S12 and Figure S12. DPPH vs. ABTS (*Pinus pinaster* Aiton); Table S13 and Figure S13. DPPH vs. ABTS (Six *Pinus* taxa); Table S14 and Figure S14. DPPH vs. ABTS (Ten *Pinus* taxa); Table S15 and Figure S15. DPPH vs. ABTS (*Pinus cembra* L., *Pinus mugo* Turra, *Picea abies* L., and *Abies alba* Mill.); Table S16 and Figure S16. DPPH vs. ABTS (TEAC) (*Pinus cembra* L., *Pinus mugo* Turra, *Picea abies* L., and *Abies alba* Mill.); Table S17 and Figure S17. DPPH vs. Beta-carotene bleaching (*Pinus halepensis* Mill.); Table S18 and Figure S18. DPPH vs. beta-carotene bleaching assay (*Pinus pinea* L.); Table S19 and Figure S19. DPPH vs. Ferrous ion-chelating activity (*Pinus halepensis* Mill.); Table S20 and Figure S20. DPPH vs. Ferrous ion-chelating activity (*Pinus halepensis* Mill.); Table S21 and Figure S21. DPPH vs. ferrous ion-chelating activity (*Pinus pinea* L.); Table S22 and Figure S22. DPPH vs. Folin-Ciocâlteu (TEAC vs. GAE) (*Abies sachalinensis*,); Table S23 and Figure S23. DPPH vs. FRAP (*Cedrus atlantica* (Endl.) G.Manetti ex Carrière); Table S24 and Figure S24. DPPH vs. FRAP (*Pinus pinaster* Aiton); Table S25 and Figure S25. DPPH vs. FRAP (*Pinus pinaster* Aiton,); Table S26 and Figure S26. DPPH vs. FRAP (*Pinus roxburghii* Sarg.); Table S27 and Figure S27. DPPH vs. FRAP (Six *Pinus* taxa); Table S28 and Figure S28. DPPH vs. FRAP (Ten *Pinus* taxa); Table S29 and Figure S29. DPPH vs. Hydrogen peroxide scavenging (*Pinus roxburghii* Sarg.); Table S30 and Figure S30. DPPH vs. Linoleic acid system (*Pinus roxburghii* Sarg.); Table S31 and Figure S31. DPPH vs. Nitric oxide radical scavenging (*Pinus pinea* L.); Table S32 and Figure S32. DPPH vs. ^•^OH-radical inhibition (*Pinus pinaster* Aiton); Table S33 and Figure S33. DPPH vs. reducing power (*Pinus halepensis* Mill.); Table S34 and Figure S34. DPPH vs. reducing power (*Pinus halepensis* Mill.); Table S35 and Figure S35. DPPH vs. TBARS (*Pinus mugo* Turro, [47]); Table S36 and Figure S36. Ferrous ion-chelating vs. beta-carotene bleaching assay (*Pinus pinea* L.); Table S37 and Figure S37. Ferrous ion-chelating vs. Nitric oxide radical scavenging (*Pinus pinea* L.); Table S38 and Figure S38. FRAP vs. OH (*Pinus pinaster* Aiton); Table S39 and Figure S39. Hydrogen peroxide scavenging vs. FRAP (Pinus roxburghii Sarg.); Table S40 and Figure S40. Linoleic acid system vs. FRAP (*Pinus roxburghii* Sarg.); Table S41 and Figure S41. Linoleic acid system vs. Hydrogen peroxide scavenging (*Pinus roxburghii* Sarg.); Table S42 and Figure S42. Reducing power vs. Beta-carotene bleaching (*Pinus halepensis* Mill.); Table S43 and Figure S43. Reducing power vs. Ferrous ion-chelating activity (*Pinus halepensis* Mill.).
