# Supplementary material for: Antioxidant Activity of Essential Oils from Pinaceae Species
Source: Antioxidants (Basel). 2024 Feb 26;13(3):286. doi: 10.3390/antiox13030286 (PMC10967544; doi:10.3390/antiox13030286)
Supplement: Supplementary file 1 [file antioxidants-13-00286-s001.zip › antioxidants-2864350-supplementary.pdf]

Table S1 and Figure S1. ABTS vs. Ferrous ion-chelating activity (*Pinus halepensis* Mill. [91])

Antioxidant endpoint	Test (Assay)
0.722 ± 0.031	ABTS
1.177 ± 0.057	ABTS
1.149 ± 0.013	ABTS
1.148 ± 0.07	ABTS
2.52 ± 0.022	ABTS
0.536 ± 0.036	ABTS
0.525 ± 0.023	ABTS
267.434 ± 2.36	Chelat
266.437 ± 1.04	Chelat
214.804 ± 0.99	Chelat
139.985 ± 0.8	Chelat
198.912 ± 1.56	Chelat
216.292 ± 0.33	Chelat
164.319 ± 2.71	Chelat

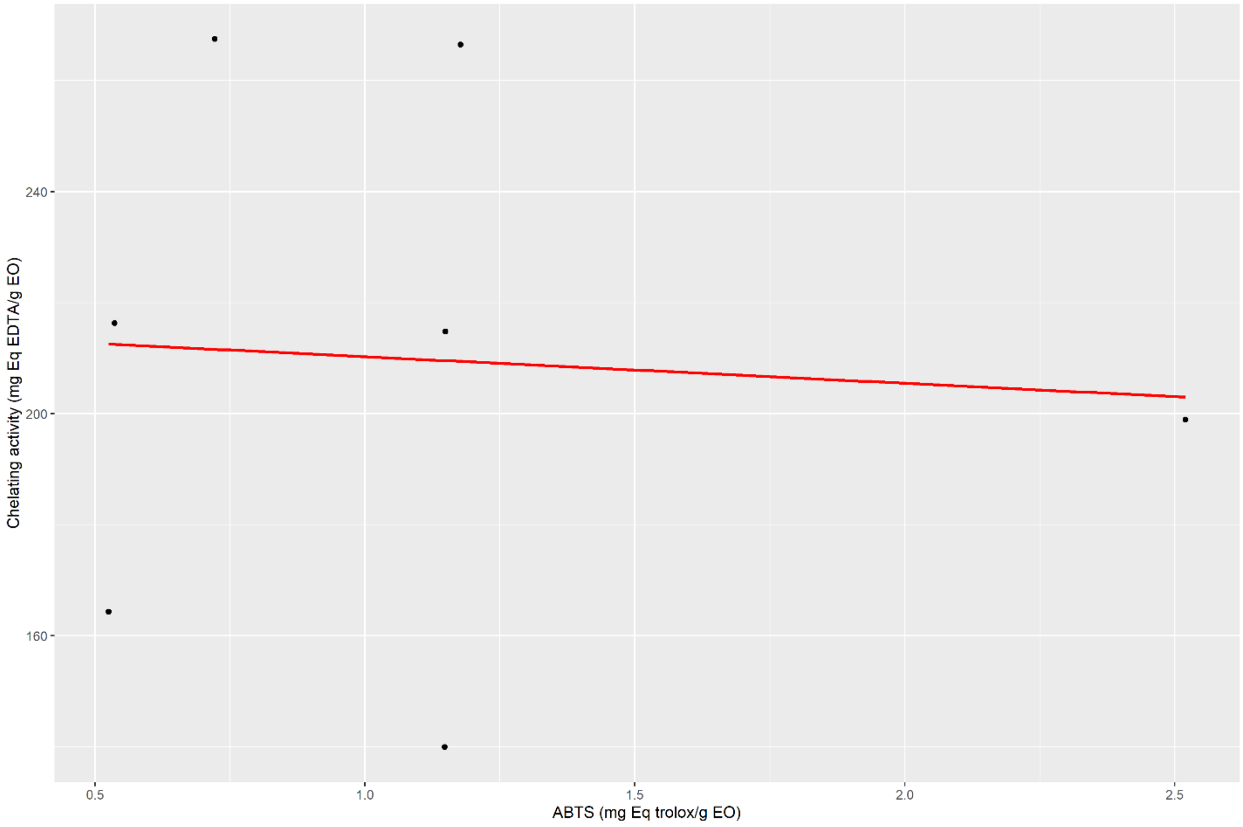

Table S2 and Figure S2. ABTS vs. FRAP (*Pinus pinaster* Aiton, [72])

Antioxidant endpoint	Test
110.42	ABTS
102.24	ABTS
107.28	ABTS
20.69	FRAP
27.92	FRAP
18.31	FRAP

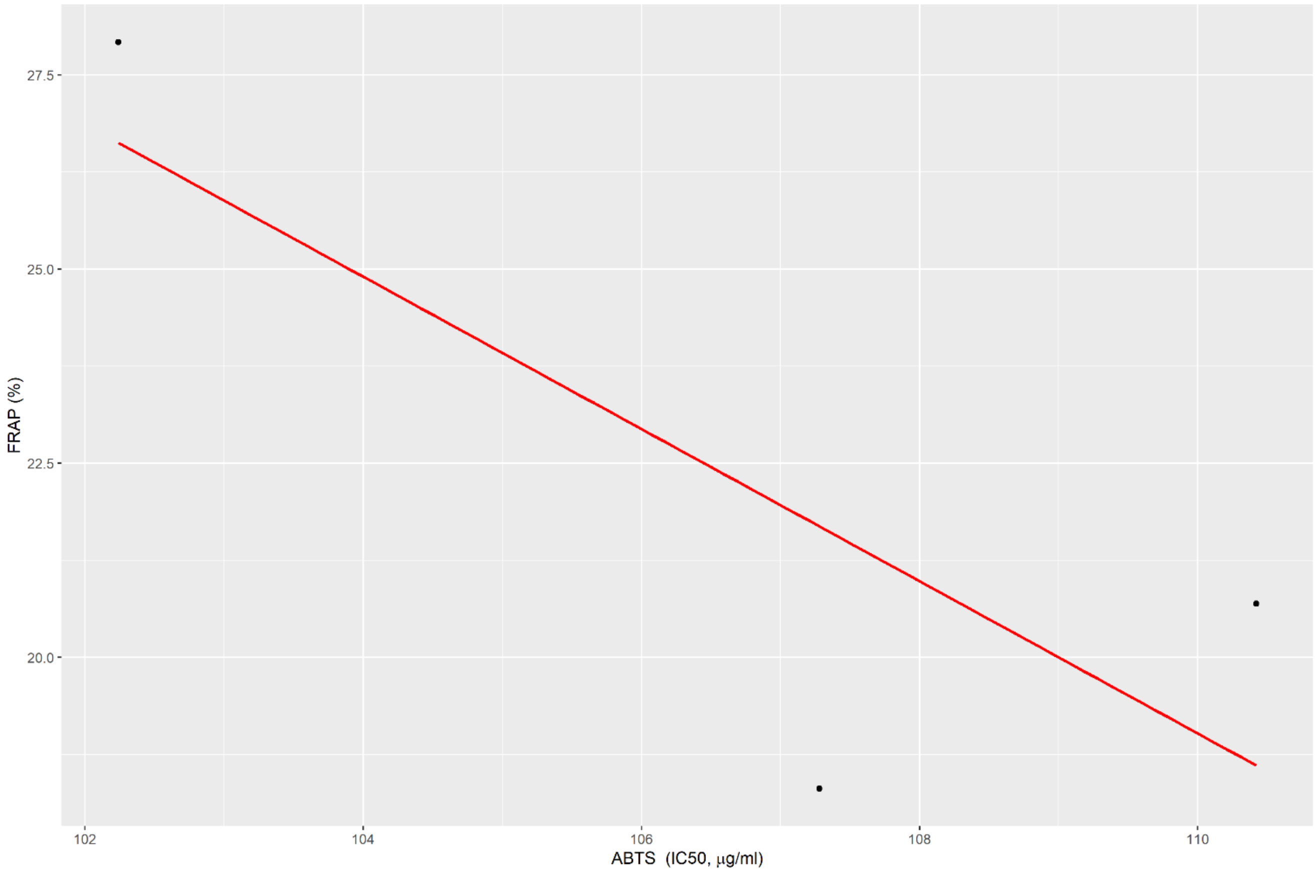

Table S3 and Figure S3. ABTS vs. FRAP (six Pinus taxa, [41])

Antioxidant endpoint	Test
3078.52 ± 278.59	ABTS
2467.85 ± 141.63	ABTS
3584.41 ± 315.63	ABTS
3486.33 ± 140.75	ABTS
2151.43 ± 215.03	ABTS
1461.01 ± 131.03	ABTS
1036.68 ± 51.14	FRAP
904.72 ± 90.73	FRAP
1134.45 ± 36.14	FRAP
814.72 ± 112.41	FRAP
584.78 ± 68.67	FRAP
477.78 ± 48.67	FRAP

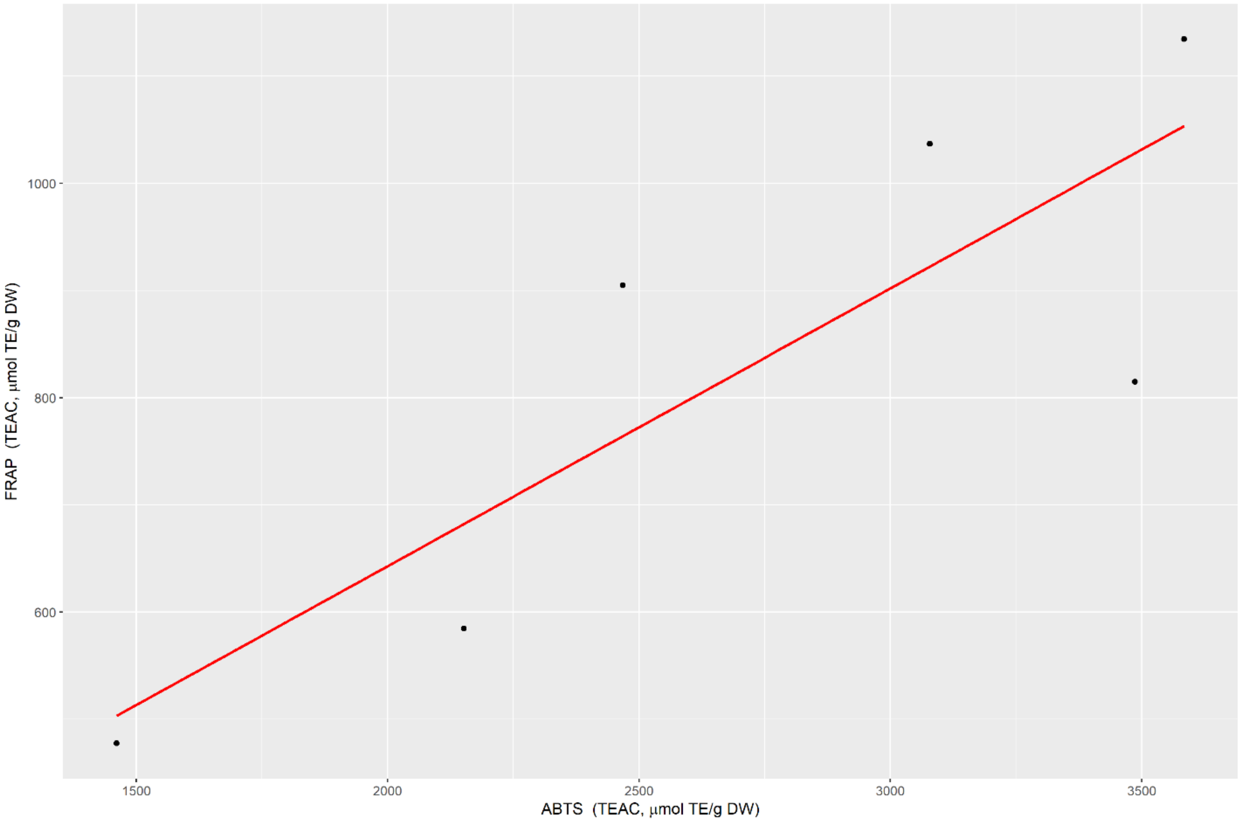

Table S4 and Figure S4. ABTS vs. FRAP (Ten Pinus taxa, [49])

Antioxidant endpoint	Test
1,378.50 ± 137.32	ABTS
1,741.67 ± 130.30	ABTS
1,472.99 ± 53.17	ABTS
3,140.51 ± 89.74	ABTS
2,570.07 ± 109.37	ABTS
1,255.67 ± 74.24	ABTS
2,403.74 ± 37.92	ABTS
3,857.93 ± 92.91	ABTS
2,501.97 ± 51.06	ABTS
1,674.41 ± 15.16	ABTS
441.18 ± 13.00	FRAP
418.63 ± 7.56	FRAP
406.09 ± 3.88	FRAP
1,677.19 ± 104.61	FRAP
1,007.24 ± 83.56	FRAP
370.81 ± 9.78	FRAP
564.64 ± 25.82	FRAP
1,286.11 ± 76.20	FRAP
1,007.62 ± 59.50	FRAP
956.63 ± 55.66	FRAP

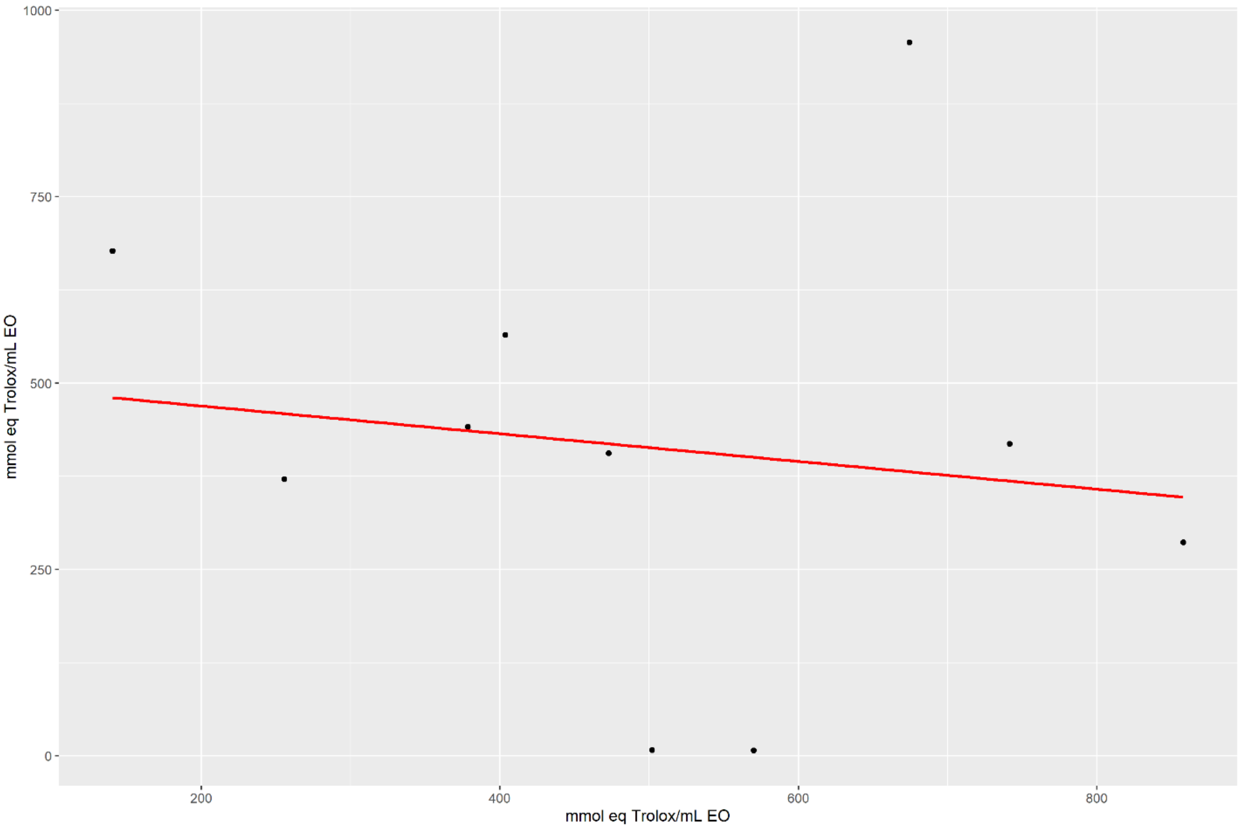

Table S5 and Figure S5. ABTS vs. •OH-radical (Pinus pinaster Aiton, [72])

Antioxidant endpoint	Test
110.42	ABTS
102.24	ABTS
107.28	ABTS
138.25	OH_radical
105.17	OH_radical
152.27	OH_radical

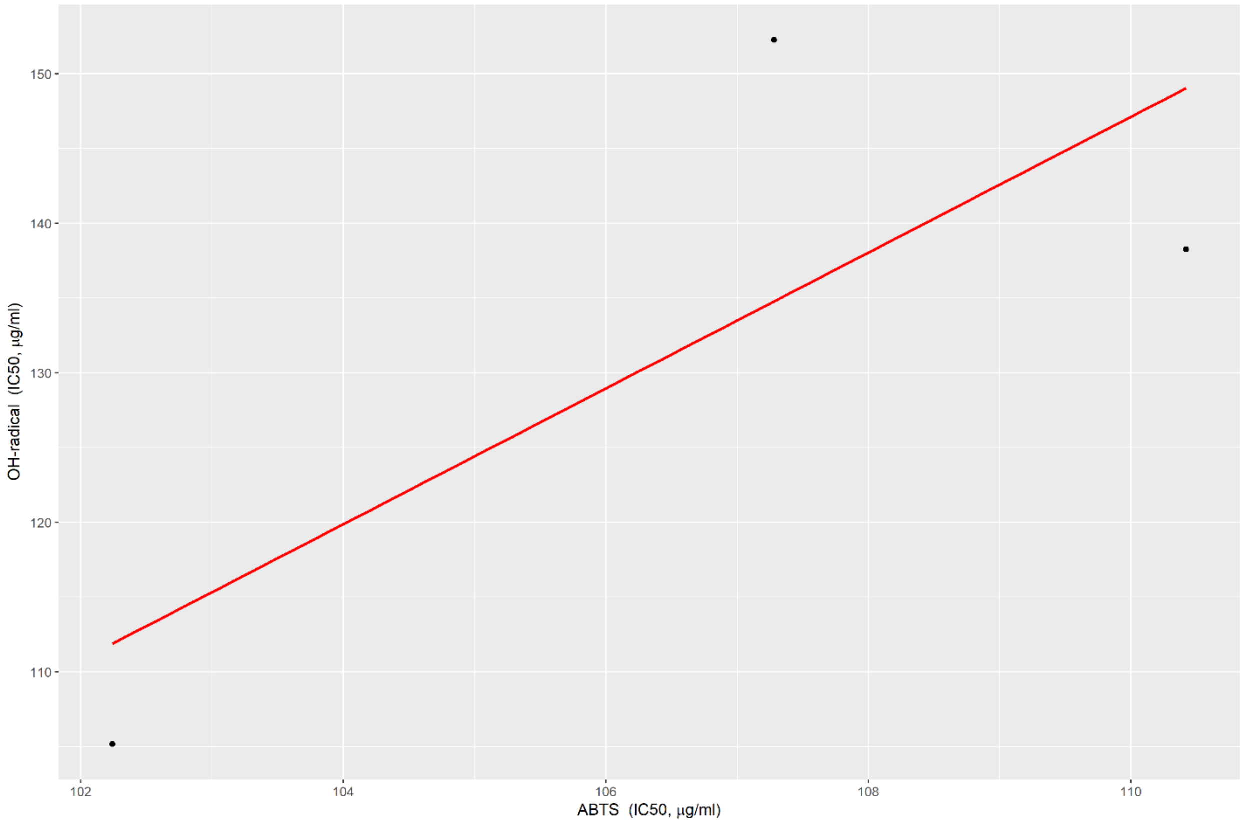

Table S6 and Figure S6. ABTS vs. reducing power (*Pinus halepensis* Mill., [81])

Antioxidant endpoint	Test
577.33 ± 20.52a	ABTS
279.78 ± 2.08 f	ABTS
349.11 ± 10.27e	ABTS
537.36 ± 5.66b	ABTS
212.00 ± 2.38 g	ABTS
337.42 ± 2.79e	ABTS
473.26 ± 5.72 c	ABTS
529.62 ± 15.12b	ABTS
380.25 ± 3.84d	ABTS
197.87 ± 0.22 g	ABTS
517.24 ± 15.07b	ABTS
1.36 ± 0.38 c	Reducing_power
1.64 ± 0.23 c	Reducing_power
1.54 ± 0.27 c	Reducing_power
3.92 ± 0.23a	Reducing_power
3.04 ± 0.30b	Reducing_power
1.41 ± 0.31 c	Reducing_power
1.30 ± 0.20 c	Reducing_power
1.98 ± 0.16 c	Reducing_power
1.51 ± 0.49 c	Reducing_power
3.05 ± 0.30bc	Reducing_power
1.23 ± 0.34 c	Reducing_power

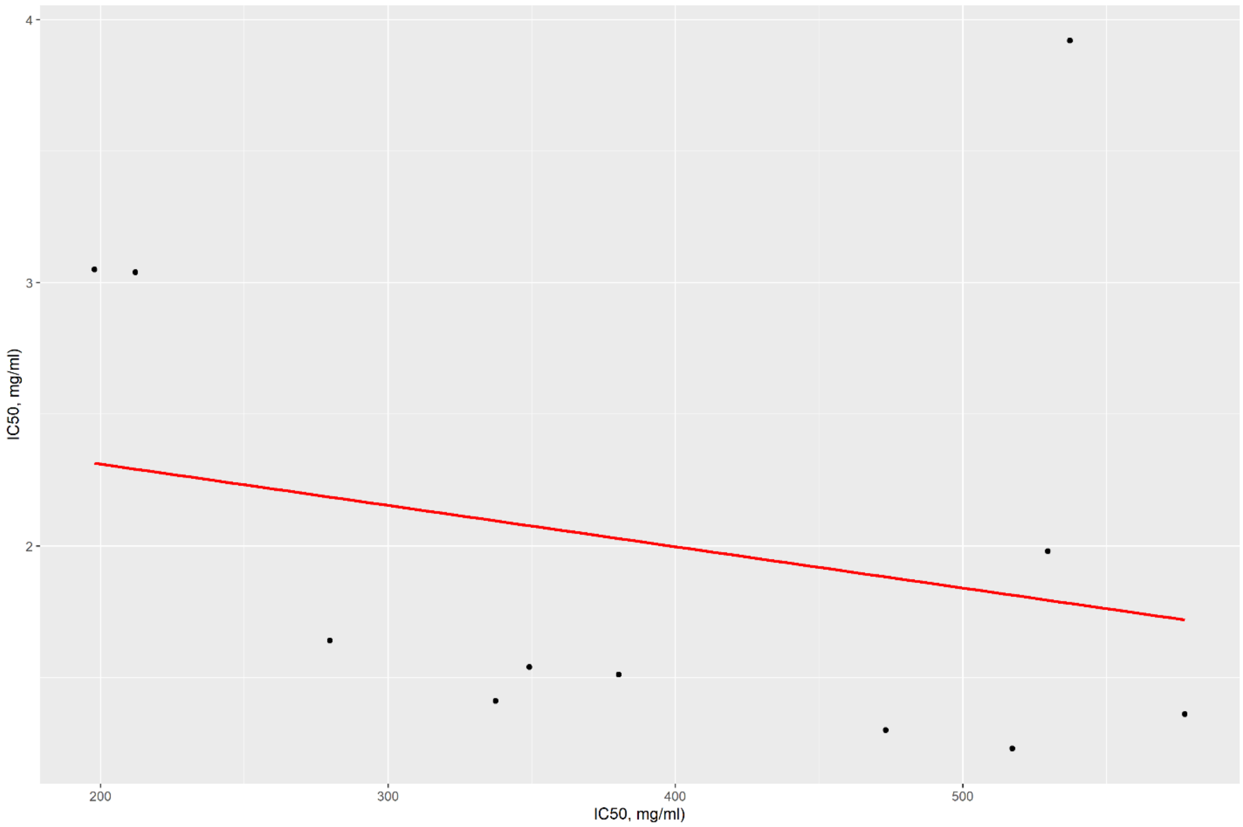

Table S7 and Figure S7. Beta-carotene bleaching vs. Ferrous ion-chelating activity (Pinus halepensis Mill., [69])

ANTIOXIDANT ENDPOINT	Test
52.21932115	Carotene_bleaching
52.21932115	Carotene_bleaching
53.52480418	Carotene_bleaching
49.60835509	Carotene_bleaching
52.21932115	Carotene_bleaching
67.88511749	Carotene_bleaching
71.80156658	Carotene_bleaching
71.80156658	Carotene_bleaching
84.85639687	Carotene_bleaching
96.60574413	Carotene_bleaching
354.1666667	Iron_chelation
352.8645833	Iron_chelation
355.46875	Iron_chelation
212.2395833	Iron_chelation
355.46875	Iron_chelation
389.3229167	Iron_chelation
385.4166667	Iron_chelation
243.4895833	Iron_chelation
425.78125	Iron_chelation
424.4791667	Iron_chelation

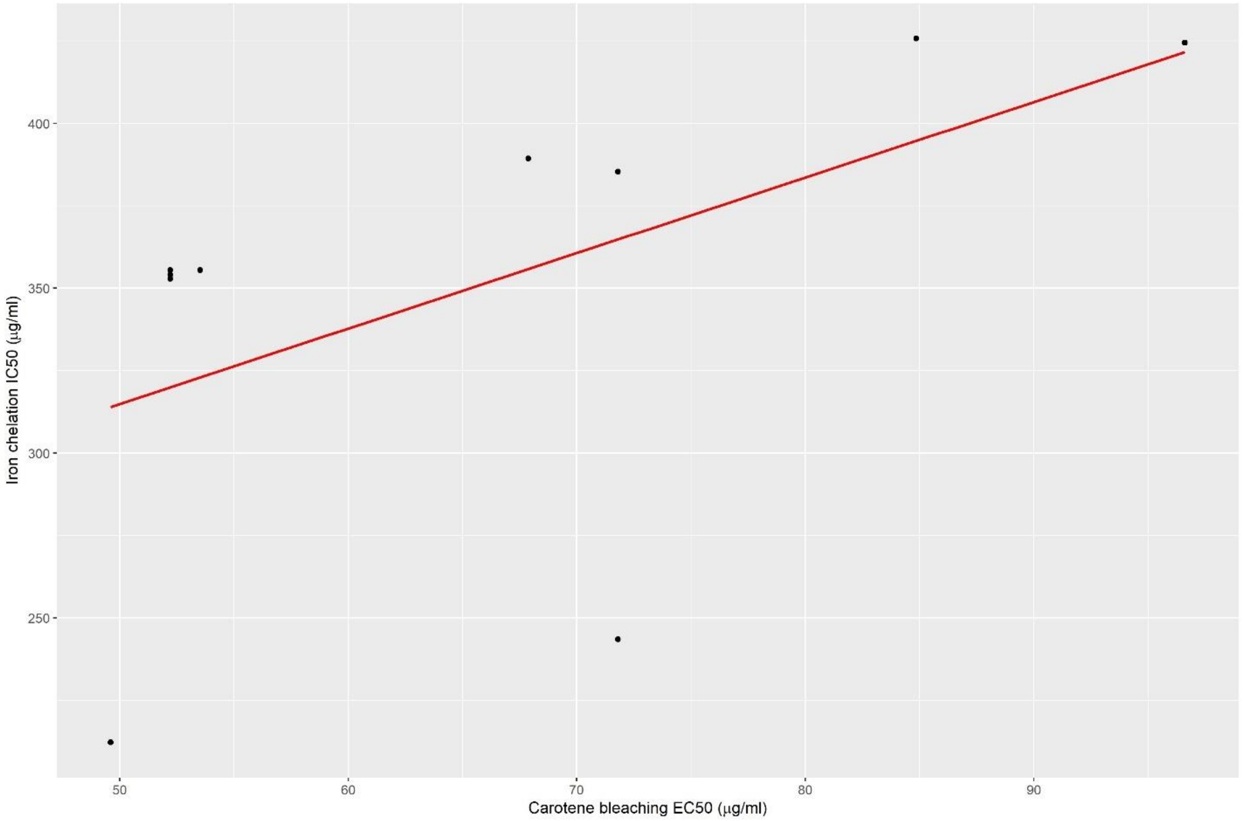

Table S8 and Figure S8. Beta-carotene bleach-ing vs. Nitric oxide radical scavenging (Pinus pinea L., [101])

Antioxidant endpoint	Test
110.5 ± 1.3	Beta_carotene
115.4 ± 2.1	Beta_carotene
138.2 ± 2.3	Beta_carotene
175.0 ± 2.5	Nitric_oxide
185.1 ± 1.9	Nitric_oxide
201.2 ± 1.7	Nitric_oxide

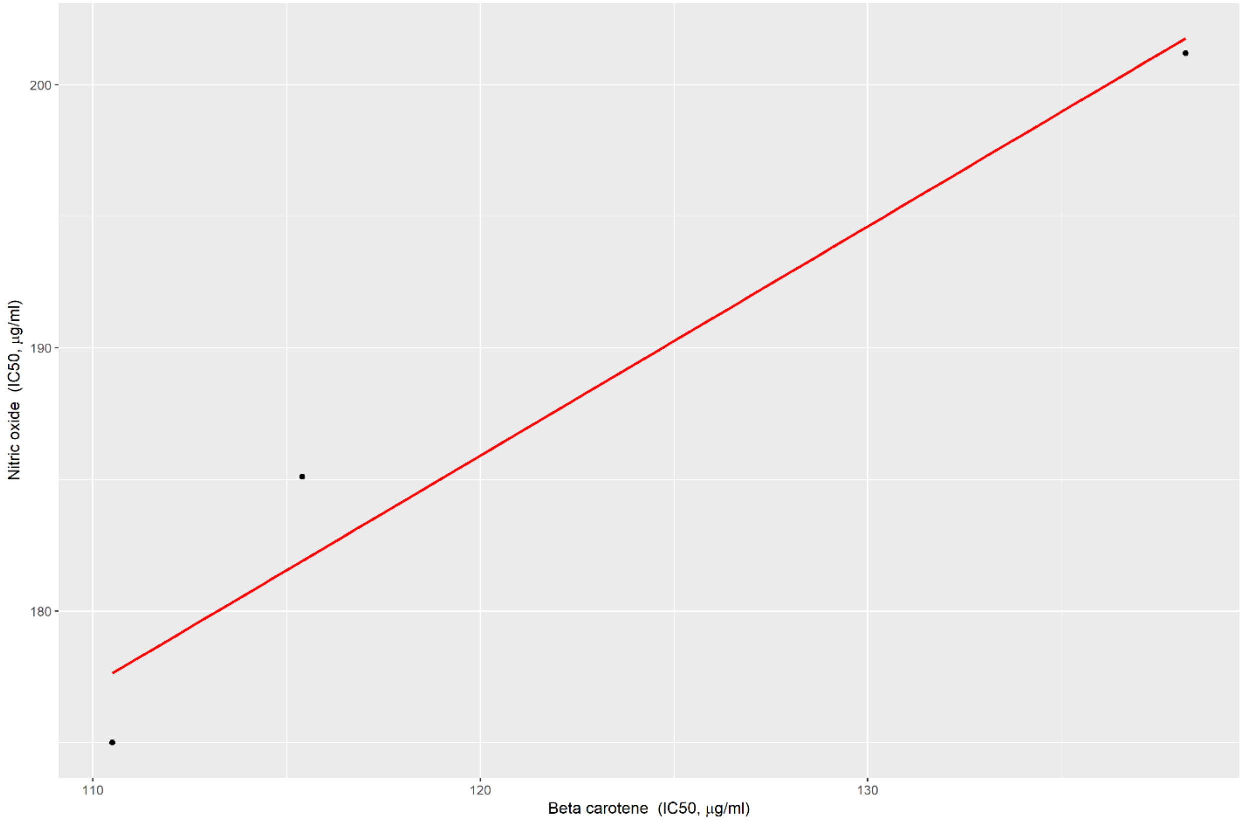

Table S9 and Figure S9. DPPH vs. ABTS (*Pinus halepensis* Mill., [91])

Antioxidant endpoint	Test
0.099 ± 0.004	DPPH
0.312 ± 0.013	DPPH
0.052 ± 0.003	DPPH
0.133 ± 0.001	DPPH
0.016 ± 0.004	DPPH
0.054 ± 0.003	DPPH
0.035 ± 0.006	DPPH
0.722 ± 0.031	ABTS
1.177 ± 0.057	ABTS
1.149 ± 0.013	ABTS
1.148 ± 0.07	ABTS
2.52 ± 0.022	ABTS
0.536 ± 0.036	ABTS
0.525 ± 0.023	ABTS

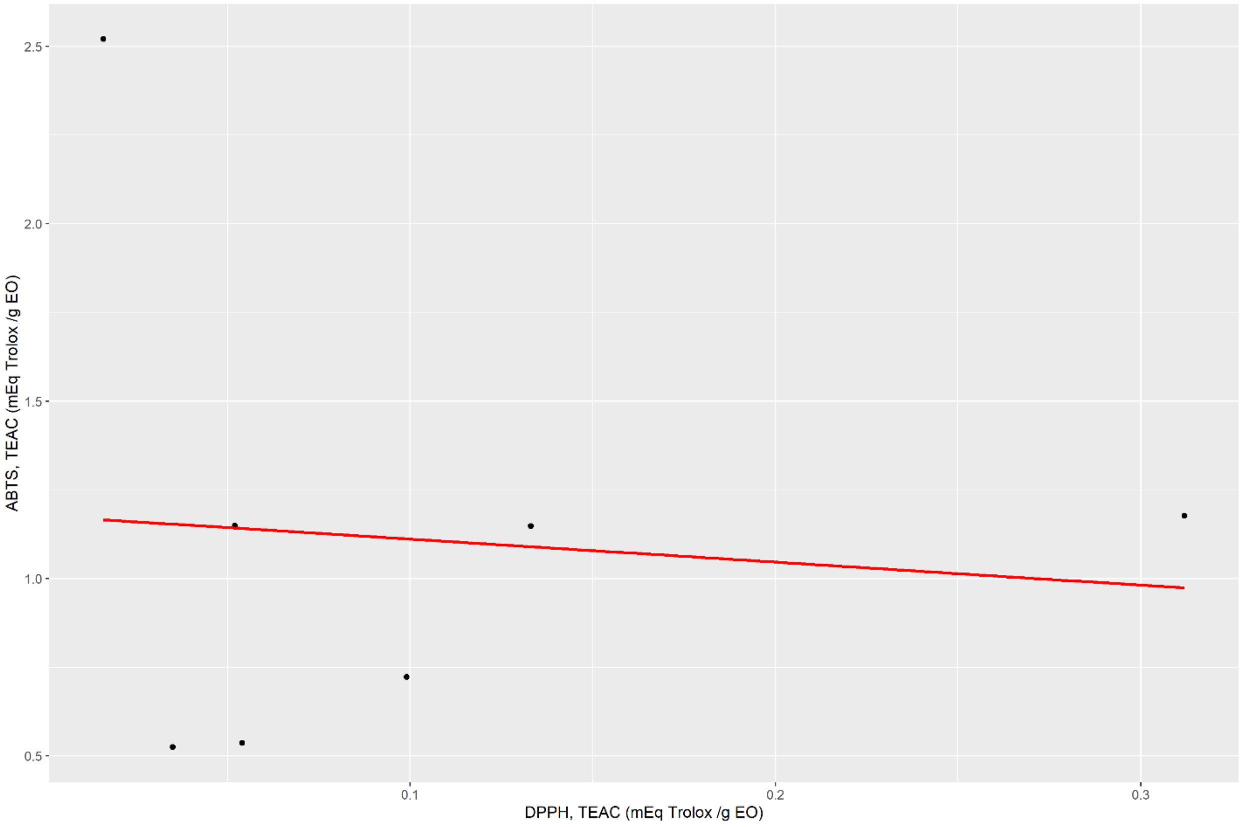

Table S10 and Figure S10. DPPH vs. ABTS (Pinus halepensis Mill., [95])

Antioxidant endpoint	Test
14.62 ± 1.59	DPPH
16.93 ± 1.73	DPPH
18.33 ± 1.97	DPPH
4.59 ± 0.55	ABTS
6.25 ± 0.79	ABTS
7.65 ± 0.88	ABTS

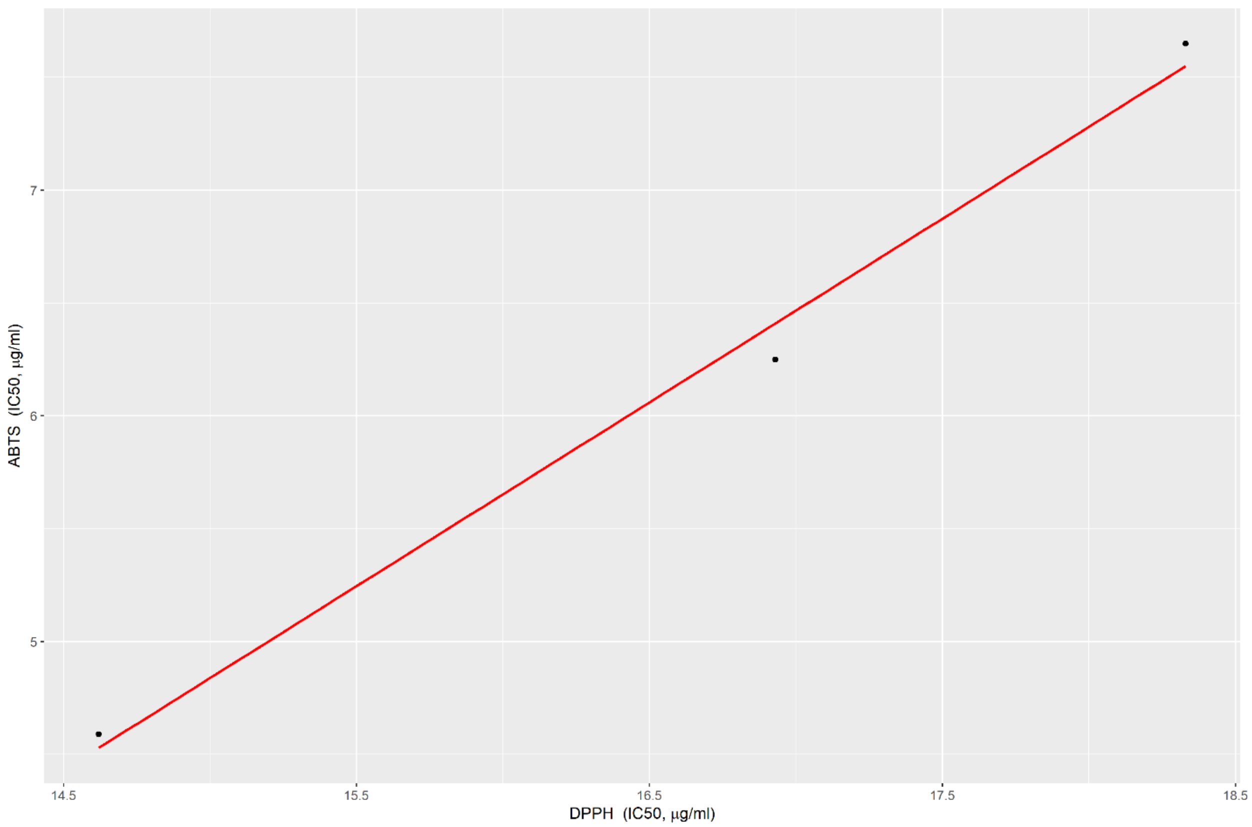

Table S11 and Figure S11. DPPH vs. ABTS (Pinus halepensis Mill., [81])

Antioxidant endpoint	Test
196.01 ± 2.32 c	DPPH
73.03 ± 5.44e	DPPH
114.24 ± 6.34d	DPPH
263.35 ± 13.00a	DPPH
203.10 ± 5.48bc	DPPH
132.69 ± 2.63d	DPPH
251.15 ± 9.26a	DPPH
270.86 ± 8.79a	DPPH
199.17 ± 4.23 c	DPPH
222.79 ± 15.61b	DPPH
191.09 ± 4.51 c	DPPH
577.33 ± 20.52a	ABTS
279.78 ± 2.08 f	ABTS
349.11 ± 10.27e	ABTS
537.36 ± 5.66b	ABTS
212.00 ± 2.38 g	ABTS
337.42 ± 2.79e	ABTS
473.26 ± 5.72 c	ABTS
529.62 ± 15.12b	ABTS
380.25 ± 3.84d	ABTS
197.87 ± 0.22 g	ABTS
517.24 ± 15.07b	ABTS

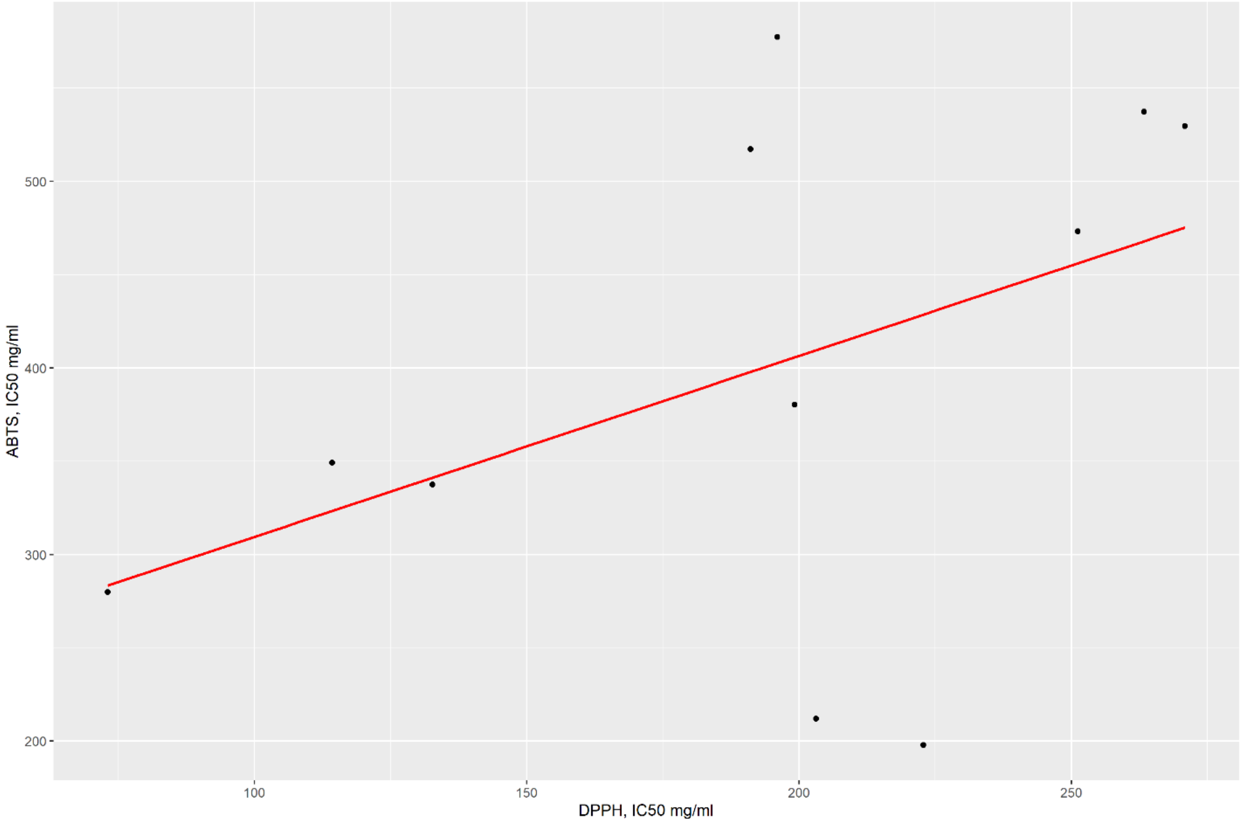

Table S12 and Figure S12. DPPH vs. ABTS (*Pinus pinaster* Aiton, [72])

Antioxidant endpoint	Test
113.45	DPPH
85.82	DPPH
145.8	DPPH
110.42	ABTS
102.24	ABTS
107.28	ABTS

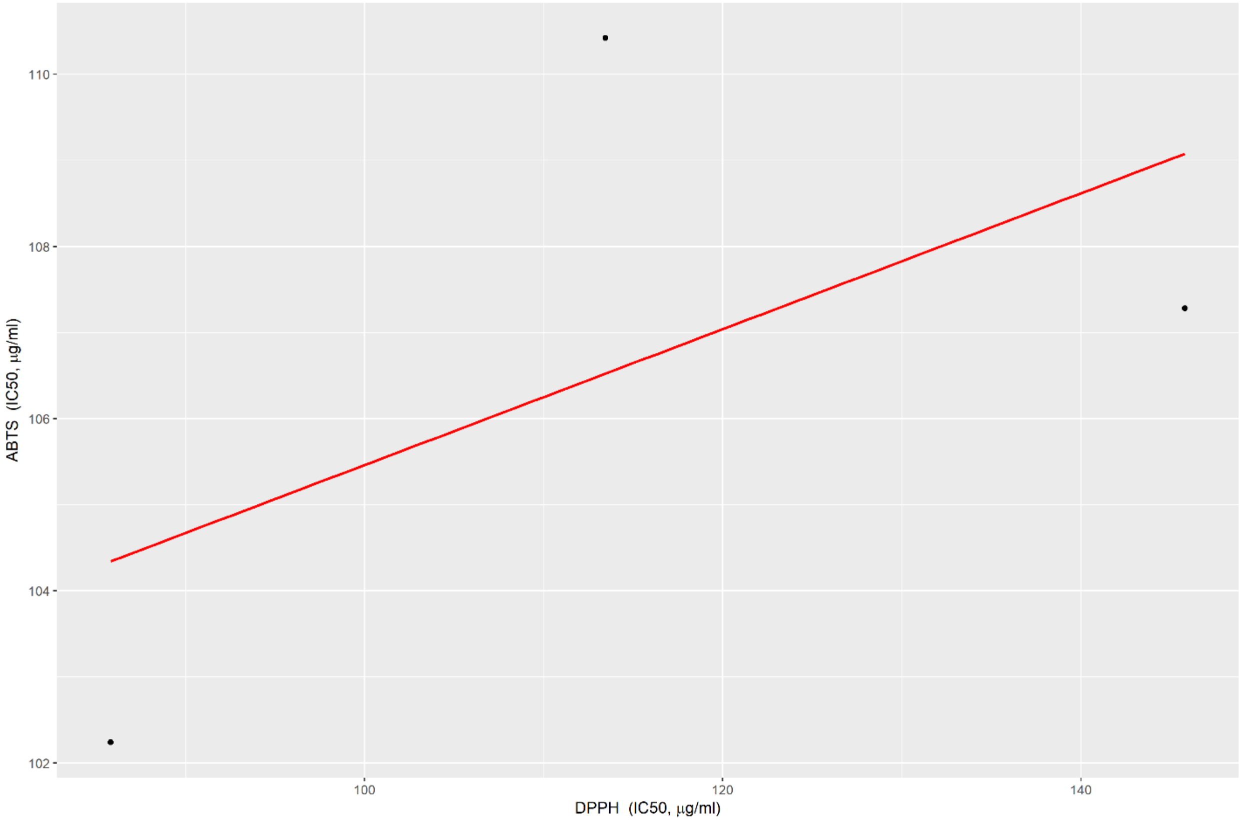

Table S13 and Figure S13. DPPH vs. ABTS (Six *Pinus* taxa, [41])

Antioxidant endpoint	Test
1775.22 ± 138.17	DPPH
1844.19 ± 180.55	DPPH
1851.65 ± 151.19	DPPH
1817.25 ± 131.19	DPPH
918.28 ± 25.37	DPPH
892.45 ± 78.31	DPPH
3078.52 ± 278.59	ABTS
2467.85 ± 141.63	ABTS
3584.41 ± 315.63	ABTS
3486.33 ± 140.75	ABTS
2151.43 ± 215.03	ABTS
1461.01 ± 131.03	ABTS

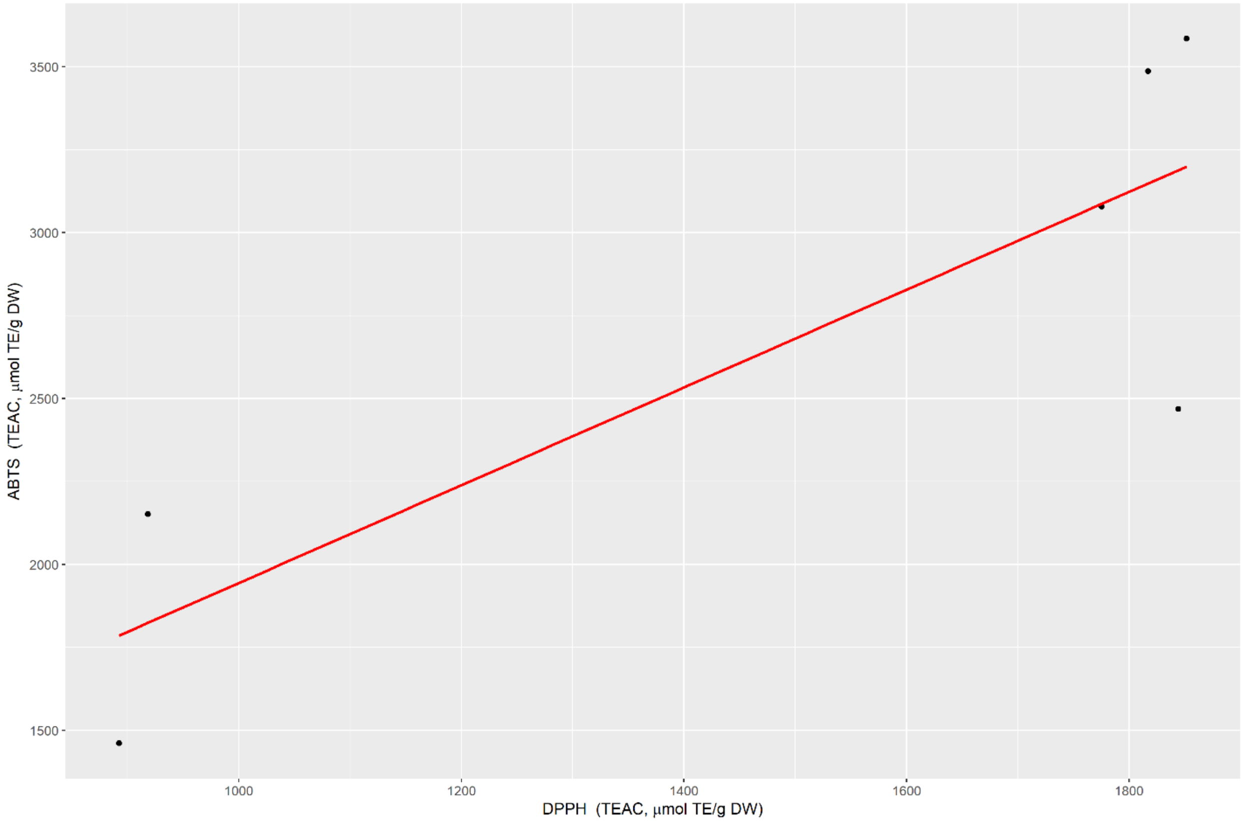

Table S14 and Figure S14. DPPH vs. ABTS (Ten *Pinus* taxa, [49])

Antioxidant endpoint	Test
1,065.00 ± 47.98	DPPH
1,161.66 ± 56.06	DPPH
990.31 ± 53.13	DPPH
1,272.75 ± 21.48	DPPH
1,141.79 ± 73.53	DPPH
499.15 ± 56.76	DPPH
1,155.45 ± 38.97	DPPH
1,263.20 ± 71.51	DPPH
1,128.44 ± 12.61	DPPH
1,118.03 ± 50.73	DPPH
1,378.50 ± 137.32	ABTS
1,741.67 ± 130.30	ABTS
1,472.99 ± 53.17	ABTS
3,140.51 ± 89.74	ABTS
2,570.07 ± 109.37	ABTS
1,255.67 ± 74.24	ABTS
2,403.74 ± 37.92	ABTS
3,857.93 ± 92.91	ABTS
2,501.97 ± 51.06	ABTS
1,674.41 ± 15.16	ABTS

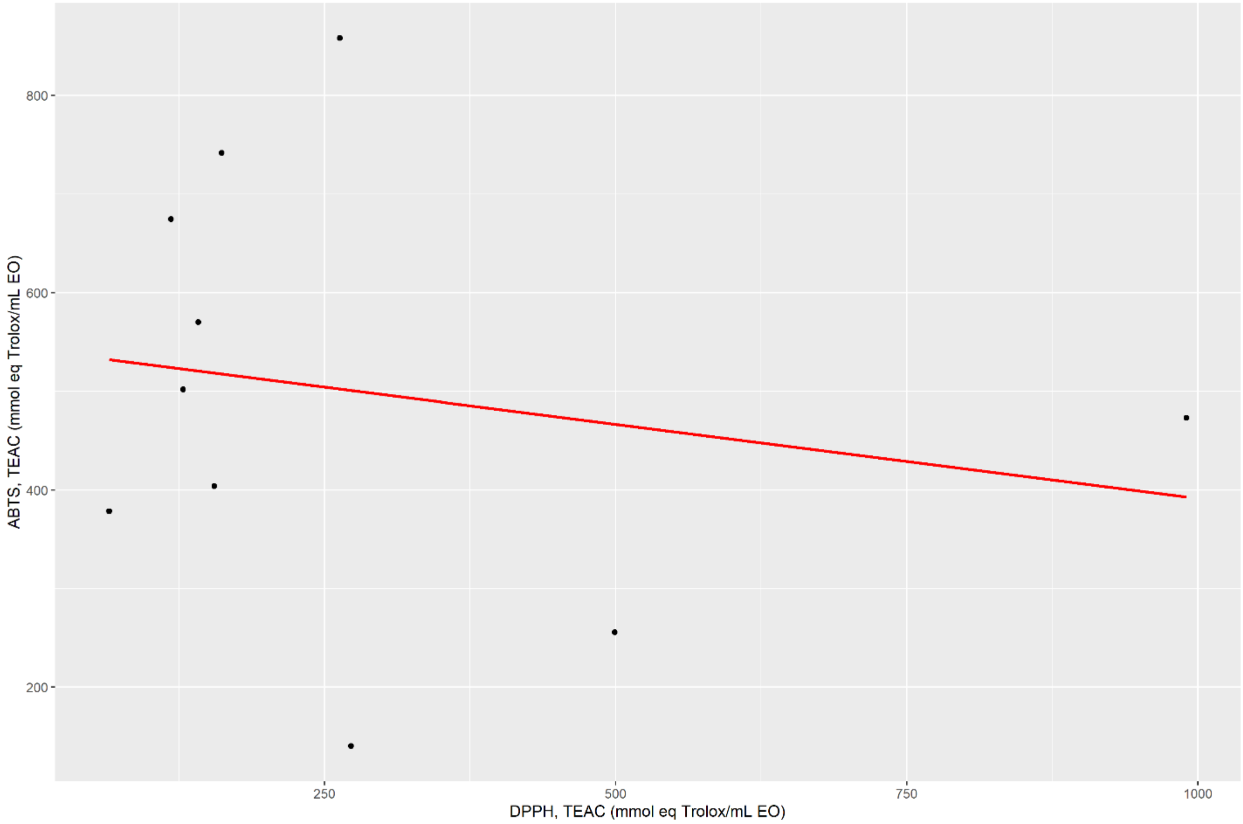

Table S15 and Figure S15. DPPH vs. ABTS (Pinus cembra L., Pinus mugo Turra, Picea abies L., and Abies alba Mill., [40])

Antioxidant endpoint	Test
13.01 ± 0.86	DPPH
3.08 ± 0.65	DPPH
13.05 ± 3.09	DPPH
7.84 ± 1.70	DPPH
44.90 ± 2.06	ABTS
43.08 ±6.95	ABTS
45.00 ± 6.26	ABTS
44.23 ± 1.10	ABTS

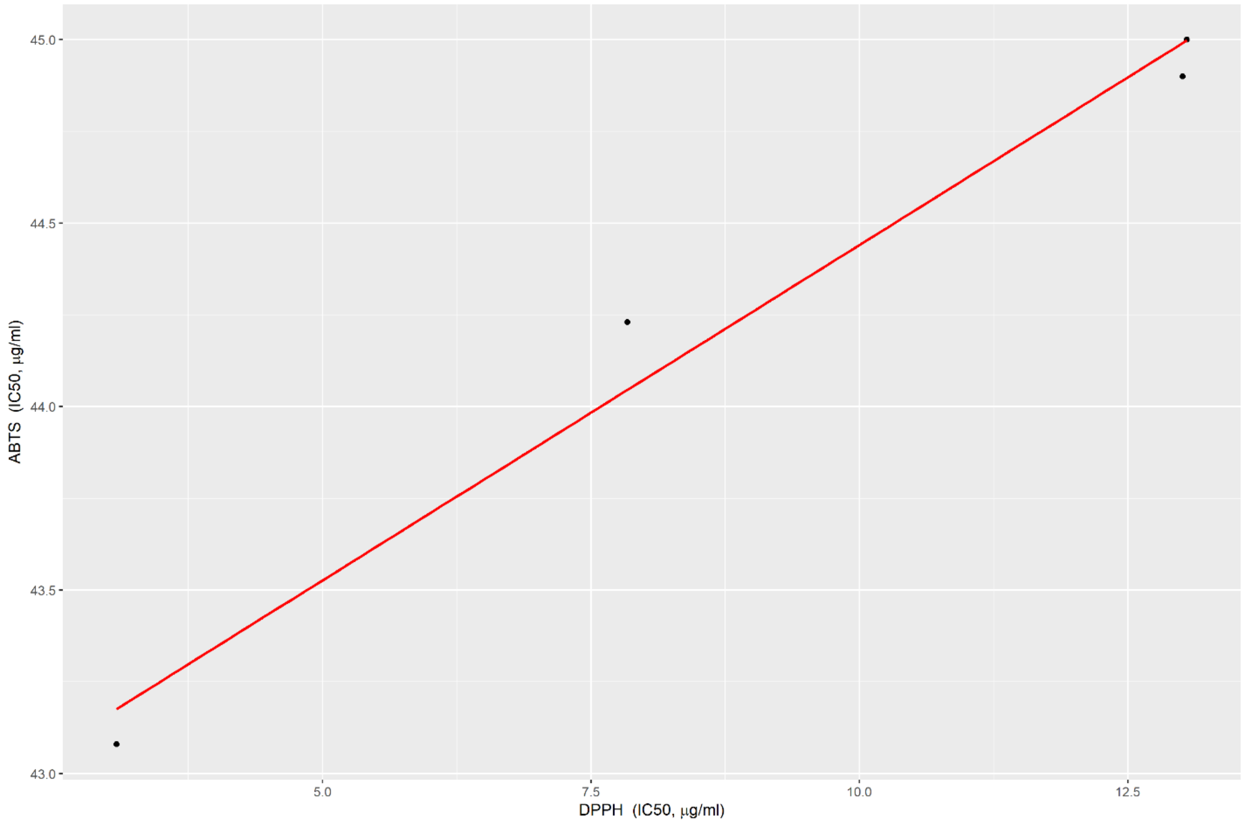

Table S16 and Figure S16. DPPH vs. ABTS (TEAC) (Pinus cembra L., Pinus mugo Turra, Picea abies L., and Abies alba Mill., [40])

Antioxidant endpoint	Test
1.63 ± 0.46	DPPH
7.65 ± 1.33	DPPH
1.68 ± 0.64	DPPH
3.01 ± 0.48	DPPH
13.26 ± 1.45	ABTS
14.01 ± 2.01	ABTS
13.26 ± 0.52	ABTS
13.65 ± 0.49	ABTS

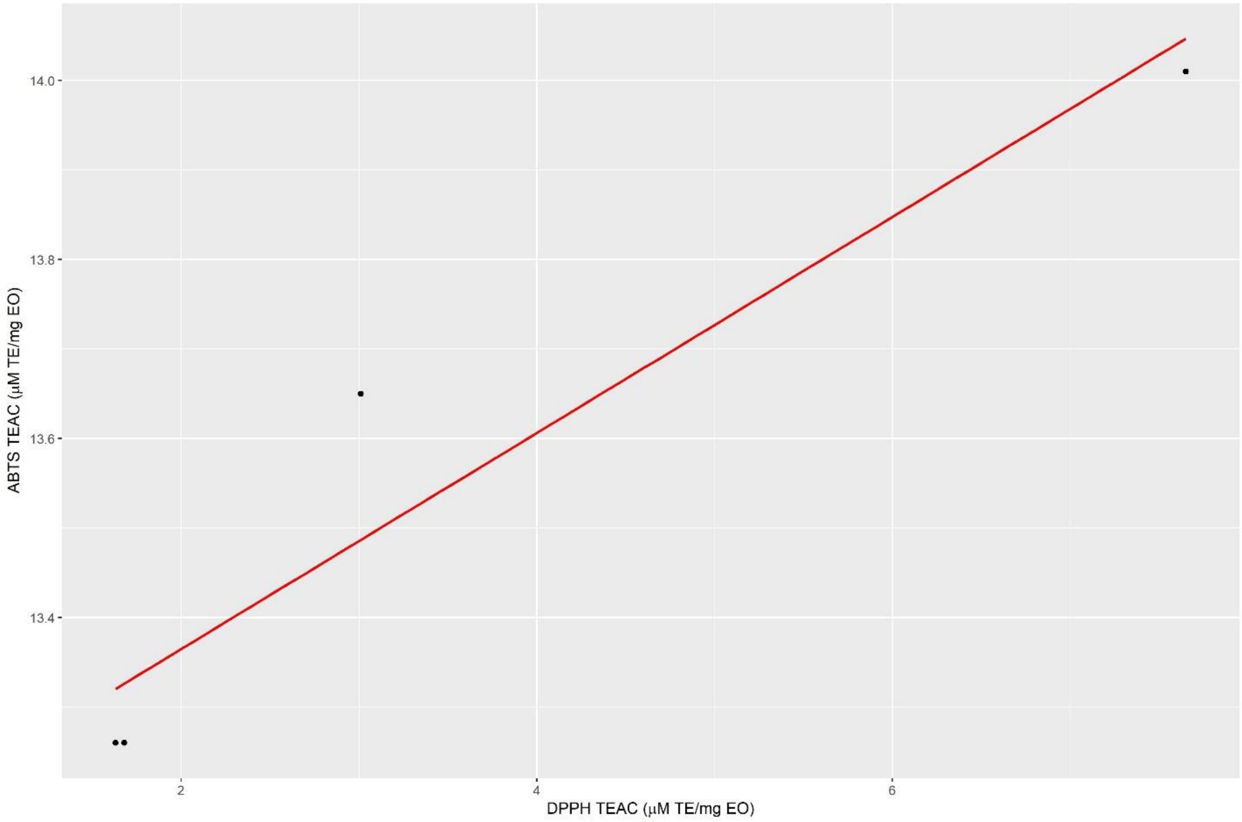

Table S17 and Figure S17. DPPH vs. Beta-carotene bleaching (*Pinus halepensis* Mill., [69])

ANTIOXIDANT ENDPOINT	Test
215.5172414	DPPH
212.6436782	DPPH
214.0804598	DPPH
213.71	DPPH
212.96	DPPH
238.5057471	DPPH
239.9425287	DPPH
244.2528736	DPPH
278.7356322	DPPH
284.12	DPPH
52.21932115	Carotene_bleaching
52.21932115	Carotene_bleaching
53.52480418	Carotene_bleaching
49.60835509	Carotene_bleaching
52.21932115	Carotene_bleaching
67.88511749	Carotene_bleaching
71.80156658	Carotene_bleaching
71.80156658	Carotene_bleaching
84.85639687	Carotene_bleaching
96.60574413	Carotene_bleaching

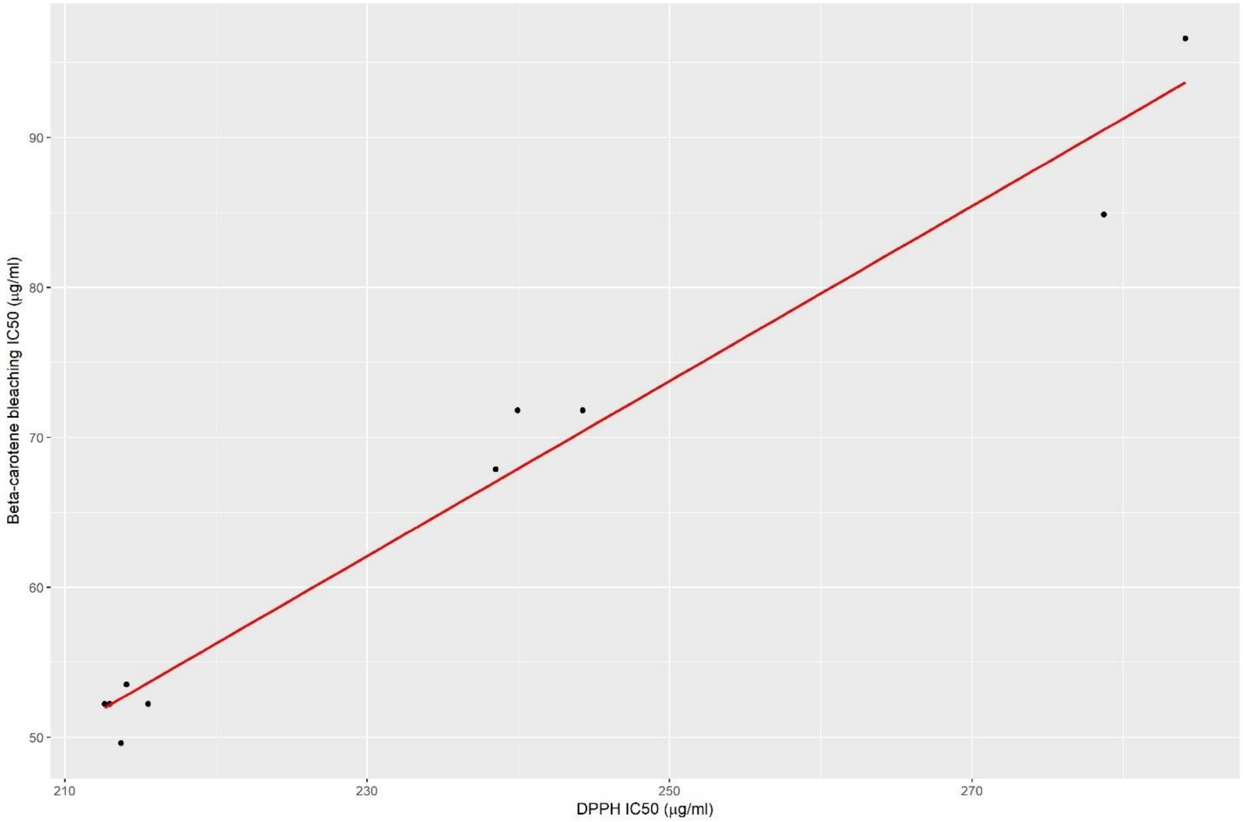

Table S18 and Figure S18. DPPH vs. beta-carotene bleaching assay (*Pinus pinea* L., [101])

Antioxidant endpoint	Test
45.1 ± 1.5	DPPH
40.5 ± 0.7	DPPH
48.4 ± 1.2	DPPH
110.5 ± 1.3	Carotene_bleaching
115.4 ± 2.1	Carotene_bleaching
138.2 ± 2.3	Carotene_bleaching

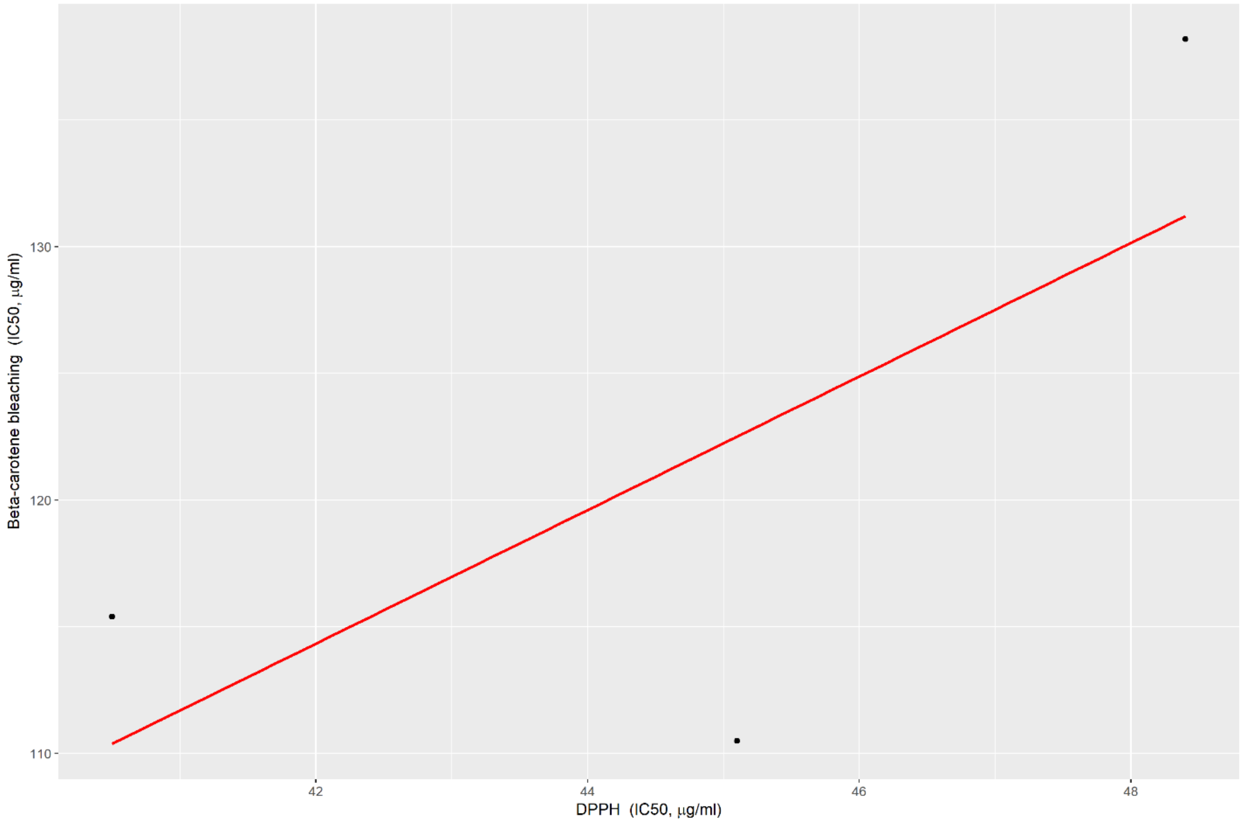

Table S19 and Figure S19. DPPH vs. Ferrous ion-chelating activity (*Pinus halepensis* Mill., [91])

Antioxidant endpoint	Test
0.099 ± 0.004	DPPH
0.312 ± 0.013	DPPH
0.052 ± 0.003	DPPH
0.133 ± 0.001	DPPH
0.016 ± 0.004	DPPH
0.054 ± 0.003	DPPH
0.035 ± 0.006	DPPH
267.434 ± 2.36	Iron_chelation
266.437 ± 1.04	Iron_chelation
214.804 ± 0.99	Iron_chelation
139.985 ± 0.8	Iron_chelation
198.912 ± 1.56	Iron_chelation
216.292 ± 0.33	Iron_chelation
164.319 ± 2.71	Iron_chelation

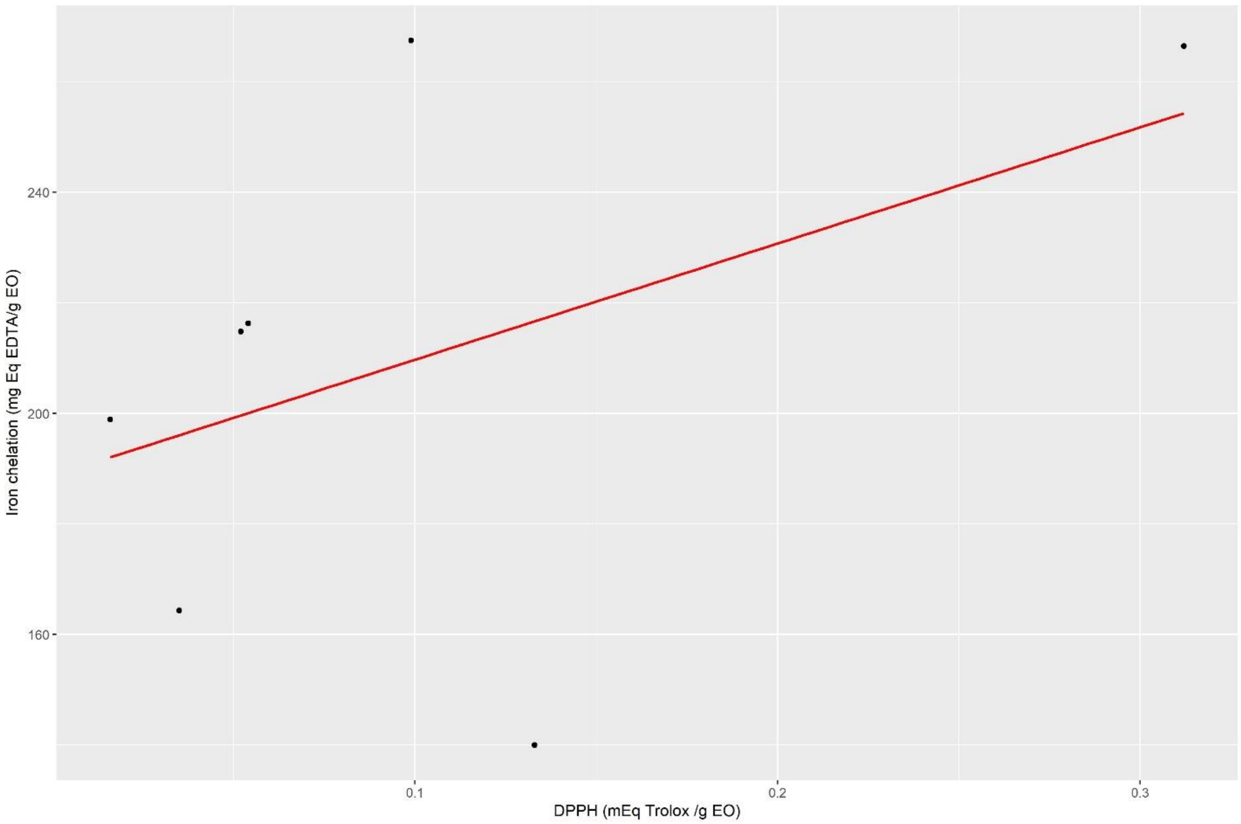

Table S20 and Figure S20. DPPH vs. Ferrous ion-chelating activity (*Pinus halepensis* Mill., [69])

215.5172414	DPPH
212.6436782	DPPH
214.0804598	DPPH
213.71	DPPH
212.96	DPPH
238.5057471	DPPH
239.9425287	DPPH
244.2528736	DPPH
278.7356322	DPPH
284.12	DPPH
354.1666667	Iron_chelation
352.8645833	Iron_chelation
355.46875	Iron_chelation
212.2395833	Iron_chelation
355.46875	Iron_chelation
389.3229167	Iron_chelation
385.4166667	Iron_chelation
243.4895833	Iron_chelation
425.78125	Iron_chelation
424.4791667	Iron_chelation

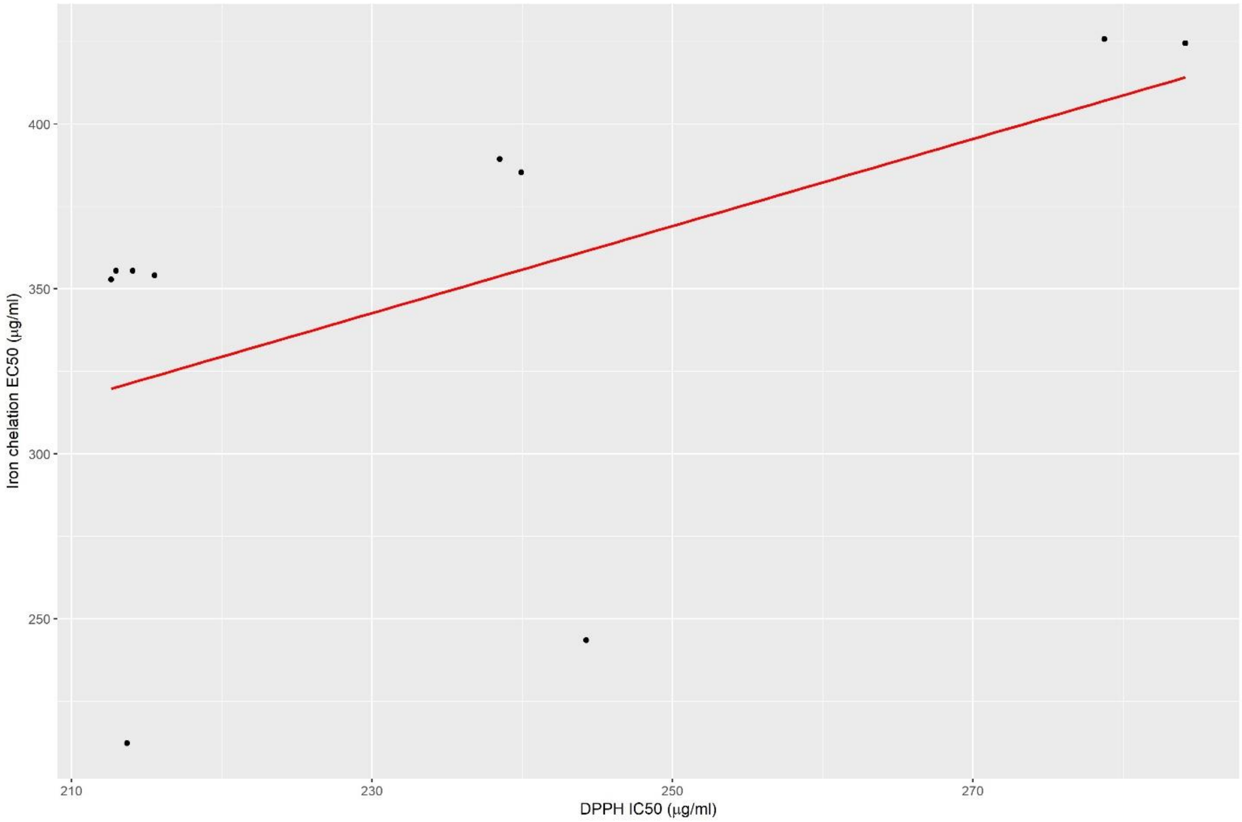

Table S21 and Figure S21. DPPH vs. ferrous ion-chelating activity (*Pinus pinea* L., [101])

Antioxidant endpoint	Test
45.1 ± 1.5	DPPH
40.5 ± 0.7	DPPH
48.4 ± 1.2	DPPH
51.1 ± 0.9	Iron_chelation
48.0 ± 1.1	Iron_chelation
55.0 ± 0.5	Iron_chelation

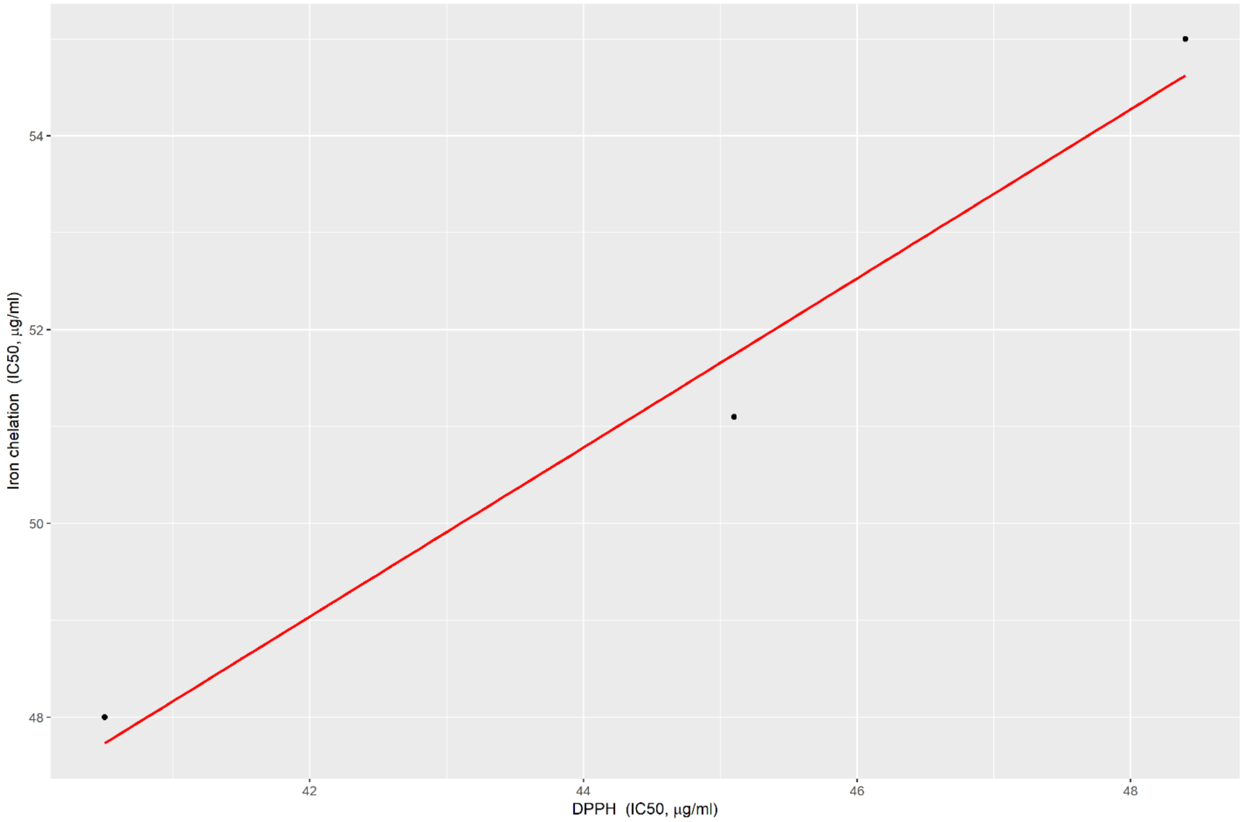

Table S22 and Figure S22. DPPH vs. Folin-Ciocâlteu (TEAC vs. GAE) (*Abies sachalinensis,* [44])

Antioxidant endpoint	Test
489 (control)	DPPH
518.1 (SW3)	DPPH
718.3 (SW7)	DPPH
522.3 (SW10)	DPPH
0.37 (control)	Folin
0.44 (SW3)	Folin
0.99 (SW7)	Folin
0.55 (SW10)	Folin

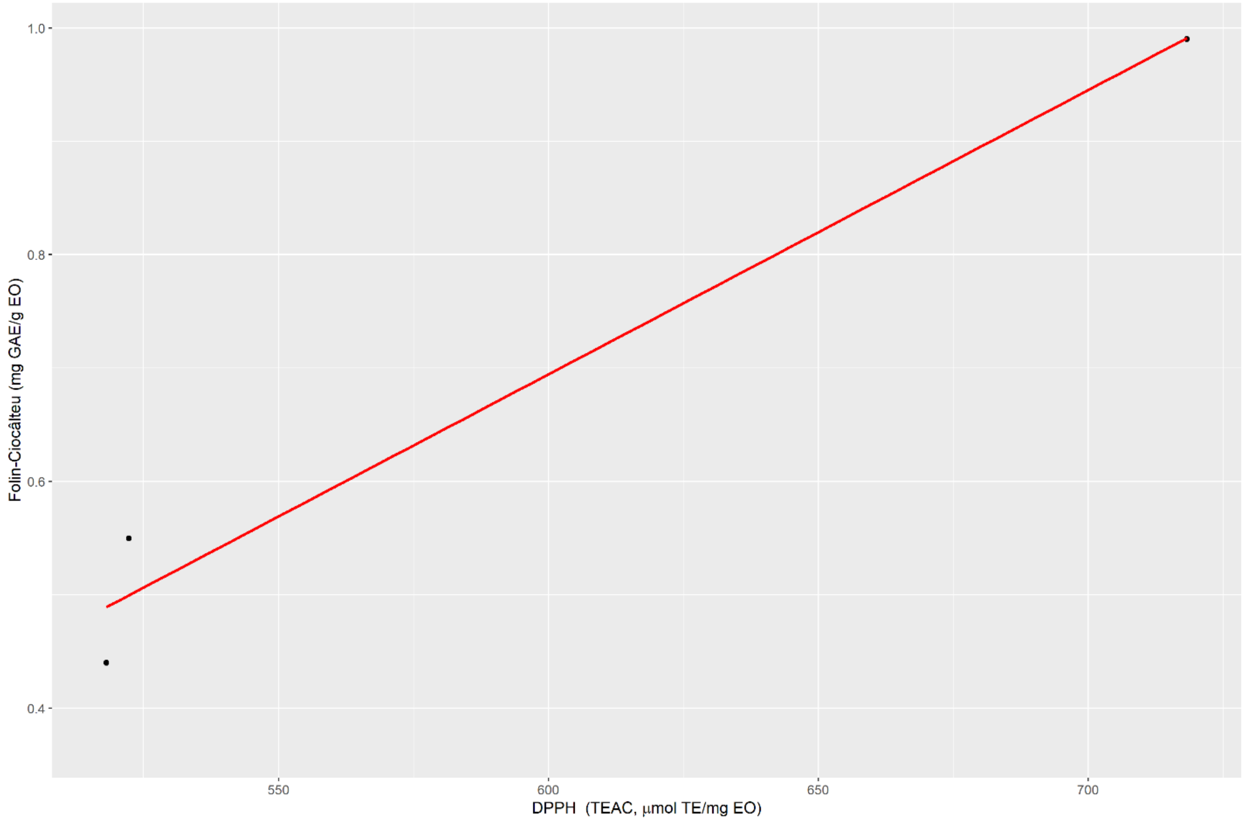

Table S23 and Figure S23. DPPH vs. FRAP (*Cedrus atlantica* (Endl.) G.Manetti ex Carrière, [100])

Antioxidant endpoint	Test
0.126 ± 0.013	DPPH
0.143 ± 0.014	DPPH
15.559 ± 0.715	DPPH
16.264 ± 0.285	DPPH
0.832 ± 0.002	FRAP
0.410 ± 0.002	FRAP
2.219 ± 0.001	FRAP
1.996 ± 0.013	FRAP

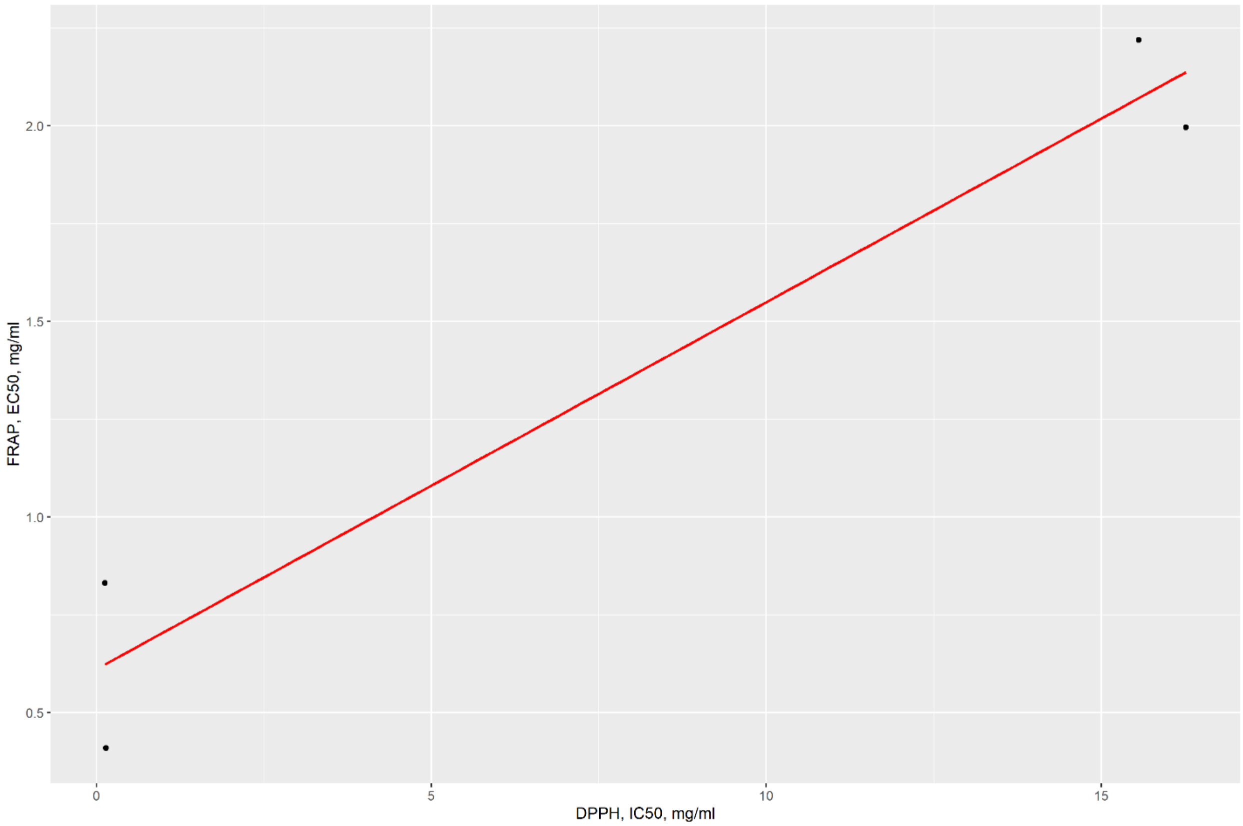

Table S24 and Figure S24. DPPH vs. FRAP (*Pinus pinaster* Aiton, [72])

Antioxidant endpoint	Test
113.45	DPPH
85.82	DPPH
145.8	DPPH
20.69	FRAP
27.92	FRAP
18.31	FRAP

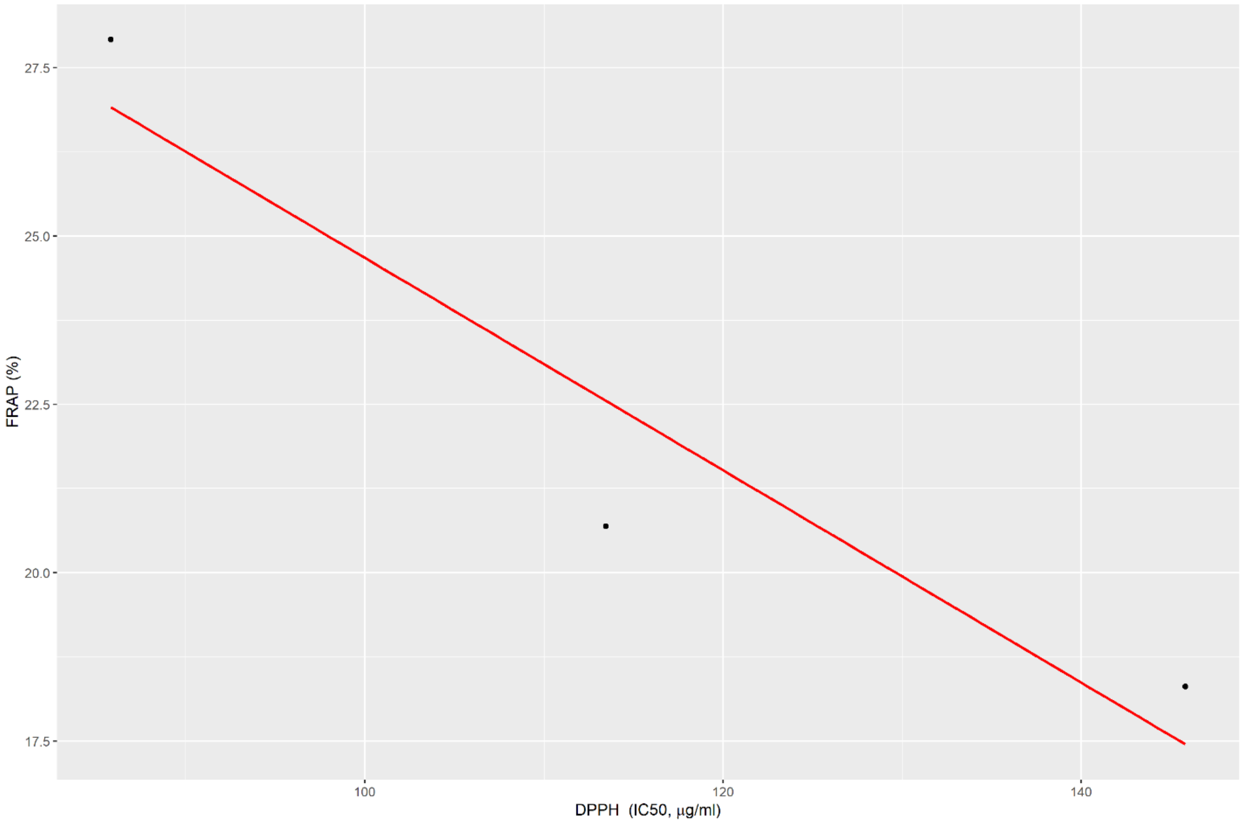

Table S25 and Figure S25. DPPH vs. FRAP (*Pinus pinaster* Aiton, [98])

Antioxidant endpoint	Test
123.0 ± 5.4	DPPH
115.2 ± 4.8	DPPH
59.8 ± 2.1	DPPH
15.0 ± 0.9	DPPH
15.4 ± 1	DPPH
0.307	FRAP
0.322	FRAP
0.401	FRAP
0.598	FRAP
0.591	FRAP

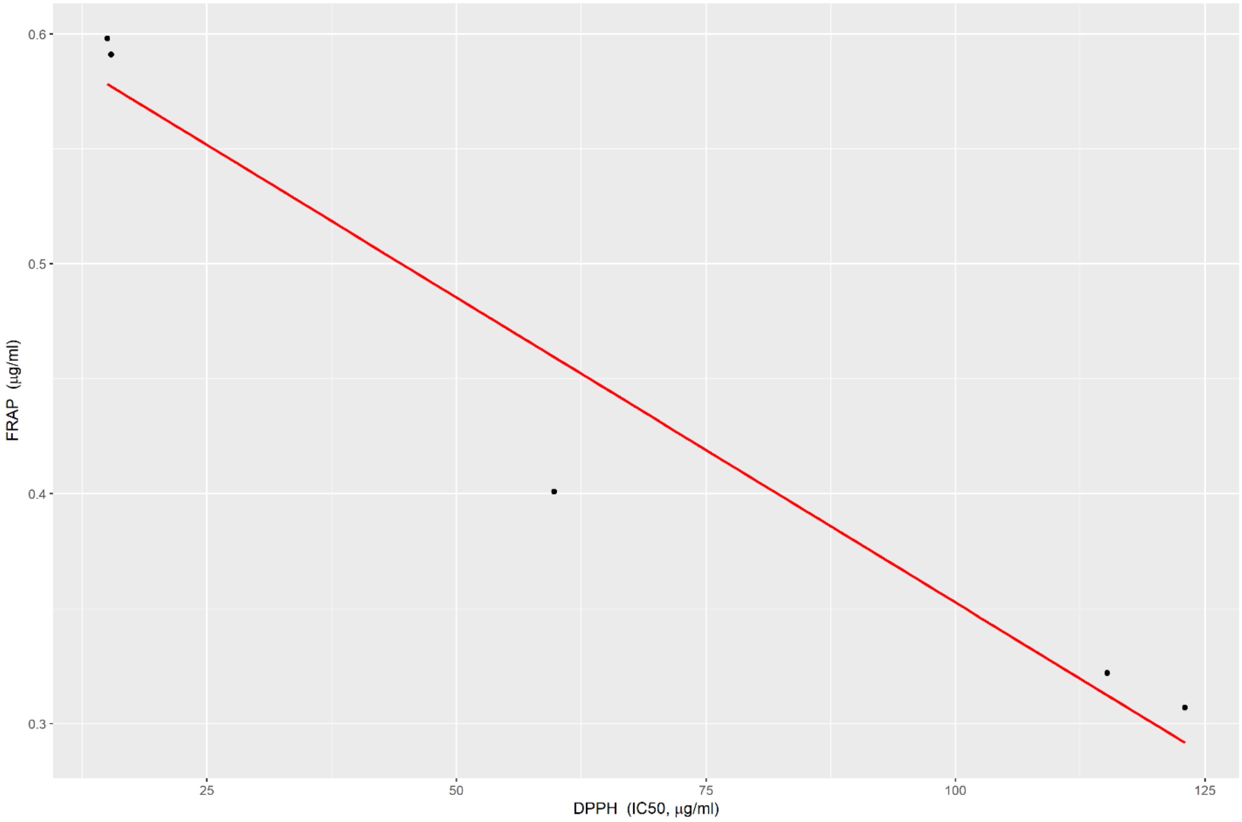

Table S26 and Figure S26. DPPH vs. FRAP (*Pinus roxburghii* Sarg., [62])

Antioxidant endpoint	Test
62.4 ± 0.41	DPPH
49.53 ± 0.11	DPPH
54.67 ± 0.38	DPPH
60.68 ± 0.34	DPPH
63.33 ± 0.47	DPPH
67.44 ± 0.95	FRAP
99.92 ± 1.06	FRAP
102.07 ± 1.12	FRAP
127.12 ± 1.21	FRAP
134.49 ± 1.34	FRAP

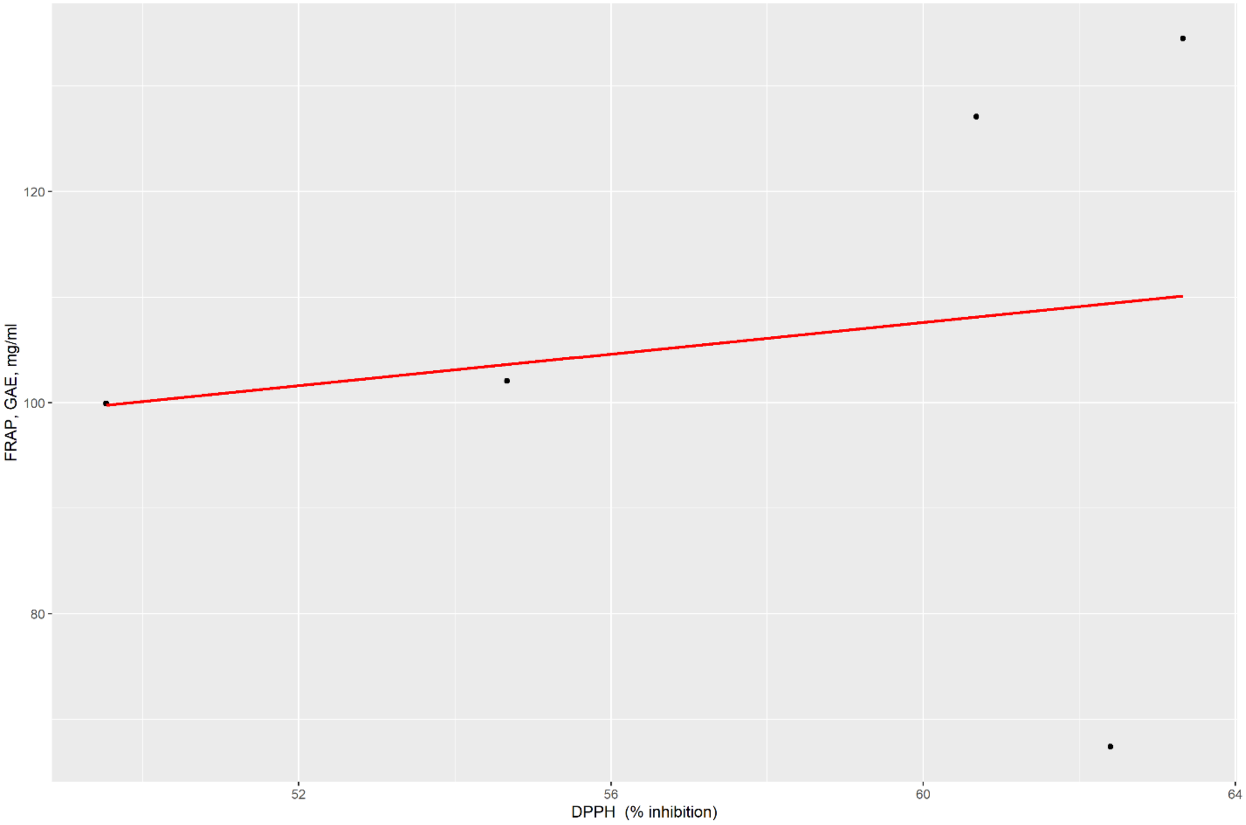

Table S27 and Figure S27. DPPH vs. FRAP (Six *Pinus* taxa, [41])

Antioxidant endpoint	Test
1775.22 ± 138.17	DPPH
1844.19 ± 180.55	DPPH
1851.65 ± 151.19	DPPH
1817.25 ± 131.19	DPPH
918.28 ± 25.37	DPPH
892.45 ± 78.31	DPPH
1036.68 ± 51.14	FRAP
904.72 ± 90.73	FRAP
1134.45 ± 36.14	FRAP
814.72 ± 112.41	FRAP
584.78 ± 68.67	FRAP
477.78 ± 48.67	FRAP

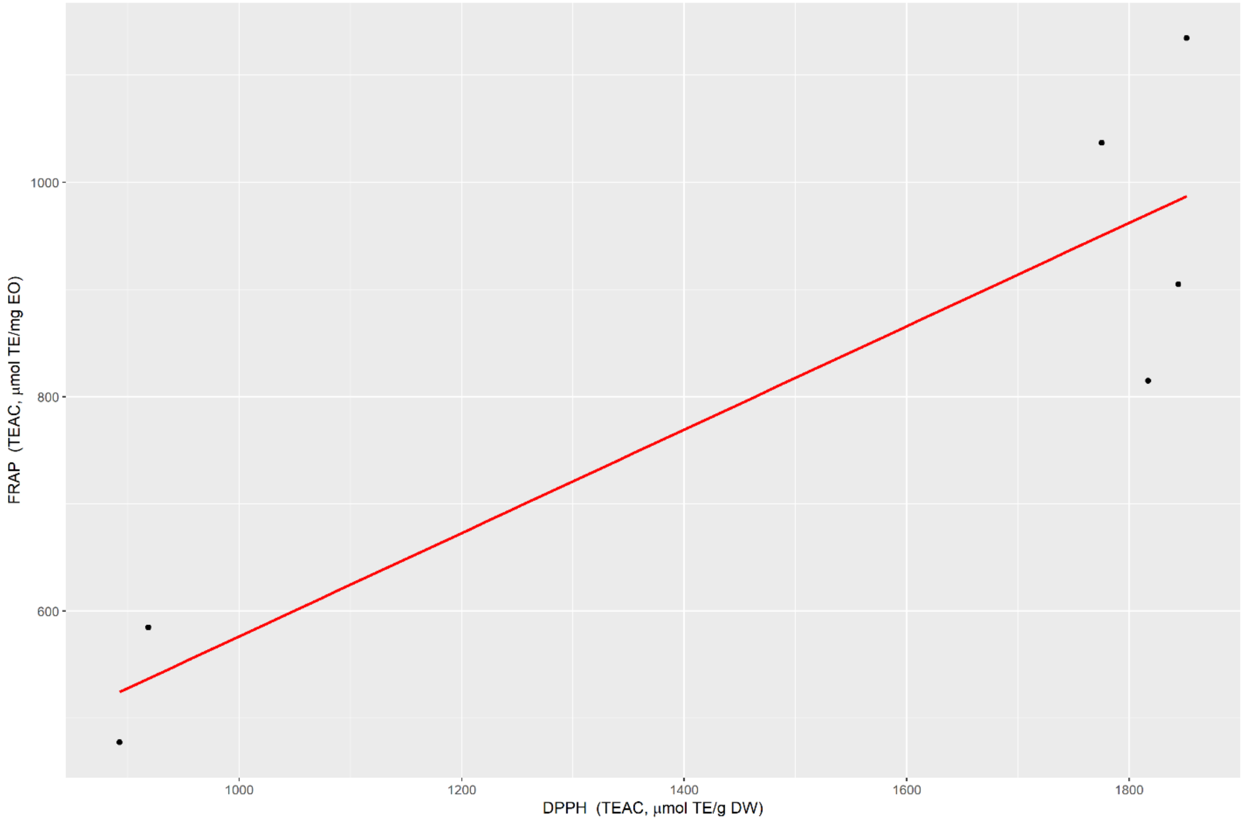

Table S28 and Figure S28. DPPH vs. FRAP (Ten *Pinus* taxa, [49])

Antioxidant endpoint	Test
1,065.00 ± 47.98	DPPH
1,161.66 ± 56.06	DPPH
990.31 ± 53.13	DPPH
1,272.75 ± 21.48	DPPH
1,141.79 ± 73.53	DPPH
499.15 ± 56.76	DPPH
1,155.45 ± 38.97	DPPH
1,263.20 ± 71.51	DPPH
1,128.44 ± 12.61	DPPH
1,118.03 ± 50.73	DPPH
441.18 ± 13.00	FRAP
418.63 ± 7.56	FRAP
406.09 ± 3.88	FRAP
1,677.19 ± 104.61	FRAP
1,007.24 ± 83.56	FRAP
370.81 ± 9.78	FRAP
564.64 ± 25.82	FRAP
1,286.11 ± 76.20	FRAP
1,007.62 ± 59.50	FRAP
956.63 ± 55.66	FRAP

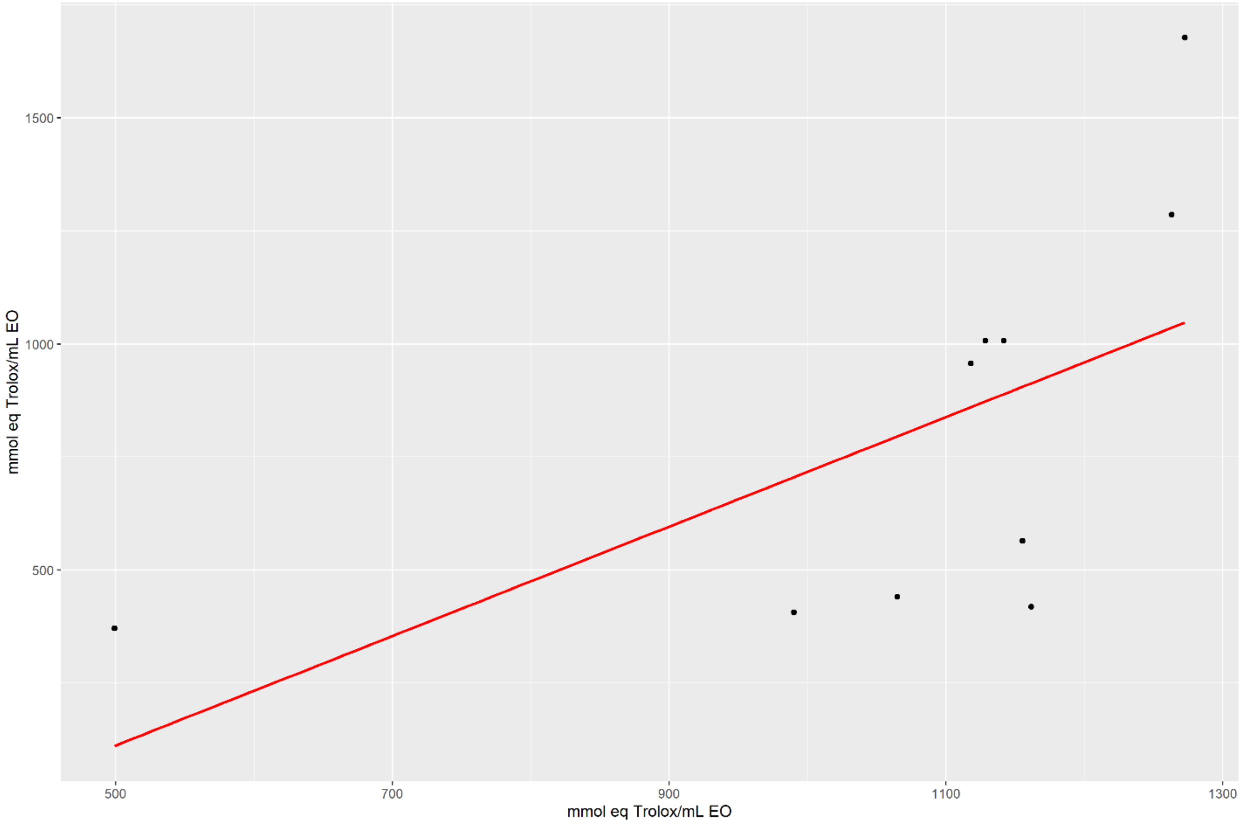

Table S29 and Figure S29. DPPH vs. Hydrogen peroxide scavenging (*Pinus roxburghii* Sarg., [62])

Antioxidant endpoint	Test
62.4 ± 0.41	DPPH
49.53 ± 0.11	DPPH
54.67 ± 0.38	DPPH
60.68 ± 0.34	DPPH
63.33 ± 0.47	DPPH
55.32 ± 0.29	Hydrogen_peroxide
52.49 ± 0.26	Hydrogen_peroxide
53.76 ± 0.16	Hydrogen_peroxide
50.43 ± 0.19	Hydrogen_peroxide
59.42 ± 0.32	Hydrogen_peroxide

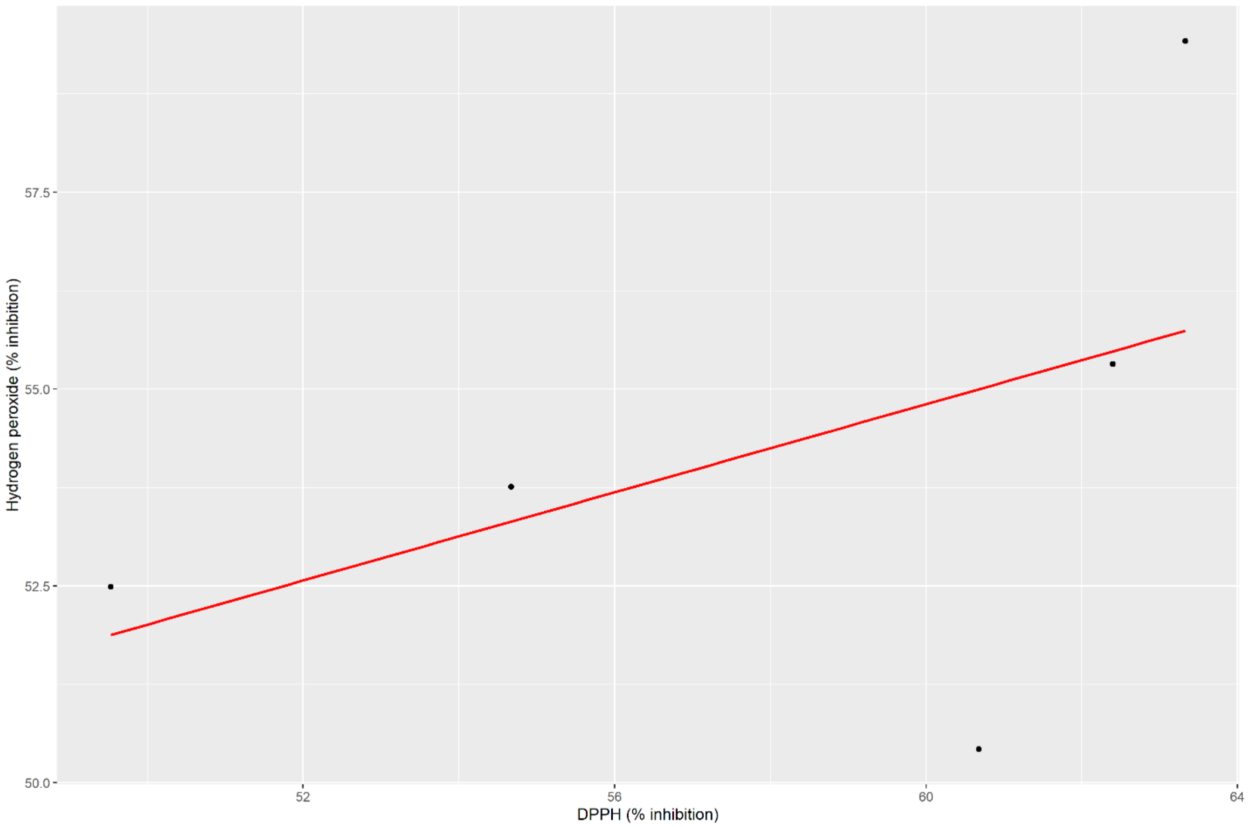

Table S30 and Figure S30. DPPH vs. Linoleic acid system (*Pinus roxburghii* Sarg., [62])

Antioxidant endpoint	Test
62.4 ± 0.41	DPPH
49.53 ± 0.11	DPPH
54.67 ± 0.38	DPPH
60.68 ± 0.34	DPPH
63.33 ± 0.47	DPPH
80.46 ± 1.42	Linoleic
88.37 ± 1.92	Linoleic
92.18 ± 1.33	Linoleic
93.16 ± 1.53	Linoleic
96.55 ± 1.71	Linoleic

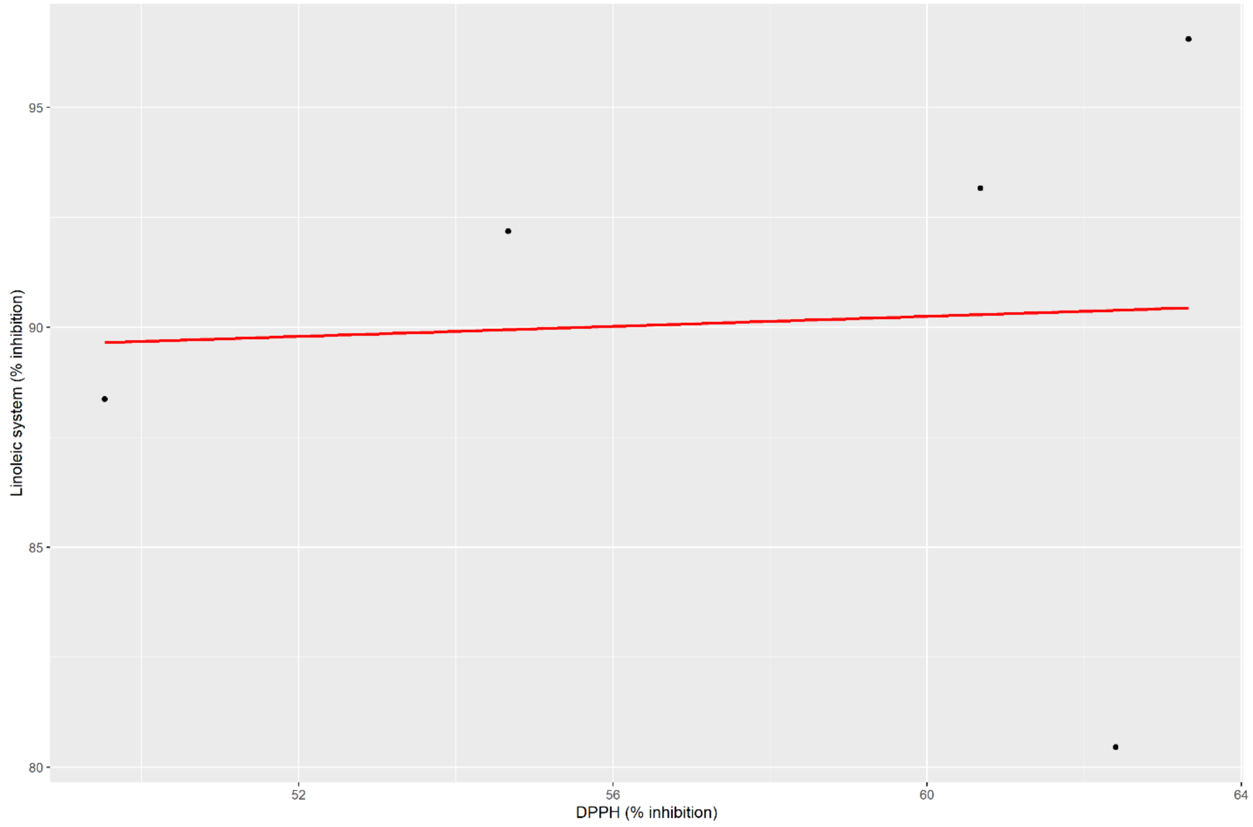

Table S31 and Figure S31. DPPH vs. Nitric oxide radical scavenging (*Pinus pinea* L., [101])

Antioxidant endpoint	Test
45.1 ± 1.5	DPPH
40.5 ± 0.7	DPPH
48.4 ± 1.2	DPPH
175.0 ± 2.5	Nitric_oxide
185.1 ± 1.9	Nitric_oxide
201.2 ± 1.7	Nitric_oxide

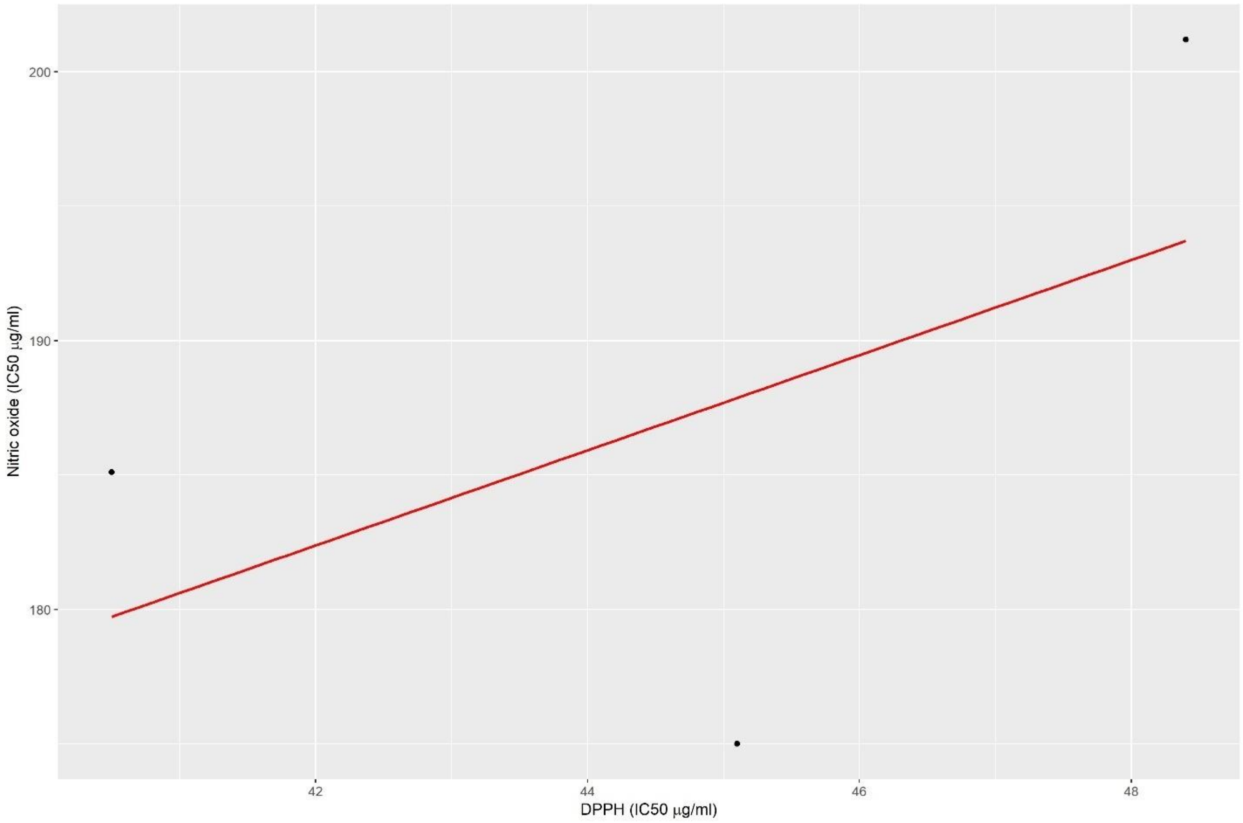

Table S32 and Figure S32. DPPH vs. OH-radical inhibition (*Pinus pinaster* Aiton, [72])

Antioxidant endpoint	Test
113.45	DPPH
85.82	DPPH
145.8	DPPH
138.25	OH radical
105.17	OH radical
152.27	OH radical

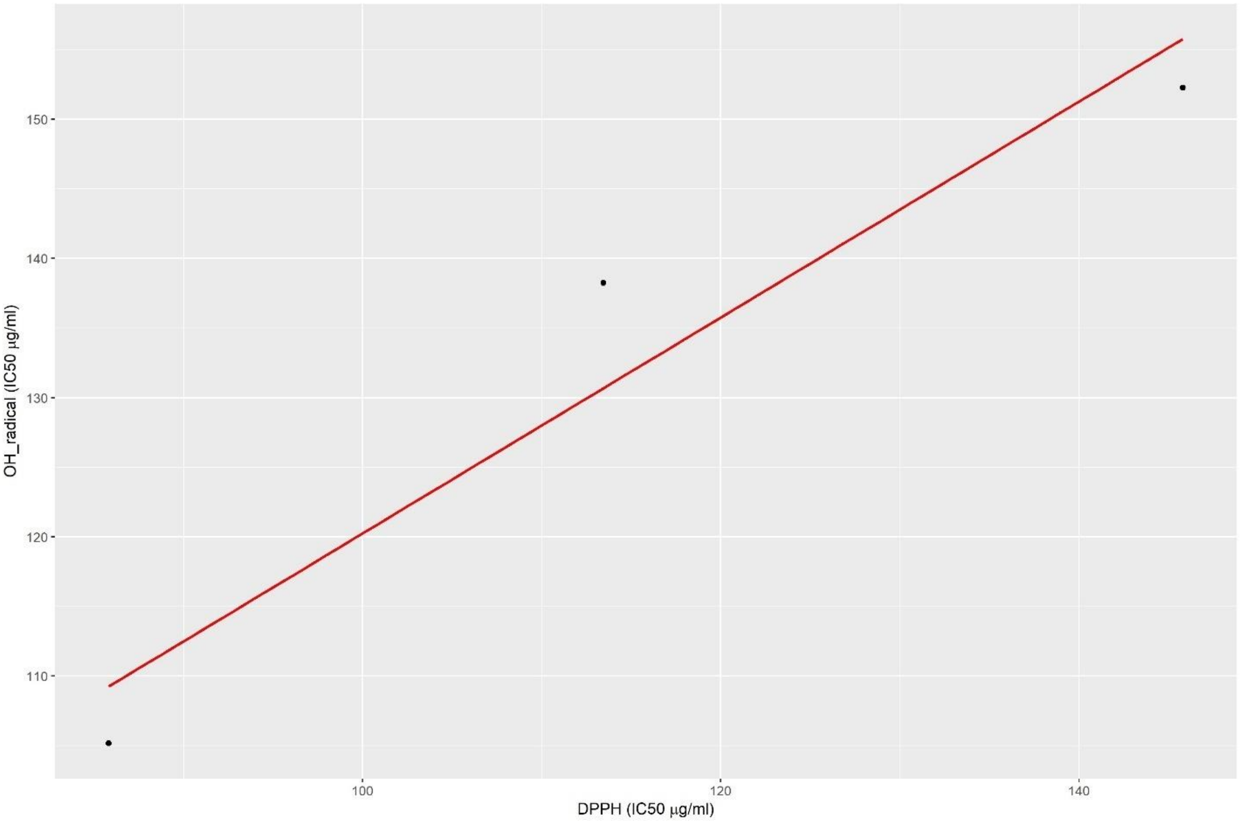

Table S33 and Figure S33. DPPH vs. reducing power (*Pinus halepensis* Mill., [69])

Antioxidant endpoint	Test
215.5172414	DPPH
212.6436782	DPPH
214.0804598	DPPH
213.71	DPPH
212.96	DPPH
238.5057471	DPPH
239.9425287	DPPH
244.2528736	DPPH
278.7356322	DPPH
284.12	DPPH
111.6883117	Reducing_power
107.7922078	Reducing_power
110.3896104	Reducing_power
111.6883117	Reducing_power
110.3896104	Reducing_power
141.5584416	Reducing_power
142.8571429	Reducing_power
144.1558442	Reducing_power
170.1298701	Reducing_power
172.7272727	Reducing_power

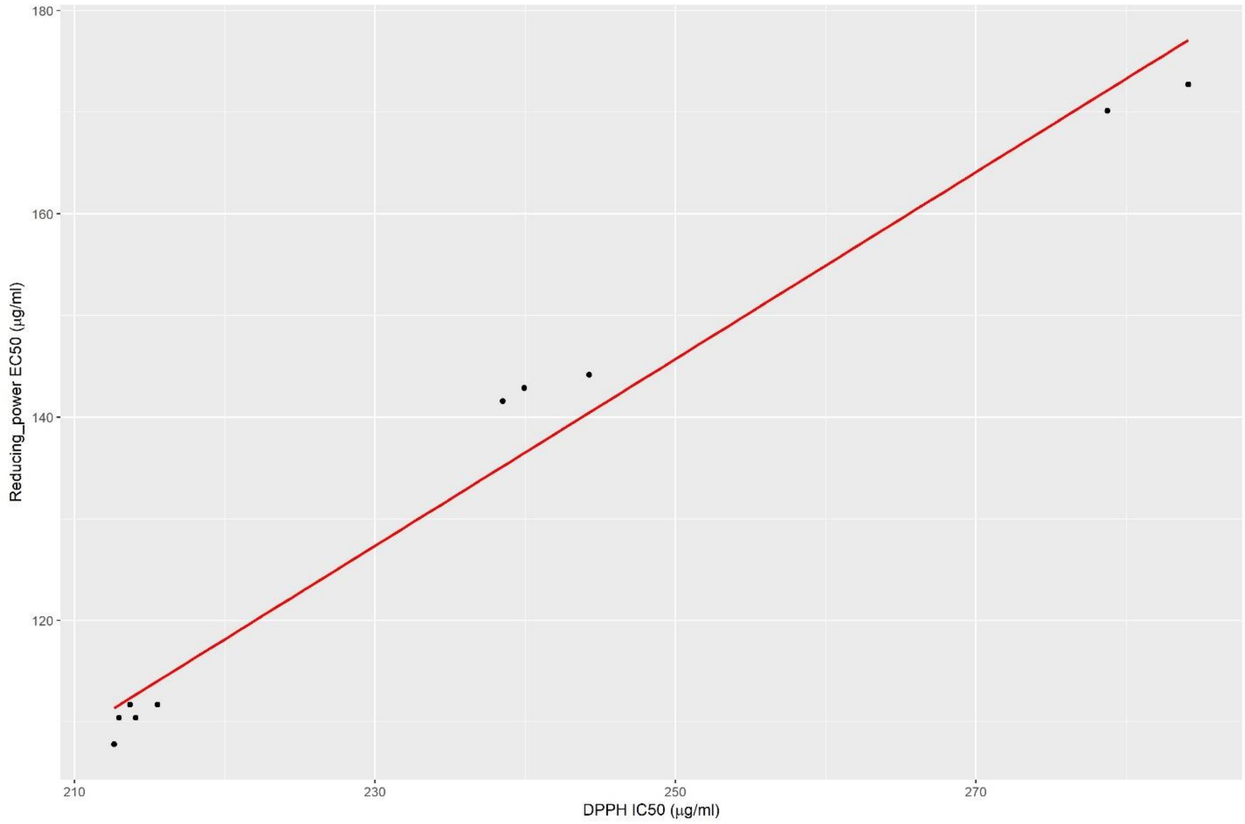

Table S34 and Figure S34. DPPH vs. reducing power (Pinus halepensis Mill., [81])

Antioxidant endpoint	Test
196.01 ± 2.32 c	DPPH
73.03 ± 5.44e	DPPH
114.24 ± 6.34d	DPPH
263.35 ± 13.00a	DPPH
203.10 ± 5.48bc	DPPH
132.69 ± 2.63d	DPPH
251.15 ± 9.26a	DPPH
270.86 ± 8.79a	DPPH
199.17 ± 4.23 c	DPPH
222.79 ± 15.61b	DPPH
191.09 ± 4.51 c	DPPH
1.36 ± 0.38 c	Reducing_power
1.64 ± 0.23 c	Reducing_power
1.54 ± 0.27 c	Reducing_power
3.92 ± 0.23a	Reducing_power
3.04 ± 0.30b	Reducing_power
1.41 ± 0.31 c	Reducing_power
1.30 ± 0.20 c	Reducing_power
1.98 ± 0.16 c	Reducing_power
1.51 ± 0.49 c	Reducing_power
3.05 ± 0.30bc	Reducing_power
1.23 ± 0.34 c	Reducing_power

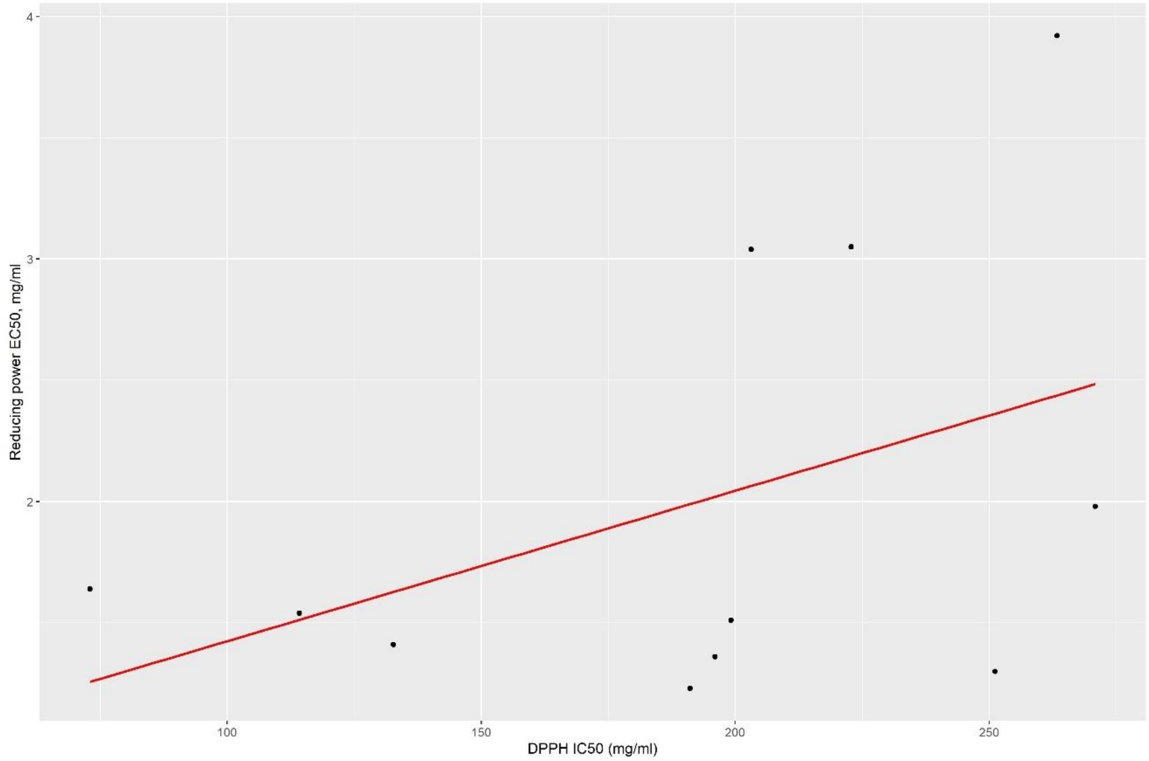

Table S35 and Figure S35. DPPH vs. TBARS (*Pinus mugo* Turro, [47])

Antioxidant endpoint	Test
2.65 ± 0.6 (year 1, 2039 m altitude)	DPPH
3.22 ± 0.4 (year 1, 1640 m altitude)	DPPH
2.51 ± 0.3 (year 2, 2039 m altitude)	DPPH
4.26 ± 0.5 (year 2, 1640 m altitude)	DPPH
2.59 ± 0.5 (year 1, 2039 m altitude)	TBARS
3.12 ± 0.6 (year 1, 1640 m altitude)	TBARS
2.42 ± 0.2 (year 2, 2039 m altitude)	TBARS
4.14 ± 0.3 (year 2, 1640 m altitude)	TBARS

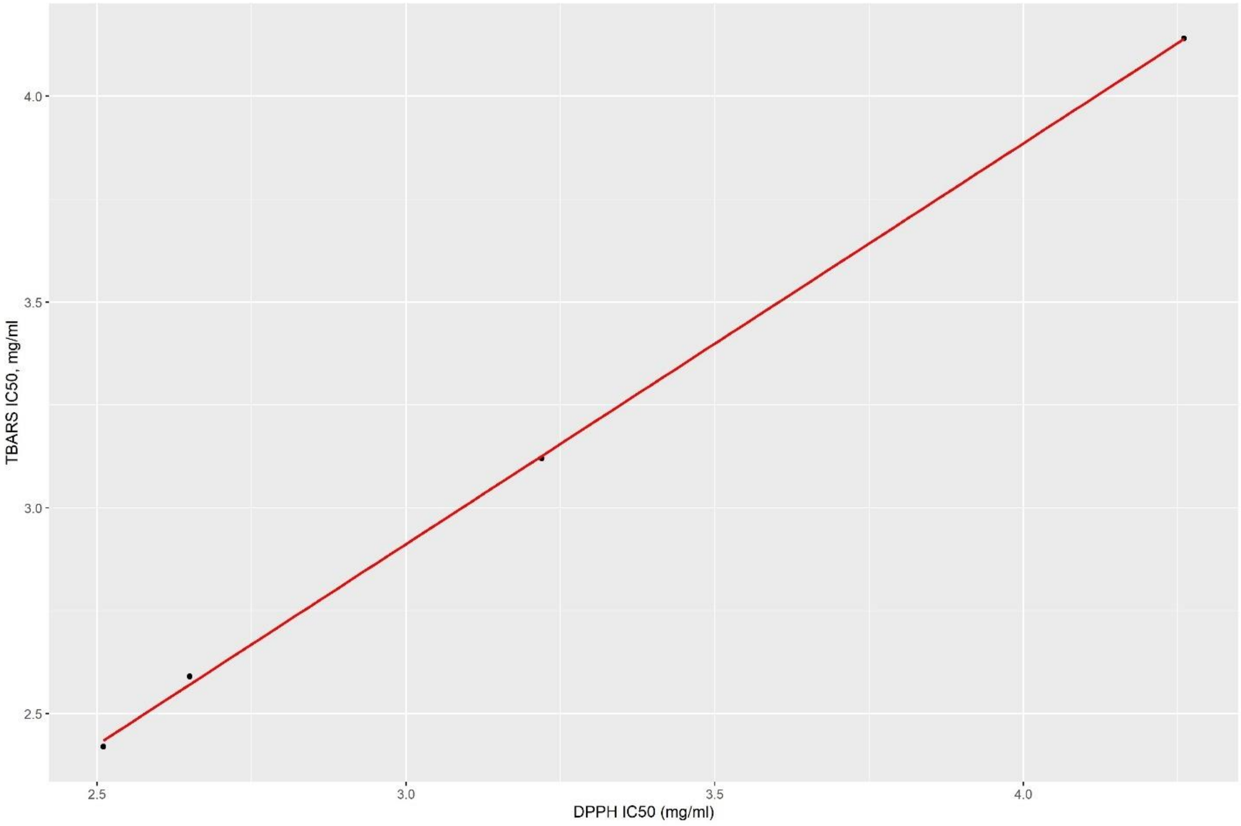

Table S36 and Figure S36. Ferrous ion-chelating vs. beta-carotene bleaching assay (*Pinus pinea* L. [101])

ANTIOXIDANT ENDPOINT	Test
51.1 ± 0.9	Iron_chelation
48.0 ± 1.1	Iron_chelation
55.0 ± 0.5	Iron_chelation
110.5 ± 1.3	Carotene_bleaching
115.4 ± 2.1	Carotene_bleaching
138.2 ± 2.3	Carotene_bleaching

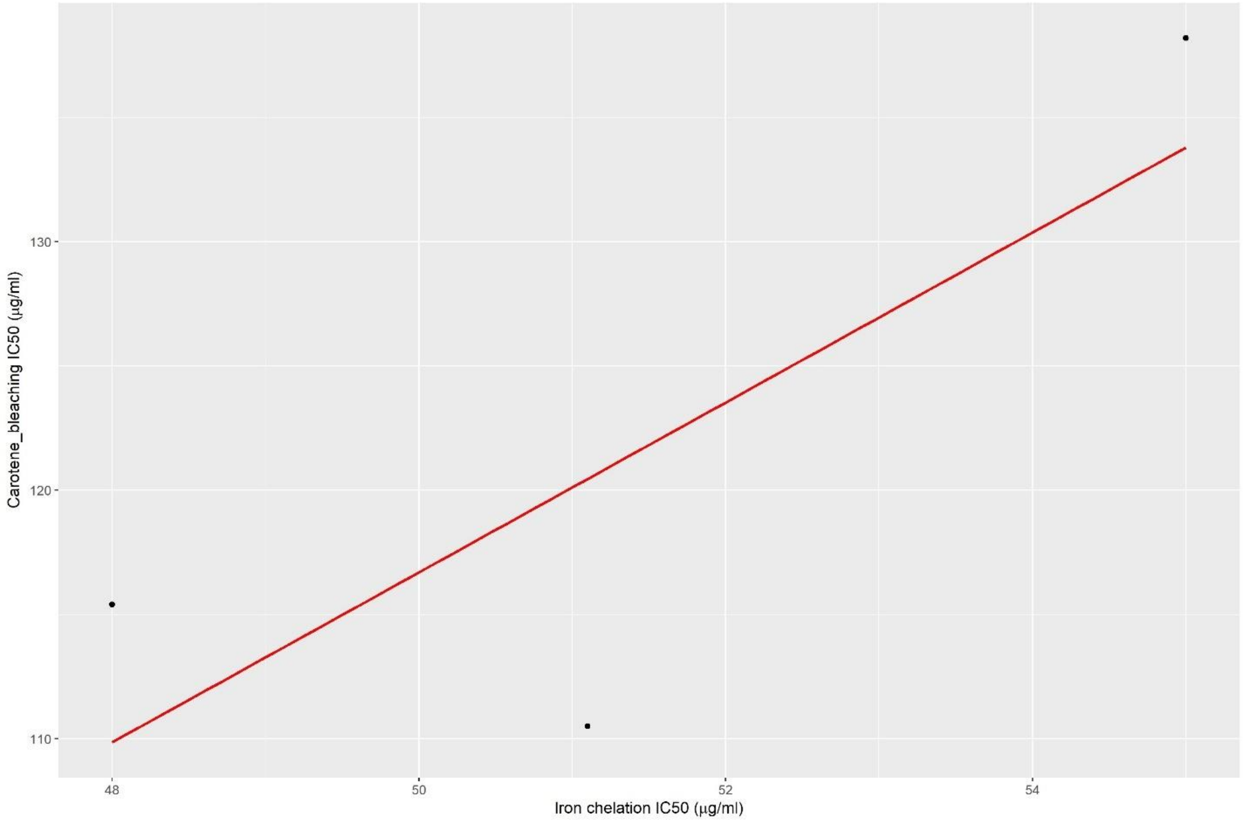

Table S37 and Figure S37. Ferrous ion-chelating vs. Nitric oxide radical scavenging (*Pinus pinea* L., [101])

Antioxidant endpoint	Test
51.1 ± 0.9	Iron_chelation
48.0 ± 1.1	Iron_chelation
55.0 ± 0.5	Iron_chelation
175.0 ± 2.5	Nitric_oxide
185.1 ± 1.9	Nitric_oxide
201.2 ± 1.7	Nitric_oxide

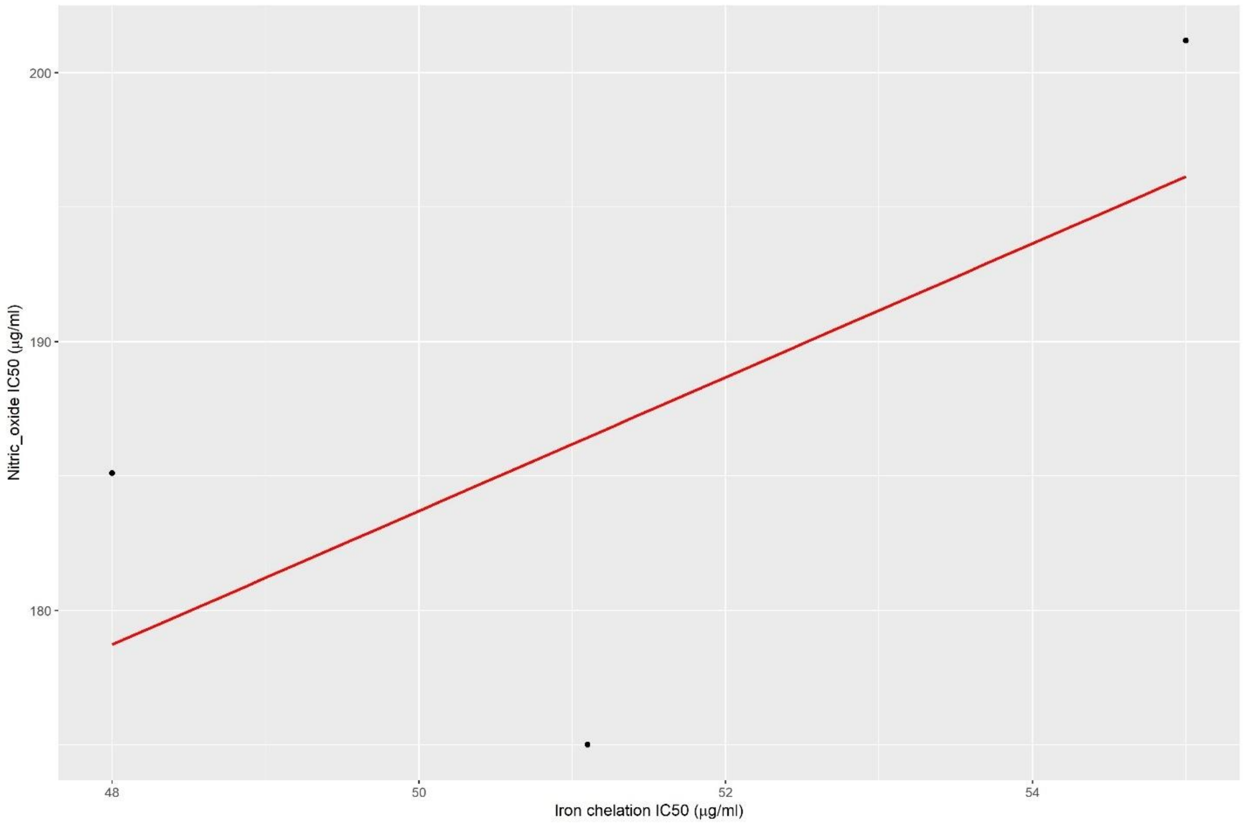

Table S38 and Figure S38. FRAP vs. OH (*Pinus pinaster* Aiton, [72])

ANTIOXIDANT ENDPOINT	Test
20.69	FRAP
27.92	FRAP
18.31	FRAP
138.25	Hydroxyl inhibition
105.17	Hydroxyl inhibition
152.27	Hydroxyl inhibition

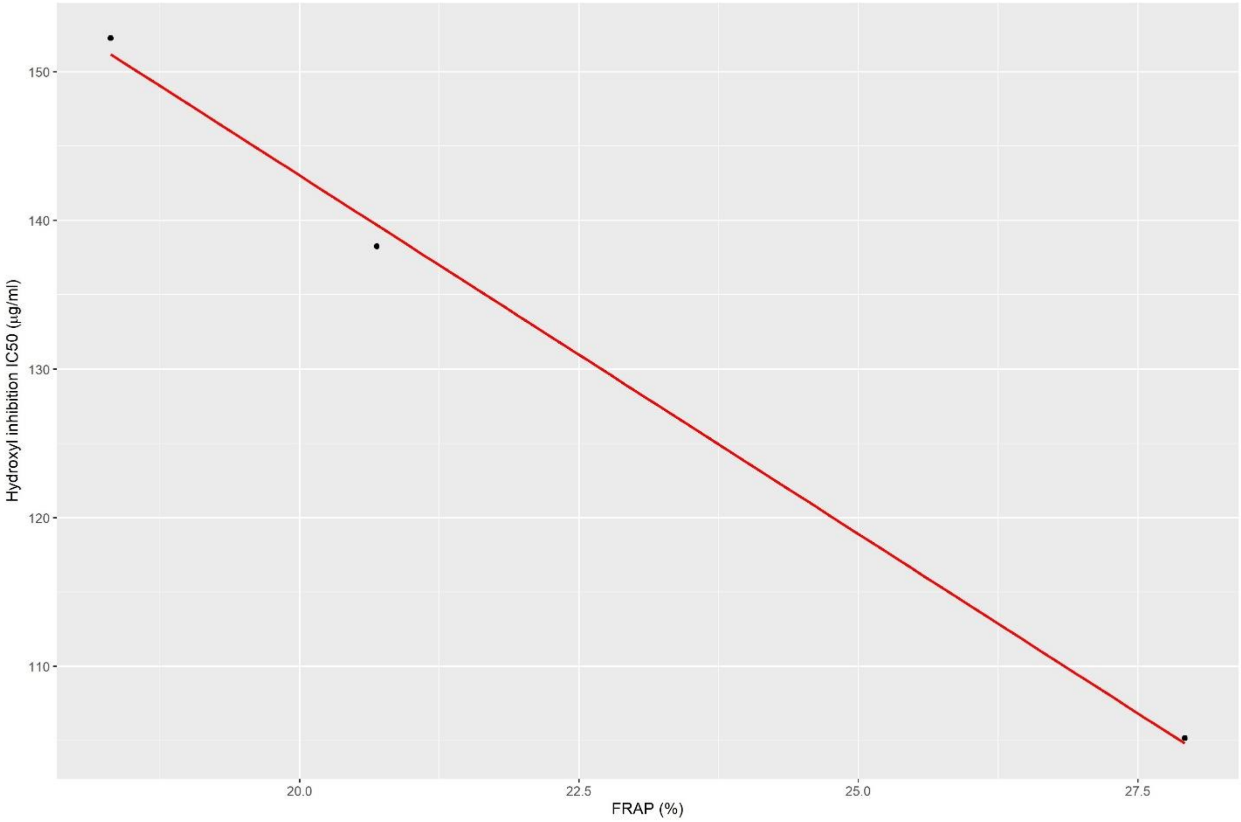

Table S39 and Figure S39. Hydrogen peroxide scavenging vs. FRAP (*Pinus roxburghii* Sarg., [62])

ANTIOXIDANT ENDPOINT	Test
55.32 ± 0.29	Hydrogen_peroxide
52.49 ± 0.26	Hydrogen_peroxide
53.76 ± 0.16	Hydrogen_peroxide
50.43 ± 0.19	Hydrogen_peroxide
59.42 ± 0.32	Hydrogen_peroxide
67.44 ± 0.95	FRAP
99.92 ± 1.06	FRAP
102.07 ± 1.12	FRAP
127.12 ± 1.21	FRAP
134.49 ± 1.34	FRAP

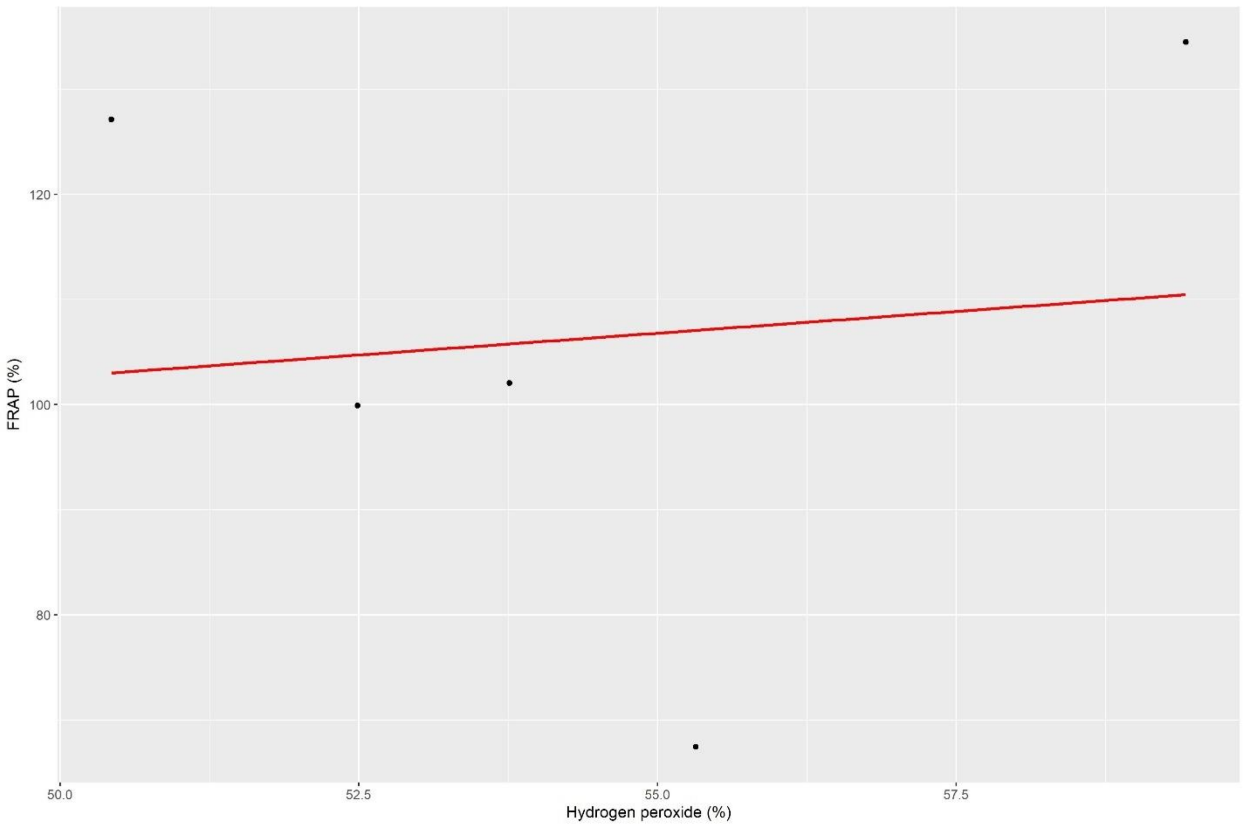

Table S40 and Figure S40. Linoleic acid system vs. FRAP (*Pinus roxburghii* Sarg., [62])

ANTIOXIDANT ENDPOINT	Test
80.46 ± 1.42	Linoleic_system
88.37 ± 1.92	Linoleic_system
92.18 ± 1.33	Linoleic_system
93.16 ± 1.53	Linoleic_system
96.55 ± 1.71	Linoleic_system
67.44 ± 0.95	FRAP
99.92 ± 1.06	FRAP
102.07 ± 1.12	FRAP
127.12 ± 1.21	FRAP
134.49 ± 1.34	FRAP

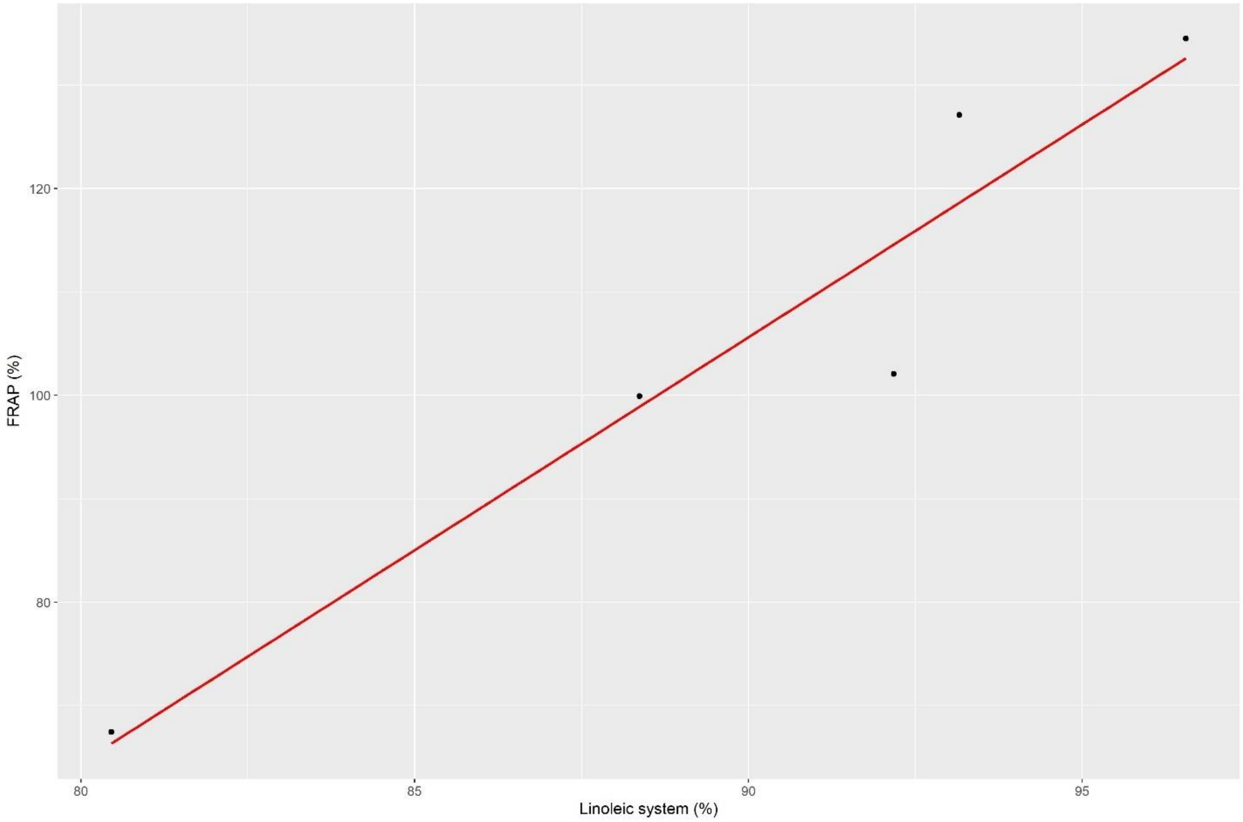

Table S41 and Figure S41. Linoleic acid system vs. Hydrogen peroxide scavenging (*Pinus roxburghii* Sarg., [62])

ANTIOXIDANT ENDPOINT	Test
80.46 ± 1.42	Linoleic_system
88.37 ± 1.92	Linoleic_system
92.18 ± 1.33	Linoleic_system
93.16 ± 1.53	Linoleic_system
96.55 ± 1.71	Linoleic_system
55.32 ± 0.29	Hydrogen_peroxide
52.49 ± 0.26	Hydrogen_peroxide
53.76 ± 0.16	Hydrogen_peroxide
50.43 ± 0.19	Hydrogen_peroxide
59.42 ± 0.32	Hydrogen_peroxide

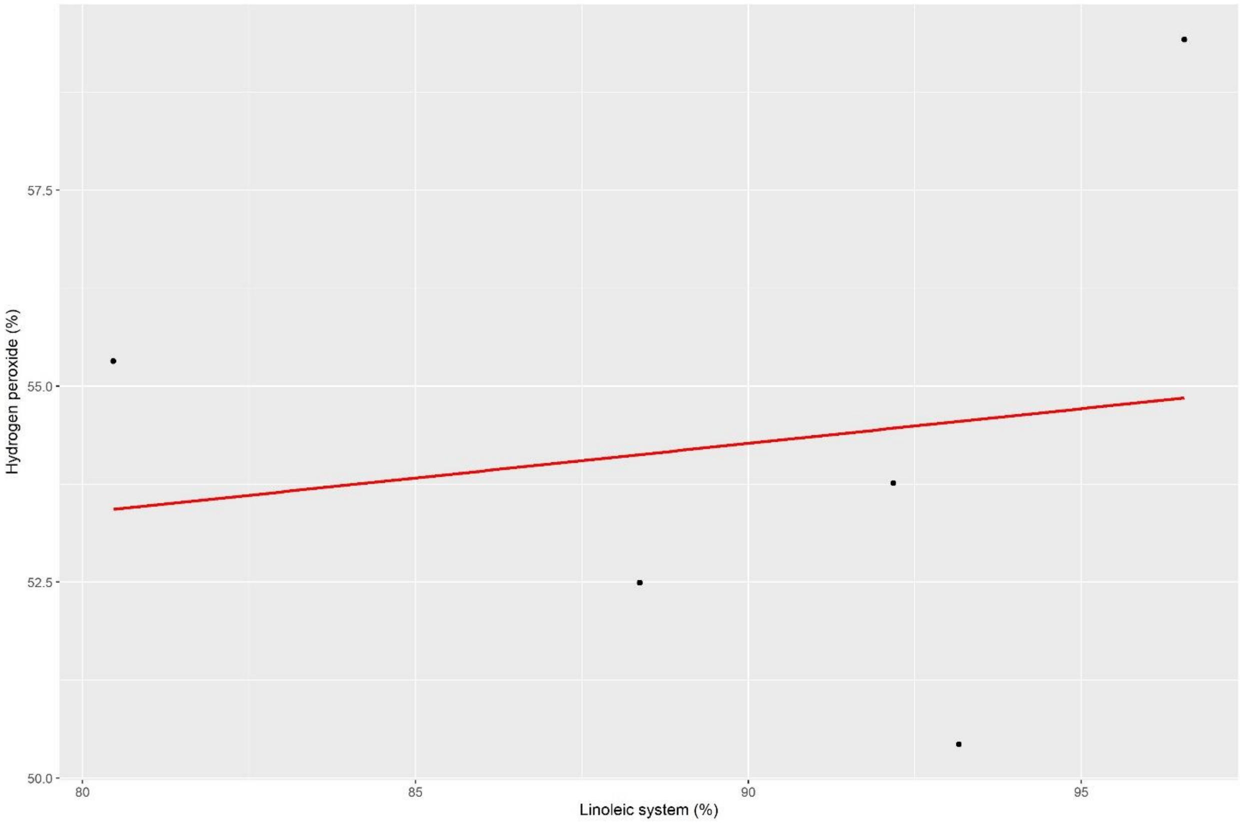

Table S42 and Figure S42. Reducing power vs. Beta-carotene bleaching (*Pinus halepensis* Mill., [69])

ANTIOXIDANT ENDPOINT	Test
111.6883117	Reducing_power
107.7922078	Reducing_power
110.3896104	Reducing_power
111.6883117	Reducing_power
110.3896104	Reducing_power
141.5584416	Reducing_power
142.8571429	Reducing_power
144.1558442	Reducing_power
170.1298701	Reducing_power
172.7272727	Reducing_power
52.21932115	Carotene_bleaching
52.21932115	Carotene_bleaching
53.52480418	Carotene_bleaching
49.60835509	Carotene_bleaching
52.21932115	Carotene_bleaching
67.88511749	Carotene_bleaching
71.80156658	Carotene_bleaching
71.80156658	Carotene_bleaching
84.85639687	Carotene_bleaching
96.60574413	Carotene_bleaching

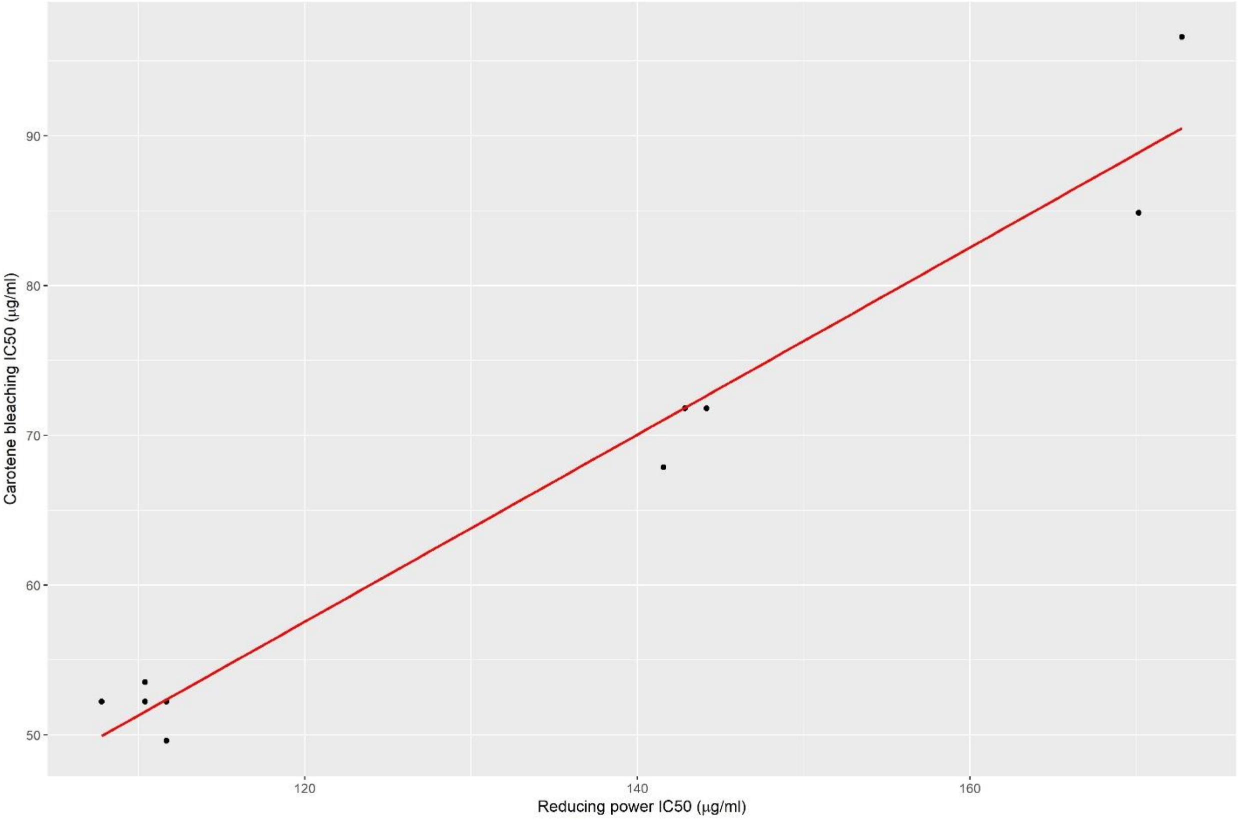

Table S43 and Figure S43. Reducing power vs. Ferrous ion-chelating activity (*Pinus halepensis* Mill., [69])

ANTIOXIDANT ENDPOINT	Test
111.6883117	Reducing_power
107.7922078	Reducing_power
110.3896104	Reducing_power
111.6883117	Reducing_power
110.3896104	Reducing_power
141.5584416	Reducing_power
142.8571429	Reducing_power
144.1558442	Reducing_power
170.1298701	Reducing_power
172.7272727	Reducing_power
354.1666667	Iron_chelation
352.8645833	Iron_chelation
355.46875	Iron_chelation
212.2395833	Iron_chelation
355.46875	Iron_chelation
389.3229167	Iron_chelation
385.4166667	Iron_chelation
243.4895833	Iron_chelation
425.78125	Iron_chelation
424.4791667	Iron_chelation

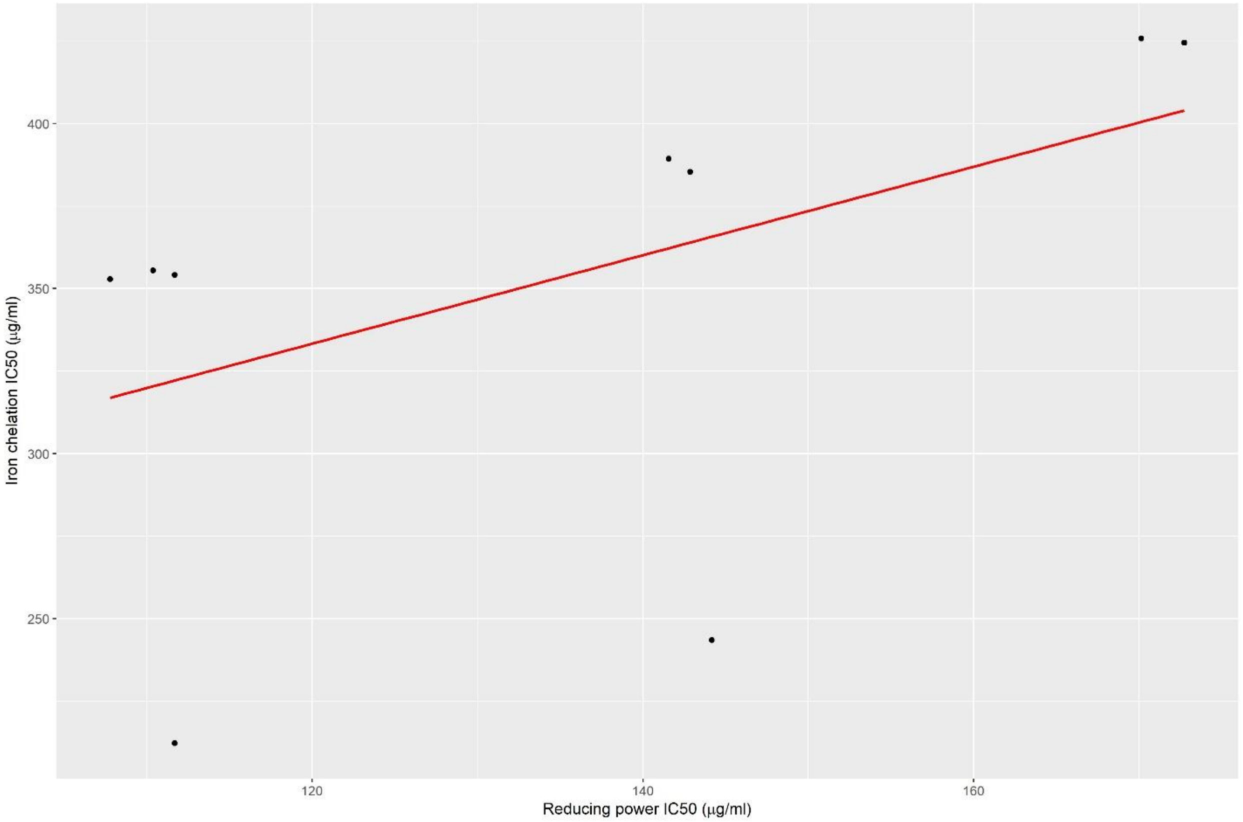

For 44-46: TPC vs. ABTS, TPC vs. DPPH, and TPC vs. FRAP (Ten *Pinus* taxa, [49]), correlations were computed and reported directly by the source paper.
